# Co-expression-based models improve eQTL predictions for transcriptome-wide association studies and highlight new schizophrenia-associated genes

**DOI:** 10.1038/s41588-026-02646-3

**Published:** 2026-06-22

**Authors:** Fabiana Rossi, Leonardo Sportelli, Gianluca C. Kikidis, Giulia Grassi, Fabio Di Camillo, Alessandro Bertolino, Giuseppe Blasi, Christopher J. Borcuk, Daniela Fusco, Thomas M. Hyde, Joel E. Kleinman, Davide Marnetto, Silvia Pellegrini, Antonio Rampino, Benedetto Vitiello, Stephan Ripke, Alice Braun, Julia Kraft, Sintia Iole Belangero, Paulo R. Menezes, Celso Arango, James T. R. Walters, Michael C. O’Donovan, Michael J. Owen, David Braff, Aiden Corvin, Derek W. Morris, Enrico Domenici, Jim van Os, Esref Atbaşoğlu, Meram C. Saka, Marta Di Forti, Bernhard T. Baune, Carlos N. Pato, Andrew McQuillin, Vera Golimbet, Nikolay Kondratyev, Valentina Escott-Price, Anna Gareeva, Elza Khusnutdinova, Jorge A. Cervilla, Margarita Rivera, Dominique Campion, Claudine Laurent-Levinson, Alessandro Serretti, Ole A. Andreassen, David St. Clair, Todd Lencz, Anil K. Malhotra, Nina S. McCarthy, Bryan J. Mowry, Dan Rujescu, Ina Giegling, Annette M. Hartmann, Bettina Konte, Markus M. Nöthen, Marcella Rietschel, George Kirov, Patrick F. Sullivan, Tracey L. Petryshen, Thomas Werge, Andrew M. McIntosh, Tõnu Esko, Erik G. Jönsson, Hannelore Ehrenreich, Brien P. Riley, Douglas F. Levinson, Joseph D. Buxbaum, Elvira Bramon, Christina M. Hultman, Roel A. Ophoff, Rolf Adolfsson, Eli A. Stahl, Sinan Guloksuz, Bart P. F. Rutten, Cristina M. Del-Ben, Florence Thibaut, Daniel R. Weinberger, Giulio Pergola

**Affiliations:** 1 Department of Translational Biomedicine and Neuroscience, University of Bari Aldo Moro, Bari, Italy; 2 Lieber Institute for Brain Development, Johns Hopkins Medical Campus, Baltimore, MD USA; 3 IMT School for Advanced Studies Lucca, Lucca, Italy; 4 Department of Clinical and Experimental Medicine, University of Pisa, Pisa, Italy; 5 Psychiatric Unit, Bari University Hospital, Bari, Italy; 6 Department of Neurosciences “Rita Levi Montalcini”, University of Turin, Turin, Italy; 7 Department of Psychiatry and Behavioral Sciences, Johns Hopkins University School of Medicine, Baltimore, MD USA; 8 Department of Neurology, Johns Hopkins University School of Medicine, Baltimore, MD USA; 9 Department of Public Health and Pediatric Sciences, University of Turin, Turin, Italy; 10 Department of Psychiatry and Psychotherapy, Charité - Universitätsmedizin, Berlin, Germany; 11 Stanley Center for Psychiatric Research, Broad Institute of MIT and Harvard, Cambridge, MA USA; 12 Analytic and Translational Genetics Unit, Massachusetts General Hospital, Boston, MA USA; 13 German Center for Mental Health (DZPG), partner site Berlin/Potsdam, Berlin, Germany; 14 Department of Morphology and Genetics, Laboratorio de Genetica, Universidade Federal de São Paulo, São Paulo, Brazil; 15 Laboratory of Integrative Neuroscience, Universidade Federal de São Paulo, São Paulo, Brazil; 16 Department of Preventative Medicine, Faculdade de Medicina FMUSP, University of São Paulo, São Paulo, Brazil; 17 Hospital Universitario La Paz, IdiPAZ, School of Medicine, Universidad Autónoma de Madrid, Madrid, Spain; 18 Centro de Investigación Biomédica en Red en Salud Mental (CIBERSAM), Madrid, Spain; 19 Centre for Neuropsychiatric Genetics and Genomics, Division of Psychological Medicine and Clinical Neurosciences, Cardiff University, Cardiff, UK; 20 Department of Psychiatry, University of California San Diego, La Jolla, CA USA; 21 VISN 22, Mental Illness Research, Education and Clinical Center (MIRECC), VA San Diego Healthcare System, San Diego, CA USA; 22 Neuropsychiatric Genetics Research Group, Department of Psychiatry, Trinity College Dublin, Dublin, Ireland; 23 Centre for Neuroimaging, Cognition and Genomics (NICOG), School of Biological and Chemical Sciences, University of Galway, Galway, Ireland; 24 Department of Cellular, Computational and Integrative Biology, University of Trento, Trento, Italy; 25 University Medical Center Utrecht, Department of Psychiatry, Utrecht, Netherlands; 26 Department of Psychosis Studies, Institute of Psychiatry, Psychology and Neuroscience, King’s College London, London, UK; 27 Department of Psychiatry, School of Medicine, Ankara University, Ankara, Turkey; 28 Department of Genetics and Genomics, Icahn School of Medicine at Mount Sinai, New York, NY USA; 29 Social, Genetic and Developmental Psychiatry Centre, Institute of Psychiatry, Psychology and Neuroscience, King’s College London, London, UK; 30 National Institute for Health Research (NIHR) Maudsley Biomedical Research Centre at South London and Maudsley NHS Foundation Trust and King’s College London, London, UK; 31 South London and Maudsley NHS Mental Health Foundation Trust, London, UK; 32 Department of Psychiatry, University of Münster, Münster, Germany; 33 Department of Psychiatry, Melbourne Medical School, University of Melbourne, Parkville, Victoria Australia; 34 The Florey Institute of Neuroscience and Mental Health, University of Melbourne, Parkville, Victoria Australia; 35 Rutgers University, Robert Wood Johnson Medical School, New Brunswick, NJ USA; 36 Department of Psychiatry and Zilkha Neurogenetics Institute, Keck School of Medicine at University of Southern California, Los Angeles, CA USA; 37 Molecular Psychiatry Laboratory, Division of Psychiatry, University College London, London, UK; 38 Russian Mental Health Research Center, Moscow, Russia; 39 Dementia Research Institute, Cardiff University, Cardiff, UK; 40 Bashkir State Medical University, Ufa, Russia; 41 Institute of Biochemistry and Genetics, Ufa Federal Research Center of the Russian Academy of Sciences, Ufa, Russia; 42 Department of Psychiatry, San Cecilio University Hospital, University of Granada, Granada, Spain; 43 Department of Biochemistry and Molecular Biology II, Faculty of Pharmacy, University of Granada, Granada, Spain; 44 Institute of Neurosciences ´Federico Olóriz´, Biomedical Research Centre (CIBM), University of Granada, Granada, Spain; 45 Instituto de Investigación Biosanitaria, Ibs Granada, Granada, Spain; 46 INSERM, Rouen, France; 47 Centre Hospitalier du Rouvray, Rouen, France; 48 Department of Child and Adolescent Psychiatry, Reference Center for Rare Disease with Psychiatric Expression, Pitié-Salpêtrière University Hospital, Assistance Publique-Hôpitaux de Paris, Paris, France; 49 UMRs933-Childhood Genetic Diseases Laboratory, INSERM and Sorbonne University, Trousseau Hospital, Paris, France; 50 Department of Medicine and Surgery, Kore University of Enna, Enna, Italy; 51 Oasi Research Institute-IRCCS, Troina, Italy; 52 Centre for Precision Psychiatry, Division of Mental Health and Addiction, University of Oslo, Oslo, Norway; 53 Division of Mental Health and Addiction, Oslo University Hospital, Oslo, Norway; 54 Institute of Medical Sciences, University of Aberdeen, Aberdeen, UK; 55 Division of Psychiatry Research, Zucker Hillside Hospital, Glen Oaks, NY USA; 56 Institute of Behavioral Science, Feinstein Institutes for Medical Research, Manhasset, NY USA; 57 Department of Psychiatry, Zucker School of Medicine at Hofstra/Northwell, Hempstead, NY USA; 58 School of Biomedical Sciences, The University of Western Australia, Perth, Western Australia Australia; 59 Queensland Brain Institute, University of Queensland, Brisbane, Queensland Australia; 60 Queensland Centre for Mental Health Research, The University of Queensland, Brisbane, Queensland Australia; 61 Department of Psychiatry and Psychotherapy, Comprehensive Center for Clinical Neurosciences and Mental Health (C3NMH), Medical University of Vienna, Vienna, Austria; 62 Institute of Human Genetics, University of Bonn & University Hospital of Bonn, Bonn, Germany; 63 Department of Genetic Epidemiology in Psychiatry, Central Institute of Mental Health, Medical Faculty Mannheim, University of Heidelberg, Mannheim, Germany; 64 Department of Genetics, University of North Carolina, Chapel Hill, NC USA; 65 Department of Psychiatry, University of North Carolina, Chapel Hill, NC USA; 66 Department of Medical Epidemiology and Biostatistics, Karolinska Institutet, Stockholm, Sweden; 67 Psychiatric and Neurodevelopmental Genetics Unit, Department of Psychiatry, Massachusetts General Hospital, Harvard Medical School, Boston, MA USA; 68 Institute of Biological Psychiatry, Mental Health Services, Copenhagen University Hospital, Copenhagen, Denmark; 69 Department of Clinical Medicine, University of Copenhagen, Copenhagen, Denmark; 70 Center for GeoGenetics, University of Copenhagen, Copenhagen, Denmark; 71 iPSYCH, The Lundbeck Foundation Initiative for Integrative Psychiatric Research, Copenhagen, Denmark; 72 Institute of Neuroscience and Cardiovascular Research, University of Edinburgh, Edinburgh, UK; 73 Institute of Genomics, University of Tartu, Tartu, Estonia; 74 Centre for Psychiatry Research, Department of Clinical Neuroscience, Karolinska Institutet and Stockholm Health Care Services, Stockholm Region, Stockholm, Sweden; 75 NORMENT Centre, Institute of Clinical Medicine, University of Oslo, Oslo, Norway; 76 Clinical Neuroscience, Max Planck Institute of Experimental Medicine, Göttingen, Germany; 77 Central Institute of Mental Health, Medical Faculty Mannheim, University of Heidelberg, Mannheim, Germany; 78 Virginia Institute for Psychiatric and Behavioral Genetics, Department of Psychiatry, Virginia Commonwealth University, Richmond, VA USA; 79 Department of Psychiatry and Behavioral Sciences, Stanford University, Stanford, CA USA; 80 Department of Psychiatry, Icahn School of Medicine at Mount Sinai, New York, NY USA; 81 Division of Psychiatry, Department of Mental Health Neuroscience, University College London, London, UK; 82 Institute of Cognitive Neuroscience, University College London, London, UK; 83 Center for Neurobehavioral Genetics, Semel Institute for Neuroscience and Human Behavior, University of California, Los Angeles, CA USA; 84 Department of Human Genetics, David Geffen School of Medicine, University of California Los Angeles, Los Angeles, CA USA; 85 Department of Clinical Sciences, Psychiatry, Umeå University, Umeå, Sweden; 86 Program in Medical and Population Genetics, The Broad Institute of MIT and Harvard, Cambridge, MA USA; 87 Regeneron Genetics Center, Tarrytown, NY USA; 88 Department of Psychiatry, University of British Columbia, Vancouver, British Columbia Canada; 89 Institute of Mental Health, University of British Columbia, Vancouver, British Columbia Canada; 90 Department of Psychiatry and Neuropsychology, School for Mental Health and Neuroscience, Maastricht University Medical Centre, Maastricht, The Netherlands; 91 Department of Psychiatry, Yale University School of Medicine, New Haven, CT USA; 92 Neuroscience and Behavior Department; Ribeirão Preto Medical School, University of São Paulo, São Paulo, Brazil; 93 University Paris Cité, Hôpital Cochin-Tarnier, Paris, France; 94 INSERM U1266, Institute of Psychiatry and Neurosciences, Paris, France; 95 Department of Neuroscience, Johns Hopkins University School of Medicine, Baltimore, MD USA; 96 Department of Genetic Medicine, Johns Hopkins University School of Medicine, Baltimore, MD USA

**Keywords:** Schizophrenia, Gene expression profiling

## Abstract

Most genetic variants associated with complex heritability phenotypes lie in non-coding regions and are thought to influence disease risk by regulating gene expression. However, most transcriptome-wide association approaches primarily model local (*cis*) genetic effects, leaving much of gene regulation unexplained. Here, we show that incorporating distal (*trans*) regulatory effects improves the prediction of gene expression and the identification of disease-associated genes. Using RNA sequencing data from six human post-mortem brain regions, we developed INGENE and MODULE, two models capturing the combined influence of candidate *trans-*acting variants within gene coexpression networks. Integrating these models with conventional *cis*-based predictors improved gene expression imputation (maximum likelihood estimation, α = 0.05) for 18,744 genes across regions. Applying this framework to Psychiatric Genomics Consortium wave 3 genotypes identified 766 genes associated with schizophrenia (*P*_FDR_ < 0.01), including 641 not previously reported by transcriptome-wide analyses. These findings highlight the contribution of distal regulatory mechanisms and gene network interactions to schizophrenia risk.

## Abstract (web summary)

INGENE and MODULE enhance gene expression prediction by capturing the effects of *trans*-eQTLs acting within coexpression networks. Integrating *cis* and *trans* predictions improves expression imputation for transcriptome-wide association studies.

## Main

Schizophrenia (SCZ) is a severe psychiatric disorder with twin-based heritability estimates between 60% and 80%^1,2^. Genome-wide association studies (GWAS) have identified numerous common single nucleotide polymorphisms (SNPs) associated with SCZ risk. Although increasing GWAS sample sizes have enhanced the number of significant loci^3,4^, the explained heritability remains limited, and translating these statistical associations into molecular mechanisms remains challenging^5^. Most risk variants are non-coding^2^ and are probably associated with gene expression^6,7^.

Gene expression provides a functional intermediary between the DNA sequence and disease liability^6,8^. Accordingly, transcriptome-wide association studies (TWAS)^9^ integrate GWAS with expression quantitative trait loci (eQTL) reference panels to impute the genetic component of gene expression and test its association with phenotypes^9–14^. In TWAS, two main approaches are commonly used: the direct prediction of expression in genotyped samples (gene-level TWAS) and the indirect estimation of associations between predicted expression and traits, accounting for linkage disequilibrium among SNPs (SNP-level TWAS). Both approaches identify gene-level trait associations while reducing the multiple-testing burden compared to SNP-based GWAS^11,12^. However, most existing TWAS implementations, including PrediXcan^11^ and related methods^12,15,16^, mainly rely on *cis*-eQTLs (variants within ±1 Mb of a gene). This assumption limits interpretability, as most non-coding risk variants do not colocalize with known eQTLs^6,17–19^, and *cis* effects explain only a fraction of expression heritability^2,20–26^.

*Trans* regulation may be particularly relevant in SCZ, whereby risk variants act through coordinated transcriptional programs across brain regions and developmental stages^27,28^. As *trans* effects are weaker than *cis* effects, their detection requires larger sample sizes^11^, and systematic characterization remains challenging^17^.

Nonetheless, risk-associated variants are thought to converge on coordinated transcriptional programs^28^ across brain regions, over time^29,30^ and even at the cell-type level^31–33^. Several studies have reported that SCZ-associated genes converge within coexpression networks^33–39^, suggesting that shared variance among sets of genes may reflect underlying regulatory architecture and disease-relevant biology, and these findings can be validated in vitro^39,40^.

Several studies have leveraged SNPs proximal to genes in coexpressed sets, termed coexpression-based eQTLs (co-eQTLs), to generate system-level polygenic scores to translate coexpression into phenotypic predictions^15,38,41–47^. These approaches leverage gene modules or coexpression partners to prioritize variants that may exert coordinated regulatory effects^39,48,49^. Given that it limits the search space to gene sets, this approach to identifying *trans*-eQTLs differs from genome-wide scans, which are often underpowered owing to the burden of multiple comparisons^11^.

Here, we developed two predictive frameworks to quantify *trans*-regulatory contributions to gene-level expression: the imputed network gene-expression *trans*-eQTL (INGENE), which models the *trans* effects of *cis*-regulated coexpression partners on a target gene; and the module quantitative trait loci eigengene (MODULE), which identifies SNPs associated with the eigenvalue (first principal component) of a gene’s coexpression module. In addition, we implemented two *cis*-based benchmarks: a standard elastic-net model^11^ (CIS) and EpiXcan^12^.

**Fig. 1:**
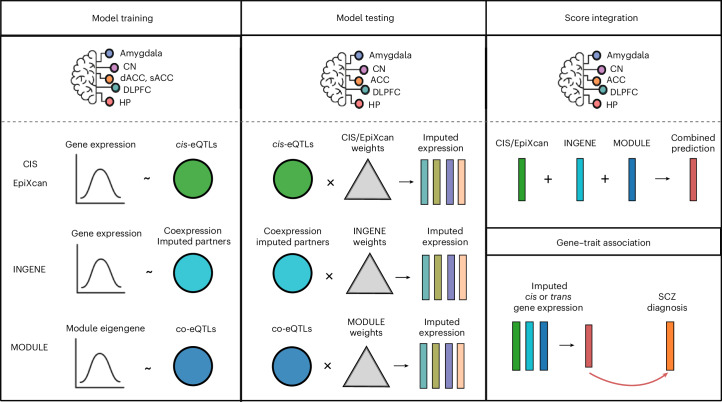
Analytical framework for *cis*-informed and *trans*-informed gene expression prediction and trait association. The workflow consists of four stages. (1) Model training: *cis*-based (CIS, EpiXcan) and *trans*-based (INGENE, MODULE) elastic-net models were trained using RNA-seq and genotype data from six LIBD post-mortem brain regions. CIS and EpiXcan model local (±1 Mb) eQTL effects, whereas INGENE leverages *cis*-regulated coexpression partners and MODULE captures SNPs associated with module eigengenes (co-eQTLs). (2) Model testing: trained weights were applied to independent genotype datasets (GTEx and CMC) to impute gene-level expression and evaluate cross-cohort reproducibility. (3) Score integration: *cis*-based and *trans*-based predictions were combined to generate unified gene-level expression estimates. (4) Gene–trait association: integrated genetically imputed expression was tested for association with SCZ diagnosis across 62 PGC3 cohorts through coTWAS. CN, caudate nucleus; ACC, anterior cingulate cortex; HP, hippocampus.

We evaluated whether INGENE and MODULE increase the number of genetically predictable risk genes beyond *cis*-based models and whether integrating *cis* and *trans* components enhances predictive accuracy. We then applied our framework in a coexpression-informed TWAS (coTWAS) of SCZ using 62 Psychiatric Genomic Consortium (PGC) wave 3 (ref. ^5^) cohorts. The overall study design is summarized in Fig. 1.

## Results

### INGENE and MODULE increase the number of predicted genes across brain regions

#### Evaluation of *cis* and *trans* model training performance

**Fig. 2:**
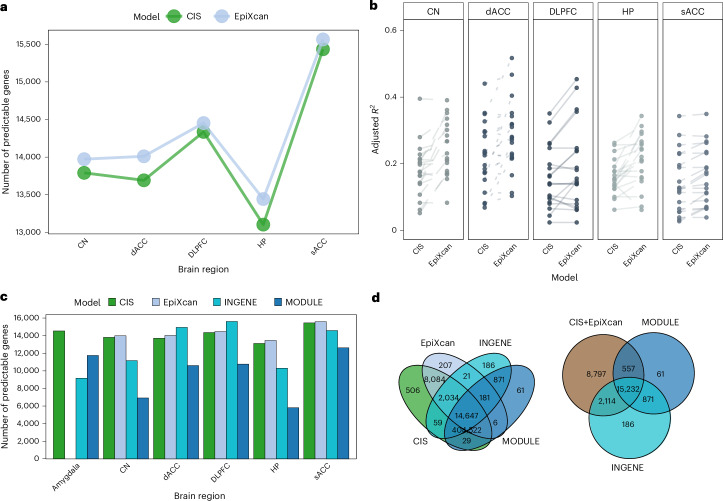
Comparative performance of *cis* and *trans* prediction models across brain regions. **a**, Number of predictable genes for CIS and EpiXcan across LIBD brain regions. **b**, Gene-level comparison of cross-validated adjusted *R*^2^ between CIS and EpiXcan for the top 20 genes per brain region ranked by improvement in cross-validated adjusted *R*^2^. Each point represents one gene (*n* = 20 per region), and paired points are connected by lines. **c**, Number of predictable genes across brain regions for all models: CIS, EpiXcan, INGENE and MODULE. *Trans*-aware models substantially expand the number of genes whose expression can be genetically predicted. **d**, Overlap of predicted genes across models. Left, intersection among CIS, EpiXcan, INGENE and MODULE. Right, overlap of *trans*-aware models with the union of *cis* predictors (CIS + EpiXcan).

We trained models using the Lieber Institute for Brain Development (LIBD) brain RNA sequencing (RNA-seq) dataset, which provides the largest available sample sizes across the examined datasets (Table 1). As benchmarks, we implemented a standard elastic-net model^11^ (CIS) and EpiXcan^12^, which incorporates Roadmap Epigenomics^50^ chromatin priors (except for the amygdala). Across five regions, EpiXcan increased both the number of predictable genes and training accuracy relative to CIS (mean adjusted *R*^2^ of 0.153 vs 0.140; ~9% relative gain; Fig. 2a and Supplementary Fig. 1). However, the models showed partial complementarity: approximately 6–8% of genes were uniquely captured by either approach, or CIS outperformed EpiXcan for a subset of genes in each region (caudate nucleus, 1,558; dorsal anterior cingulate cortex (dACC), 1,569; dorsolateral prefrontal cortex (DLPFC), 1,562; hippocampus, 1,736; subgenual anterior cingulate cortex (sACC), 1,193; Fig. 2b). Both *cis* models were therefore retained in downstream analyses.

**Table 1:** Sample characteristics of brain transcriptomic datasets used for model training and validation

Brain region		LIBD				GTEx				CMC						
		*n*	Age (mean ± s.d.)	Sex (% female)	Ancestry	*n*	Age (mean ± s.d.)	Sex (% female)	Ancestry	*n*	Age (mean ± s.d.)			Sex (% female)		Ancestry
ACC	dACC	176	47 ± 15	35.3	EUR	141	60 ± 10	28.4	EUR	159	61 ± 18			33%		EUR
	sACC	508	47 ± 16	31	EUR											
Amygdala		461	47 ± 16	29	EUR	114	58 ± 10	30%	EUR							
CN		211	50 ± 16.4	25.1	EUR	199	59 ± 10	25%	EUR							
DLPFC		584	46 ± 15	31.5	EUR	160	59.3 ± 9	27%	EUR	387		70 ± 17	40%		EUR	
HP		236	46 ± 16	23	EUR	145	59 ± 11	26.2%	EUR							

The table summarizes sample size and demographic characteristics of post-mortem brain tissue datasets from the LIBD, GTEx and CMC across brain regions. For each dataset and region, the number of samples (*n*), mean age at death (in years), percentage of female donors and ancestry are reported. EUR, European; CN, caudate nucleus; HP, hippocampus.

We next compared the *cis*-based predictors (CIS and EpiXcan) with the *trans*-informed frameworks, INGENE and MODULE. *Trans* models were trained in all available post-mortem datasets, including LIBD, the Genotype-Tissue Expression project (GTEx) and the CommonMind Consortium^37^ (CMC) (Table 1). Only genes replicated across datasets were retained (Supplementary Notes and Supplementary Fig. 2). This cross-dataset filtering reduced false positives and improved stability of *trans*-eQTL signals. *Cis* models were not penalized by this filtering, as we observed that performance was sensitive to training sample size (Supplementary Notes and Supplementary Figs. 3 and 4).

We next quantified training performance across models, revealing heterogeneous predictive capacity across brain regions (Fig. 2c). When pooling regions, CIS predicted 26,285 genes, EpiXcan predicted 25,702, INGENE predicted 18,403 and MODULE predicted 16,721 (Fig. 2d left). *Cis*-based models contributed 8,797 unique predictions (30% of total), whereas INGENE and MODULE showed substantial overlap, sharing 16,103 common predictions (96% of MODULE; 88% of INGENE; Fig. 2d right). Supplementary Fig. 5 shows the unfiltered performance comparison. To examine whether *trans-*predicted genes shared regulatory architecture across frameworks, we assessed whether SNPs acting as *trans*-eQTLs for a MODULE-predicted gene also serve as *cis*-eQTLs for coexpression partners used by INGENE to predict that same target gene. Across brain regions, 15–29% of MODULE-predicted genes exhibited such *cis*–*trans* regulatory relationships (Supplementary Table 1), indicating partial convergence between *cis*-mediated effects and module-level regulatory structure.

Cross-validated adjusted *R*^2^ comparisons for 15,232 commonly predicted genes across models (Fig. 2d right and Supplementary Fig. 6) showed that EpiXcan outperformed CIS (mean delta (Δ)*R*^2^ = 0.0103, one-tailed Wilcoxon signed-rank test, *P* = 2.06 × 10^−20^) and INGENE achieved higher mean adjusted *R*^2^ than CIS (Δ*R*^2^ = 0.0241) and EpiXcan (Δ*R*^2^ = 0.0257), although these differences were not statistically significant. By contrast, MODULE significantly outperformed all other models, with improvements relative to INGENE (Δ*R*^2^ = 0.0464, *P* = 5.04 × 10^−16^), CIS (Δ*R*^2^ = 0.0721, *P* = 3.02 × 10^−3^) and EpiXcan (Δ*R*^2^ = 0.0705, *P* = 1.46 × 10^−4^).

To contextualize our approach, we compared it with published *cis*–*trans* frameworks (Bayesian genome-wide (BGW)-TWAS^51^ and MOSTWAS^52^; Supplementary Notes), restricting analyses to the DLPFC, the only common region. A gene was classified as *cis* or *trans* when the corresponding component contributed to its prediction (Supplementary Fig. 7a). Approximately 59% of BGW *cis*–*trans* predictions and 96% of the *cis*–*trans* set in our framework were recovered by both approaches (Supplementary Fig. 7b), indicating strong concordance in regulatory classification while extending predictions to additional genes.

#### Evaluation of *cis* and *trans* models on a testing dataset

We assessed the replication of all four models—CIS, EpiXcan, INGENE and MODULE—in the independent GTEx cohort across the same brain regions used for model training. We applied each model to GTEx genotype data to generate predictions, thereby avoiding potential overfitting by evaluating performance in an independent testing dataset. We evaluated predictions based on a positive correlation between predicted and observed expression (Pearson’s *r* > 0) and adjusted *R*^2^ > 0.

**Fig. 3:**
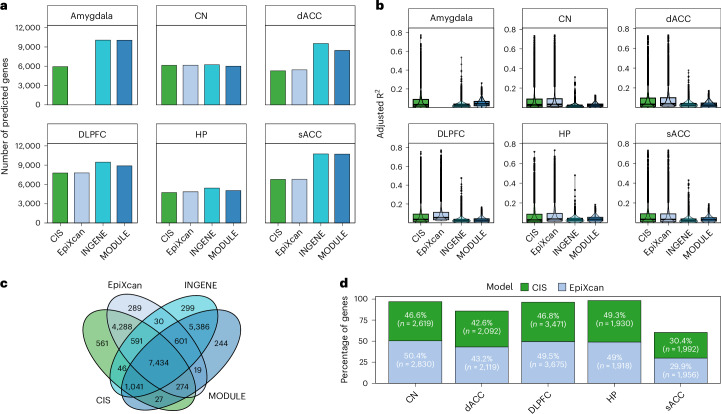
Predictive models replicate across brain regions in the external GTEx dataset and predict sets of genes with different performance. **a**, Barplots showing the number of predicted genes in the GTEx dataset by CIS, EpiXcan, INGENE and MODULE models. **b**, Boxplots of adjusted *R*^2^ values in predicting gene-level expression in GTEx using CIS, EpiXcan, INGENE and MODULE predictive models across brain regions. The median is represented by the central line, with the interquartile range (IQR) as the box. Whiskers extend to 1.5× the IQR, and outliers are plotted as individual points. Each point represents a gene whose expression was predicted in the independent GTEx dataset. The number of genes included per model and region is as follows: amygdala: CIS (*n* = 5,936), INGENE (*n* = 8,052), MODULE (*n* = 10,057); CN: CIS (*n* = 6,140), EpiXcan (*n* = 6,172), INGENE (*n* = 6,228), MODULE (*n* = 6,016); dACC: CIS (*n* = 5,284), EpiXcan (*n* = 5,447), INGENE (*n* = 9,537), MODULE (*n* = 8,455); DLPFC: CIS (*n* = 7,777), EpiXcan (*n* = 7,797), INGENE (*n* = 9,464), MODULE (*n* = 8,895); HP: CIS (*n* = 4,732), EpiXcan (*n* = 4,858), INGENE (*n* = 5,429), MODULE (*n* = 5,048); sACC: CIS (*n* = 6,782), EpiXcan (*n* = 6,799), INGENE (*n* = 10,749), MODULE (*n* = 10,718). **c**, Venn diagram showing the number of total intersecting predicted genes among models. **d**, Barplots showing the percentage corresponding to ‘*n*’ genes predicted within each region for which CIS or EpiXcan achieves the higher adjusted *R*^2^ among genes predicted by both models.

In GTEx, CIS predicted between 4,732 and 7,777 genes across regions. EpiXcan predicted 4,858–7,797 genes. INGENE achieved broader coverage, predicting 5,429–10,749 genes, while MODULE predicted 5,084–10,718 genes (Fig. 3a). Therefore, both *trans* models substantially increased the number of predictable genes relative to *cis*-only approaches, with INGENE predicting up to 1.8-fold more genes than CIS and 1.7-fold more than EpiXcan (Supplementary Fig. 8).

As expected, *cis*-based models explained a higher proportion of expression variance per gene than *trans*-informed models. In GTEx, mean adjusted *R*^2^ ranged from 7.1% to 7.8% for CIS and from 7.2% to 9.4% for EpiXcan, aligning with previous findings^12^. By contrast, INGENE explained between 2% and 3.3% of variance, and MODULE explained between 2.7% and 4.9% (Fig. 3b and Supplementary Fig. 9). Across regions, EpiXcan significantly exceeded INGENE and MODULE in mean adjusted *R*^2^ (one-tailed Wilcoxon test, all *P* < 2.2 × 10^−16^; Supplementary Fig. 10). Compared to CIS, EpiXcan showed significantly higher performance in caudate nucleus, dACC, DLPFC and hippocampus (all one-tailed Wilcoxon tests, *P* < 2.2 × 10^−16^), whereas no significant difference was observed in sACC (*P* = 0.64). Notably, testing performance differed from training estimates, highlighting the importance of external validation.

Despite the substantial overlap across frameworks (Venn diagram in Fig. 3c), each method contributed unique predictions in GTEx: CIS identified 561 genes not captured by other models, EpiXcan identified 289, INGENE identified 299 and MODULE identified 244. This complementarity supports retaining all four models within the integrative framework. For genes predicted by both CIS and EpiXcan, we selected the model with the higher adjusted *R*^2^ (Fig. 3d) for downstream analyses, maximizing predictive accuracy while avoiding redundancy.

To further assess reproducibility, we applied all models to the CMC post-mortem datasets (DLPFC and ACC; Table 1) and quantified cross-cohort consistency relative to GTEx (Supplementary Fig. 11). CIS retained 2,136–4,535 common genes (32–47% overlap) with cross-cohort correlations of *r* = 0.43–0.68, and EpiXcan showed similar replicability (2,183–4,565 genes, 33–47%; *r* = 0.43–0.68). INGENE yielded 11,057–12,274 shared genes (77–79%) with *r* = 0.17–0.52, whereas MODULE retained 17,594–19,385 genes (~88%) with *r* = 0.14–0.17 (Supplementary Fig. 11). Although *cis* predictors exhibited higher correlation, *trans*-informed models demonstrated a higher percentage of shared genes across independent cohorts.

We further benchmarked our LIBD-trained models against BGW^51^ and MOSTWAS^52^ in DLPFC across both CMC and GTEx datasets (Supplementary Notes). Across cohorts, our models predicted the largest number of genes and showed comparable or higher adjusted *R*^2^ distributions (Supplementary Fig. 7c,d). The broader performance range observed for BGW-TWAS and MOSTWAS probably reflects their integration of *cis* and *trans* components, whereas INGENE and MODULE, which rely exclusively on *trans* signals, showed narrower and more stable distributions. Notably, the median predictive accuracy of INGENE and MODULE exceeded that of the other *trans*-informed models.

Taken together, these results show that our coexpression-based approach improves gene coverage while maintaining replicability across independent datasets.

### Functional genetics of co-eQTLs

To define the regulatory architecture of *trans*-associated variants, we examined MODULE-derived *trans*-SNPs that were also significant *cis*-eQTLs in the GTEx multi-tissue QTL resource^53^ (Supplementary Table 3). Depending on the region, 25–44% of *trans*-SNPs overlapped with GTEx *cis*-eQTLs and were associated with 5,821–19,276 *cis-*regulated eGenes (Supplementary Table 3). These results show that a substantial portion of the *trans* signals detected by MODULE arises from SNPs that also exert *cis* effects on nearby genes. Gene Ontology enrichment of these *cis*-eGenes revealed overrepresentation of ATP-dependent and catalytic activities, electron-transferase functions and protein-binding categories, including major histocompatibility complex (MHC) class II binding and cadherin binding (false discovery rate (FDR) < 0.05; Supplementary Fig. 12).

Regulomic analysis identified 252 transcription factors significantly enriched among GTEx *cis*-eGenes linked to MODULE *trans*-SNPs (Bonferroni *P* < 0.05; Supplementary Fig. 13a). Enriched transcription factors included *GATAD2A*, *RERE*, *IRF3* and *SP4*, genes previously prioritized in SCZ GWAS^5^ (Supplementary Fig. 13b).

Together, these findings suggest that a subset of *trans*-eQTLs reflects coordinated regulatory programs involving transcriptional control mechanisms.

#### Functional enrichment of SCZ risk variant-associated genes in predictive models

We next assessed whether regulatory SNP weights were enriched among SCZ-associated variants^5^ with model-derived SNP weights (defined as the mean absolute weight across all regulated genes). We did not perform the same analysis for INGENE, as its predictions derive from *cis*-regulatory SNPs of coexpressed partner genes rather than direct SNP-to-gene associations, and therefore reflect propagated partner-gene *cis* effects rather than regulation of the target gene itself. Across brain regions, both *cis*-based and *trans-*based models showed significant positive correlations between QTL weights and the effect sizes of SCZ-associated variants (Supplementary Table 4 and Supplementary Fig. 14), indicating that SNPs with stronger regulatory effects are associated with higher disease risk. CIS and EpiXcan showed comparable correlations across regions (*r* = 0.14–0.18), whereas MODULE consistently achieved markedly stronger correlations (*r* = 0.28–0.42) (Supplementary Table 4). To assess whether these differences were significant, correlations were compared using Fisher’s *z*-test, confirming that MODULE outperformed CIS and EpiXcan across regions (*P* < 2 × 10^−16^; Supplementary Fig. 15). These results suggest a stronger association of *trans*-eQTLs than for *cis*-eQTLs with common variant pathogenicity. Similar trends were observed for bipolar and major depressive disorders (Supplementary Fig. 14).

We further tested whether *trans*-predicted genes that are enriched for SCZ-associated SNPs, quantified by the PGC weight ratio (Supplementary Notes), showed increased connectivity^27^ to PGC3-prioritized genes^5^ across regions. When genes were ranked by PGC weight quintiles, mean connectivity increased monotonically, as confirmed by linear regression, Spearman’s correlation and permutation testing (*n* = 1,000 permutations per model-region pair; Supplementary Fig. 16, Supplementary Table 5 and Supplementary Notes). MODULE showed robust enrichment across DLPFC, caudate nucleus, hippocampus and sACC (all *P* < 0.001), whereas CIS exhibited a weaker effect in the amygdala (*P* = 0.037) and EpiXcan showed no significant enrichment. These results indicate that *trans*-derived signals preferentially localize to coordinated coexpression networks linked to SCZ risk^27^.

### Combination of *cis* and *trans* scores enhances gene expression prediction in GTEx

**Fig. 4:**
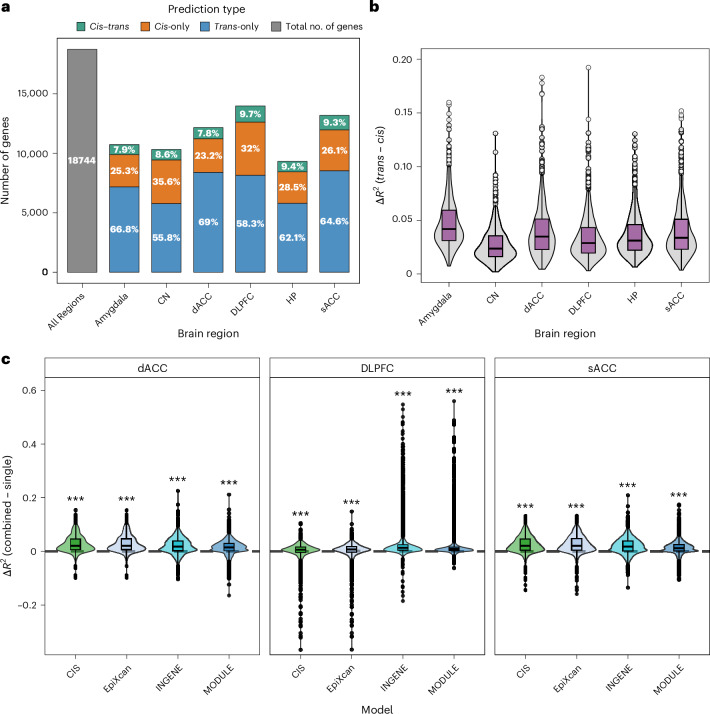
INGENE and MODULE *trans* scores improve gene prediction across brain regions. **a**, Number of genes significantly predicted by *cis*-only, *trans*-only and *cis*–*trans* models across regions; gray indicates unique genes when pooling regions. Best-fitting model counts per region: amygdala (*trans*, 7,169; *cis*, 2,717; *cis*–*trans*, 847), CN (*trans*, 5,763; *cis*, 3,674; *cis*–*trans*, 889), dACC (*trans*, 8,393; *cis*, 2,822; *cis*–*trans*, 949), DLPFC (*trans*, 8,148; *cis*, 4,475; *cis*–*trans*, 1,362), HP (*trans*, 5,790; *cis*, 2,654; *cis*–*trans*, 881), sACC (*trans*, 8,521; *cis*, 3,443; *cis*–*trans*, 1,232). **b**, Distribution of Δ*R*^2^ = adjusted *R*^2^ (*cis* + *trans*) − adjusted *R*^2^ (*cis*) for genes for which *cis*–*trans* was the best model. Violin plots show kernel density. Boxplots show the median (center line), IQR (box bounds, 25th–75th percentiles), whiskers extending to the most extreme values within 1.5× the IQR and points indicating outliers beyond the whiskers. The dashed horizontal line indicates Δ*R*^2^ = 0. Median Δ*R*^2^: amygdala, 0.0418 (*n* = 847); CN, 0.0238 (*n* = 889); DLPFC, 0.0288 (*n* = 1,362); HP, 0.0311 (*n* = 881); dACC, 0.0348 (*n* = 949); sACC, 0.0337 (*n* = 1,232). **c**, Distribution of Δ*R*^2^ = adjusted *R*^2^ (combined) − adjusted *R* ^2^ (single model) in the independent CMC dataset, stratified by brain region and model (CIS, EpiXcan, INGENE and MODULE). Violin and boxplots are defined as in **b**. Each point represents one gene. Statistical significance was assessed using one-sided Wilcoxon signed-rank tests (*H*_1_: combined > individual). Exact *P* values and sample sizes were: DLPFC: CIS, *n* = 3,875, *P* = 6.30 × 10^−80^; EpiXcan, *n* = 3,935, *P* = 5.23 × 10^−79^; INGENE, *n* = 8,543, *P* < 2.2 × 10^−308^; MODULE, *n* = 7,026, *P* < 2.2 × 10^−308^. dACC: CIS, *n* = 1,602, *P* = 9.66 × 10^−201^; EpiXcan, *n* = 1,633, *P* = 1.03 × 10^−207^; INGENE, *n* = 7,575, *P* < 2.2 × 10^−308^; MODULE, *n* = 6,663, *P* < 2.2 × 10^−308^. sACC: CIS, *n* = 2,108, *P* = 4.44 × 10^−234^; EpiXcan, *n* = 2,108, *P* = 2.71 × 10^−236^; INGENE, *n* = 8,222, *P* < 2.2 × 10^−308^; MODULE, *n* = 7,608, *P* < 2.2 × 10^−308^.

We next tested whether combining *cis* and *trans* predictions improves gene expression imputation in GTEx. For genes with both *cis* and *trans* components, we compared two nested models: a baseline model including covariates and *cis-*derived predictions; and a full model including covariates, *cis*-predicted expression and *trans*-predicted expression. Model comparison was performed using maximum likelihood estimation (α = 0.05). Genes were considered enhanced by *trans* predictions when the inclusion of the *trans* component led to a significant increase in adjusted *R*^2^ relative to the *cis*-only model. Across brain regions, integration of *cis* and *trans* predictors increased the number of significantly predictable genes (Fig. 4a). For these genes, inclusion of the *trans* term in the maximum likelihood estimation model consistently yielded a significant increase in variance explained compared to the *cis-*only model. This suggests that the *trans* component provides additional explanatory power that local genetic regulation alone does not capture (Fig. 4b). When pooling regions, 18,744 genes were significantly predicted by the integration of all models (Fig. 4a).

Having shown that *trans* components improve prediction for a subset of genes, we next constructed a unified model combining *cis*-derived and *trans*-derived predictions. Given that *cis*-SNPs were excluded when generating *trans* predictors, these components were independent. For genes with at least two predictors, we fit a linear model in GTEx using observed expression as the outcome and CIS, EpiXcan, INGENE and MODULE predictions as explanatory variables. We evaluated model performance in the CMC (dACC, DLPFC and sACC).

Combining *cis*-based and *trans*-based predictions into a single model (‘combined’ in Fig. 4c) improved gene expression prediction across all regions, explaining 4.1–5.0% of variance across 7,640–8,600 genes. By comparison, CIS and EpiXcan accounted for 2.3–6.4% of variance in 1,602–3,935 genes, while INGENE and MODULE explained 1.8–2.9% and 1.4–3.7% across 6,663–8,543 genes, respectively. Across regions, the combined model yielded significantly higher adjusted *R*^2^ values than any individual predictor (one-sided Wilcoxon signed-rank test; Fig. 4c). We therefore used the combined model for subsequent gene–trait association analyses.

### coTWAS identifies trait associations in PGC3 cohorts

To identify gene associations with SCZ, we applied the integrated *cis*–*trans* framework to 62 PGC3 (ref. ^5^) cohorts (102,613 individuals; Supplementary Table 6). Within each cohort and brain region, we retained genes that met GTEx selection criteria (Supplementary Fig. 17).

We associated predicted expression with SCZ diagnosis using logistic regression followed by meta-analysis of effect sizes across cohorts (coTWAS). To identify independent signals, we performed conditional analysis^13^ across 315 genomic regions (±1 Mb window) with FDR-corrected results (FDR = 0.01) to account for overlapping subjects across brain regions.

**Fig. 5:**
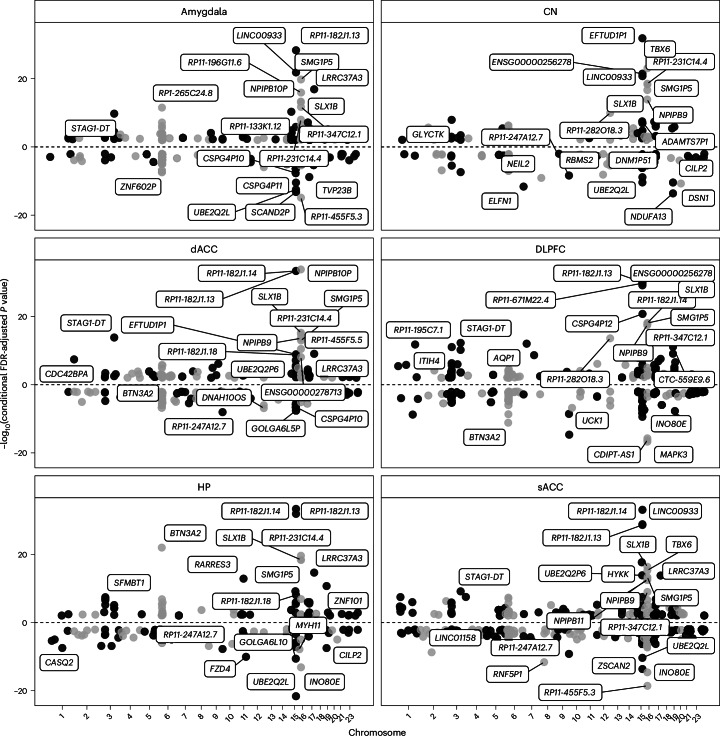
Gene-level conditional association results (coTWAS) across brain regions. Chromosomal position and −log_10_ conditional *P* values after FDR correction (FDR < 0.01). Conditional association statistics were derived using the coTWAS framework. Each point represents one gene identified as significant in the conditional analysis (amygdala, *n* = 157; CN, *n* = 151; dACC, *n* = 182; DLPFC, *n* = 232; HP, *n* = 157; sACC, *n* = 283; total, *n* = 766). The dashed horizontal line indicates the significance threshold. Genes above the dashed line represent positive effects (upregulated in cases), and those below the line represent negative effects (downregulated in cases). For clarity, a maximum of 50 gene names per region are shown.

Across 96,535 tests, we identified 1,162 significant associations (975 non-MHC), corresponding to 766 independent genes (693 non-MHC; Table 2 and Supplementary Table 7). Of these, 381 genes were genetically upregulated and 414 were downregulated in SCZ (Fig. 5).

**Table 2:** Summary of significant coTWAS gene-level associations across brain regions

Brain region	Total number of tests	Number of significant genes	β direction (positive/negative)	Total number of unique genes
Amygdala	96,535	157	78/79	766
CN		151	73/78	
dACC		182	98/84	
DLPFC		232	130/102	
HP		157	71/86	
sACC		283	120/163	

The table reports the number of genes significantly associated with SCZ in each brain region following conditional coTWAS analysis. Total number of tests indicates the total number of gene-level conditional association tests performed across all regions. Number of significant genes denotes the number of genes reaching significance in each region after FDR correction (FDR < 0.01). β direction indicates the number of genes with positive or negative effect sizes (β), corresponding to higher or lower genetically predicted gene expression associated with cases, respectively. Total number of unique genes indicates the total number of distinct genes identified across all regions combined. The unit of analysis is the gene.

Leave-site-out validation across cohorts identified 556 replicable genes (empirical one-tailed *P* < 0.05; Supplementary Fig. 18 and Supplementary Table 7) with a mean (±s.d.) concordance rate of 69.4% ± 5.1% and mean cross-site effect-size correlation *R*^2^ = 0.34 ± 0.11. These results indicate that about 70% of site-specific effect directions were consistent across leave-out iterations, supporting consistency across cohorts.

**Fig. 6:**
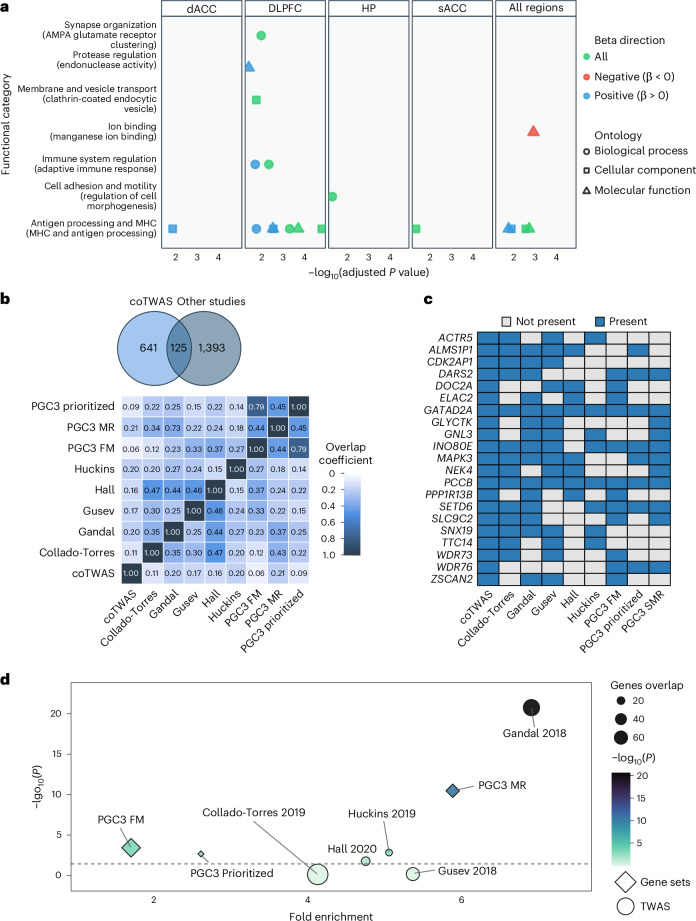
Integrative analysis of coTWAS-identified genes and SCZ-associated gene sets. **a**, Gene Ontology enrichment analysis of coTWAS-significant genes across brain regions. Enrichment significance was assessed using a one-sided hypergeometric test, with FDR correction applied for multiple comparisons. The *x* axis shows a −log_10_ scale of adjusted FDR *P* values for the top enriched term, and the *y* axis shows functional categories grouped by Gene Ontology domains. Marker colors indicate the direction of the genetic effect; marker shapes denote ontology categories. **b**, Overlap between coTWAS-identified genes and previously reported SCZ-associated gene sets. The Venn diagram (top) shows the intersection between coTWAS genes and genes reported in previous studies, including Huckins et al.^13^, SCHEMA^61^, PGC3 Mendelian randomization (MR), PGC3 fine-mapping (FM) and PGC3-prioritized genes^5^, Borcuk et al.^27^, Gandal et al.^58^, Gusev et al.^62^, Hall et al.^59^ and Collado-Torres et al.^60^. The matrix (bottom) shows pairwise overlaps quantified by the Szymkiewicz–Simpson overlap coefficient, in which darker blue colors indicate larger proportional overlap values. **c**, Presence–absence heatmap of genes present in at least four studies (*n* ≥ 4) across the indicated datasets. Rows represent genes and columns represent studies. Blue squares indicate that the gene was reported in the corresponding study, whereas white squares indicate its absence. **d**, Fold-enrichment versus −log_10_(*P*) for the overlap between coTWAS genes (*n* = 766) and each SCZ-associated gene set. Point size indicates the number of overlapping genes. Marker color represents −log_10_(*P* values), with darker colors indicating stronger statistical significance. Marker shapes distinguish data types: diamonds represent gene sets and circles represent TWAS-derived gene lists. The dashed horizontal line indicates the nominal significance threshold (*P* = 0.05). Enrichment was assessed using a one-sided hypergeometric test for overrepresentation.

Gene Ontology enrichment revealed regionally structured biology (Fig. 6a). In the DLPFC, enrichment across β directions converged on synapse organization, particularly AMPA receptor clustering, and membrane or vesicle transport pathways, including clathrin-coated vesicles, late endosome and lysosomal membrane, indicating altered receptor trafficking. Antigen-processing and MHC pathways were significantly enriched and upregulated (β > 0) in DLPFC and dACC and remained significantly enriched across regions when all β directions were considered, supporting their widespread role in individuals with SCZ. These findings delineate regionally stratified biology, with excitatory synaptic remodeling predominating in prefrontal cortex and immune-related processes distributed across cortical and limbic regions.

Among coTWAS significant genes, 354 were predicted by a single model; CIS accounted for 21%, EpiXcan accounted for 20%, INGENE accounted for 40% and MODULE accounted for 19%. Across regions, *cis* components represented 26.5–54.3% of associations, *cis*–*trans* represented 17.2–30% and *trans*-only represented 18.5–44.5% (Supplementary Fig. 19).

MAGMA *z*-score (Supplementary Notes) correlated with *cis*-predicted genes (*r* = 0.10, *P* = 0.003) but not with *trans*-only predictions (Supplementary Fig. 20; *r* = −0.02, *P* = 0.78), consistent with MAGMA’s underlying design, which focuses on SNPs located proximal to genes. Complementarity was further illustrated by genes showing strong coTWAS evidence (log_10_(adjusted *P*) > 4) but weak MAGMA support (|*z*| < 4; Supplementary Fig. 21).

Cell specificity enrichment using a human atlas^54^ revealed region-specific patterns (Supplementary Fig. 22) of transcriptional perturbation. In the amygdala, upregulated genes (β > 0) were overrepresented in excitatory DG (exDG)-like neurons. In dACC, neuronal stem cells showed enrichment for upregulated genes, while exDG-like neurons were enriched among downregulated genes (β < 0). In DLPFC, pyramidal neurons and exDG-like neurons exhibited upregulation, whereas GABAergic interneurons (GABA2) showed downregulation. These results suggest region-specific and cell-type-specific transcriptional alteration, consistent with neuronal dysfunction in SCZ.

coTWAS identified several previously reported SCZ-related genes, including *SNX19* (ref. ^55^), *CYP21A1P*^56^ and *C4A*^57^ (downregulated across all regions), and *GATAD2A* (upregulated in DLPFC). These genes were predominantly driven by *cis* components, with additional region-specific contributions from *trans* models.

Comparison with prior studies^5,13,27,58–62^ showed that 125 genes had been previously reported (Fig. 6b), whereas 641 (83.7%) were novel (Supplementary Fig. 23a). Known associations were largely recovered by *cis* and *cis*–*trans* models (28.8–56%), while novel associations were enriched among *trans*-only predictions (51%; Supplementary Fig. 23b), indicating that distal regulation drives the new findings in our framework.

Overlap was observed with SCHEMA^61^, PGC3-prioritized, Mendelian randomization and fine-mapped gene sets^5^ (Supplementary Figs. 24–27), and 21 genes were consistently detected in at least four independent studies (Fig. 6c). Although this overlapping set was not enriched for specific gene ontology, key risk loci emerged, including *MAPK3* and *INO80E*, both residing within the 16p11.2 risk locus^63^, as well as *GATAD2A*, a component of the NuRD chromatin-remodeling complex, and *GNL3*, a regulator of neural progenitor maintenance^64^. Enrichment was strongest for the Gandal gene set^58^ (*P* = 2.98 × 10^−21^) and, to a lesser extent, with the Huckins^13^ (*P* = 0.002) and Hall^59^ (*P* = 0.023) (Fig. 6d).

Mapping coTWAS-significant genes to PGC3 loci^5^ showed that 158 genes (27.4% of all tissue-specific associations) fell within significant genome-wide regions, while 608 (72.6%) lay outside (Supplementary Fig. 28a). *Cis* and *cis*–*trans* signals predominated within loci, while *trans*-only associations were largely distal (Supplementary Fig. 28a; average of 2.0% within loci vs 28.2% outside). Several of the strongest associations (−log_10_(FDR) > 20) clustered outside established GWAS peaks (Supplementary Fig. 28c), highlighting regulatory effects in addition to known SCZ loci.

Together, our coTWAS identified 766 SCZ-associated genes, 641 of which represent novel transcriptome-wide associations, demonstrating that incorporating coexpression-informed *trans* regulation expands gene discovery beyond *cis*-only TWAS frameworks.

## Discussion

In this study, we developed coexpression-informed *trans*-eQTL prediction frameworks across six brain regions and integrated them with established *cis*-based models to improve genetically imputed gene expression. By explicitly modeling distal regulatory effects, we expanded the pool of predictable genes and demonstrated that combining *cis* and *trans* components enhances gene-level prediction accuracy. Applying the framework to 102,613 individuals from PGC3, we found 766 significant SCZ-associated genes, 641 of which represent novel transcriptome-wide associations.

Our cross-dataset training and validation strategy prioritized reproducible *trans* signals while preserving the strengths of established *cis-*based predictors. As expected, *cis* models explained larger per-gene variance than *trans* models within the testing dataset (Fig. 3b and Supplementary Fig. 10), reflecting the larger effect size of local eQTLs. However, *trans*-informed models substantially increased the number of genes for which genetically regulated expression could be robustly imputed (Fig. 3c) across independent datasets (GTEx and CMC; Supplementary Fig. 11). Integration of *cis* and *trans* scores significantly enhanced gene-level accuracy relative to single-modality models (Fig. 4a,b), indicating that distal regulatory effects provide complementary information in addition to genetic control. This complementarity aligns with the polygenic and network-based architecture of SCZ, in which multiple interacting genes contribute to multiple biological pathways to influence disease risk^28,29,39,65^.

INGENE and MODULE highlight distinct but complementary features of distal regulation. INGENE models *cis*-mediated *trans* effects^66^ through coexpression partners, whereas MODULE captures shared variance across coexpressed genes^28,38^. Notably, the two approaches converge on a substantial set of predicted genes, suggesting partially shared regulatory mechanisms (Supplementary Table 1). Consistent with this overlap, up to 29% of MODULE-predictable genes have at least one *cis*-SNP regulating a coexpression partner used by INGENE, indicating that part of the MODULE signal may reflect *cis*-mediated effects propagated through coexpression networks. We found that approximately 40% of MODULE *trans*-eQTLs also function as *cis*-eQTLs in independent tissues, based on data from the GTEx QTL resource (Supplementary Table 3), consistent with the idea that regulatory variants can exert both local and distal effects. Enrichment of transcriptional regulators among genes linked to *trans* signals (Supplementary Fig. 13a) further supports a potential regulatory role for transcription factors in mediating the effects of *trans*-eQTLs on gene expression^67,68^. Several genes have established links with SCZ-relevant pathways^5^, including *GATAD2A*^4^ (gene silencing), *RERE*^61^ (developmental transcriptional repressor), *SP4* (associated with SCZ^69^ and bipolar disorder^70^) and *IRF3* (immune and inflammatory signaling^71^). The gene *ESRRA* regulates a *DRD2* gene coexpression network associated with SCZ risk^40^, while the dysregulation of the HDAC1 protein has been widely reported in the disorder^72,73^.

Notably, most MODULE-predicted gene regulatory mechanisms were not mediated by *cis* effects. The remaining 70% of signals may reflect SNPs acting in *cis* on upstream regulators without direct transcriptional mediation, context-specific *cis*-eQTLs not captured in bulk tissue (for example, brain region, cell type or developmental stage specificity) or feedback regulation from expressed genes^17,74–76^. One explanation for MODULE predictions is that RNA-seq quantification of individual genes is inherently noisy, and incorporating coexpression with related genes reduces such uncertainty. Therefore, coexpression data enhances the detection of functional eQTLs and thus contributes to reducing false negatives.

Gene-level association methods^11,12^ or isoform-level TWAS^77^ represent a notable advantage by pinpointing genes within loci that fall short of genome-wide significance in GWAS. These studies identified SNPs often exhibiting GWAS *P* values in a range between 5 × 10^−8^ and 10^−3^, suggesting borderline associations that could attain significance in larger studies. Our approach differs from traditional methods by using coexpression-based models, which re-annotate genetic variants to local (*cis*) and distal (*trans*) genes based on statistical association and not gene proximity. Notably, SNP weights derived from our *trans* model MODULE showed stronger positive correlation with PGC3 SCZ GWAS effect sizes than *cis*-derived weights (Supplementary Figs. 14 and 15), indicating that variants contributing to network-level regulation may be preferentially enriched for disease liability.

Gene Ontology enrichment (Fig. 6a) and cell-type specificity analyses (Supplementary Fig. 22) of coTWAS-significant genes highlight two convergent axes of SCZ pathophysiology: dysregulation of AMPA receptor trafficking within excitatory neurons and immune-endosomal activation processes. Consistent with recent large-scale transcriptomic studies^78^, excitatory neurons exhibit pronounced disease-associated expression changes^58^. In the DLPFC, vesicular and endocytic genes enriched in excitatory neurons were linked with AMPAR trafficking and receptor internalization dynamics, in line with prior reports of altered receptor trafficking in SCZ^79,80^. Genes enriched in GABAergic interneurons showed relative downregulation in the same region, supporting models of excitation–inhibition imbalance^81,82^. Across prefrontal and cingulate regions, enrichment of antigen-processing and MHC pathways aligns with evidence that neuroinflammatory activity exacerbates inhibitory neuron downregulation^83^. Although classically immune-related^57^, MHC class I molecules also regulate NMDA and AMPA receptor trafficking and synapse density during development^84^, suggesting that the glia component may not solely reflect neuroimmune processes. Moreover, given that most associations with expression of immune genes, such as with *C4A*, were negative, this suggests alternative mechanisms other than increased immune response. Although certain genes are preferentially expressed in specific cell types, the eQTL effects captured here reflect bulk tissue regulation and may integrate contributions from multiple cellular compartments.

Comparison of coTWAS hits with prior SCZ studies^5,9,13,27,58–61,85^ (Fig. 6b–d and Supplementary Fig. 26) demonstrated both replication and novel findings. Although 125 genes were previously reported, over 80% were newly identified (Fig. 6b and Supplementary Fig. 27a), with *trans*-only models accounting for most novel discoveries (Supplementary Fig. 27b). Significant overlap with Mendelian randomization results^5^ (Fig. 6b bottom) indicates that a subset of associations is supported by large-scale causal inference analyses, although Mendelian randomization relies exclusively on genome-wide significant *cis*-eQTL instruments and therefore does not directly validate *trans*-regulated signals. Several genes replicated across at least four independent SCZ studies (Fig. 6c), converging on pathways involving synaptic signaling, chromatin remodeling and neurodevelopment. Notable examples include *MAPK3* (ref. ^86^), which links glutamatergic transmission to synaptic plasticity programs, and *INO80E*, *GATAD2A* and *GNL3*, previously reported as dysregulated in post-mortem SCZ cortex^63,64^.

Several limitations should be considered. First, predictive model training relied on post-mortem bulk tissue datasets of moderate size, constraining power for *trans*-eQTL detection and training of prediction models^11,87^. Although cross-dataset validation mitigates overfitting, larger training cohorts will probably refine effect estimates. Second, bulk tissue data obscure cell-type-specific effects, and integration with large-scale single-cell eQTL resources may improve resolution^32,88,89^. Third, our study does not account for the influence of sex on gene expression. Sex is known to contribute to the heterogeneity observed in complex diseases like SCZ^90,91^. Fourth, analyses were restricted to European ancestry, as most individuals in PGC3 have this ancestry. Lastly, as with all imputation-based approaches, associations reflect genetically predicted expression and do not establish causality.

Collectively, our findings demonstrate that integrating coexpression as prior information in predictive models and combining *cis* and *trans* components improves gene-level modeling in disorders like SCZ, in which *trans* heritability may contribute substantially to risk. Given that predicted expression is derived independently of GWAS statistics, this framework complements traditional polygenic risk scores while providing gene-level interpretation of genetic liability. The resulting associations provide region-specific directionality of genetic effects, informing potential physiological mechanisms. Overall, our results support a model in which gene–gene interactions within regulatory networks contribute substantially to SCZ risk.

## Methods

### Ethics statement

The research described herein complies with all relevant ethical regulations. This study analyzed previously generated human genomic and transcriptomic datasets from the LIBD, GTEx, CMC and PGC3. All primary data collection and tissue acquisition were conducted with written informed consent and approved by the relevant Institutional Review Boards as described in the original publications and in the Supplementary Notes.

### Subjects

The training dataset comprised genotype and brain tissues from individuals of European ancestry in the LIBD collection, including neurotypical controls, individuals with SCZ, bipolar disorder and major depressive disorder (Table 1).

Replication analyses used GTEx post-mortem data from European ancestry controls aged 20–79 years; CMC post-mortem samples from SCZ, bipolar disorder and neurotypical control European ancestry donors aged 17–90 years (Table 1); and PGC3 genotype data from 102,613 individuals of European ancestry (SCZ = 42%) (Supplementary Table 6).

### Data sources

#### Selection criteria

The discovery dataset included post-mortem brain samples from healthy individuals and individuals diagnosed with SCZ, bipolar disorder or major depressive disorder. To ensure consistency with our primary post-mortem replication dataset (GTEx) and maximize predictive stability, we included only individuals of European ancestry aged ≥17 years.

#### LIBD RNA-seq data

Post-mortem tissue homogenate RNA-seq data were obtained from the LIBD collection across DLPFC^60,92,93^, hippocampus^60,94^, caudate nucleus^95^, amygdala^93,96^, dACC^93^ and sACC^96^ (Supplementary Notes). Genotype–RNA concordance was verified following the SPEAQeasy pipeline (https://research.libd.org/SPEAQeasy-example/swap_speaqeasy.htm).

#### Replication RNA-seq data

GTEx v8 expression data (dbGaP accession no. phs000424.v8.p2) were used for ACC, amygdala, caudate nucleus, DLPFC (Frontal Cortex BA9) and hippocampus. CMC RNA-seq data were obtained from the CMC (DLPFC, syn18097439 v3.0; ACC, syn29442240 v6.0).

#### Genotype data

LIBD genotypes were generated using Illumina BeadChips and imputed through the TOPMed imputation server^97^ using HRC reference panels^98^. GTEx genotypes followed the GTEx pilot analysis^99^ and were imputed with IMPUTE2(ref. ^100^) using the 1000 Genomes Phase 1 freeze as the reference panel^101^. CMC samples were genotyped using the Illumina Infinium HumanOmniExpressExome platform and imputed using the HRC reference panel.

### Data preprocessing

All analyses were performed primarily using R statistical software (v.4.5.0). Additional preprocessing and model preparation steps were performed using Python (v.3.10.19).

#### Genotype post-imputation processing

LIBD, GTEx and CMC genotype datasets were subjected to uniform post-imputation quality control using PLINK (v.1.09). Variants were removed if they were duplicated, deviated from Hardy–Weinberg equilibrium (*P* < 10^−6^) and had a minor allele frequency of <0.01 or missingness of >0.05. Individuals with >2% missing genotypes were excluded. Relatedness was estimated using PLINK --genome, and pairs exceeding third-degree relatedness (identity by descent of >0.125) were removed. Population stratification was evaluated relative to HapMap3 (ref. ^102^) using principal components; individuals with more than 90% European ancestry were excluded.

After quality control, the final SNP counts were 7,521,829 for LIBD, 8,623,182 for GTEx and 5,859,752 for CMC. For downstream analyses, we subset LIBD genotypes to GTEx overlapping SNPs, yielding 6,819,569 SNPs.

#### PGC3 genotype preprocessing

We analyzed 62 European ancestry cohorts from PGC3 (Supplementary Table 6). Genotype acquisition, imputation and calculation of genomic eigenvariates for population stratification were performed by the PGC^5^. Within each cohort, we retained SNPs with INFO > 0.9, missingness of <1%, minor allele frequency of >0.01 and Hardy–Weinberg equilibrium *P* > 10^−6^ (PLINK v.1.9).

### Analysis pipeline

#### Expression quantification and preprocessing

Gene-level mRNA expression was quantified using recount (v.3.22)^103^, with processed counts converted to reads per kilobase per million mapped reads (RPKMs). We selected genes with median RPKM ≥ 0.1 and ≤20% zero values. We log-transformed RPKM in log_2_(RPKM + 1), and we removed outlier subjects (>3 s.d. by inter-array distance^38^). After removing mitochondrial genes, the number of retained genes varied by region (Supplementary Table 8), and we converted LIBD and GTEx expression values to transcripts per million. Expression values were residualized separately within each dataset and brain region using linear models adjusting for relevant technical and biological covariates, including diagnosis (where applicable), sex, age, RNA integrity metrics, mapping rates, post-mortem interval (GTEx/CMC), the first five ancestry components and the first three expression principal components. Neuronal proportion, estimated using BRETIGEA^104^, was strongly correlated with principal components across regions and therefore not included to avoid collinearity. We rank-normalized residuals using RNOmni (v.1.0.1.2)^105^ before downstream analyses. The number of genes retained per region is shown in Supplementary Table 8.

#### *cis*-eQTL discovery

We defined *cis*-eQTLs as SNPs located within ±1 Mb of each gene’s transcription start site, defined as the 5′ gene coordinate (start for genes on the positive strand; end for genes on the negative strand). SNP–gene associations were tested using robust linear models (robustbase v.0.99.4.1 (ref. ^106^)) within a fourfold cross-validated framework. Statistical significance was assessed using *F*-tests (sfsmisc v.1.1.20 (ref. ^107^)), and fold-specific estimates were combined by fixed-effect inverse variance meta-analysis (stats v.3.6.2), yielding a single effect size and *P* value per SNP–gene pair. SNPs were ranked by *P* value, and linkage disequilibrium pruning was performed within ±250 kb of the lead SNP. Independent signals were defined as *R*^2^ < 0.1, with iterative removal of correlated variants^38^.

#### *cis*-eQTL-based models training and application

We trained our CIS model following the PrediXcan^11^ method, using SNPs within ±1 Mb of each gene. Elastic-net regression was implemented with the glmnet^108^ R package (v.4.1.8) and performed in a fourfold cross-validated framework to estimate SNP weights. Models with adjusted *R*^2^ ≥ 0.01 (*P* < 0.05) were retained. Tissue-specific CIS models were applied to test genotypes using MetaXcan^109^ (Predict.py).

To incorporate functional priors, we trained the EpiXcan^12^ model, which integrates epigenomic annotations into an elastic-net penalty. We retrieved annotations from the Roadmap Epigenomics^50^ portal and trained tissue-specific models following the published EpiXcan implementation. When specified, we followed the original parameter settings; otherwise, we aligned procedures with the CIS pipeline for consistency.

### Training and application of coexpression-based predictive models: INGENE and MODULE

#### Coexpression networks

We leveraged 48 previously published WGCNA^110^-derived coexpression networks^29,33,34,36–38,58,76,111,112^. The gray (unassigned) module was excluded from analysis. The genes and module assignments for all networks used in this study are provided in Supplementary Data 1.

#### INGENE

INGENE predicts gene-level expression from genetically inferred expression of *cis*-regulated coexpression partners. For a given gene *g* with *P* partners, the predicted expression *Y*_*g*_ is modeled as a weighted linear combination of partner-level predictions, assuming additive genetic effects:

$${Y}_{g}=\mathop{\sum }\limits_{{p}^{{\prime} }=1}^{{P}^{{\prime} }}{w}_{{p}^{{\prime} },g}$$

where *Y*_*g*_ is the predicted expression of gene *g*, and w_p__′,g_ is the estimated effect size of partner *p*′ on gene *g*.

We first generated *cis*-based predictions (CIS and EpiXcan) in the LIBD training set. These gene-level predictions served as inputs for coexpression partners within the INGENE framework. When both CIS and EpiXcan predicted the same partner, we retained the predictor with the higher adjusted *R*^2^ to maximize partner-level accuracy.

For each brain region and each of the 48 coexpression networks, we trained elastic-net regression models (cv.glmnet^112^ R package) using fourfold cross-validation to estimate partner weights and tune the penalty parameter λ. Models with adjusted *R*^2^ ≥ 0.01 (*P* < 0.05) were retained. Non-zero coefficients were stored to construct the INGENE prediction database.

To apply INGENE to the testing data, we first imputed *cis*-mediated partner expression using CIS and EpiXcan weights, then applied INGENE-derived coefficients to generate gene-level predictions. For genes predicted by multiple networks, we averaged predictions and selected the *cis*-derived estimate with the highest adjusted *R*^2^ between predicted and observed expression for inclusion in the final INGENE output.

#### MODULE

The MODULE framework predicts gene expression using candidate *trans*-eQTLs associated with the coexpression module eigengene. For each network and brain region, we computed the module eigengene as the first principal component (PC1) of the region-specific coexpression matrix. For a gene *g* with a set of *k trans* markers associated with the module eigengene (excluding its *cis*-SNPs within ±1 Mb), the *trans* component of predicted expression is given by:

$${Y}_{g}=\mathop{\sum }\limits_{k-cis}^{k-cis}{w}_{k-{cis},g}{X}_{k-{cis}}$$

where *Y*_*g*_ is the predicted expression of gene *g*, *k*−*cis* is the set of markers removing *cis*-SNPs*, w*_*k*−*cis,g*_ is the SNP effect size and *X*_*k*−*cis*_ is the allele dosage.

To identify SNPs associated with the module eigengene, we first mapped European 1000 Genomes SNPs^113^ within ±100 kb of module genes using MAGMA (v.1.09b)^113^. We then performed fourfold cross-validation (stratified by diagnosis), computing PC1 within each training fold. For each SNP, we tested genotype–PC1 associations using robust linear models (robustbase^106^ R package) and assessed significance with *F*-tests (sfsmisc^107^ R package). SNPs were ranked by *P* value, and cross-fold consistency was summarized using the rank product. Linkage disequilibrium pruning was performed within ±250 kb of the lead SNP (*R*^2^ < 0.1), iteratively removing lower-ranked correlated variants^38^. SNPs within the top 5% of the rank distribution were retained as co-eQTLs.

LIBD genotypes were restricted to selected co-eQTLs, excluding SNPs within ±1 Mb of each target gene to remove residual *cis* effects. Elastic-net models were trained with nested cross-validation to tune λ (Supplementary Notes). Genes with adjusted *R*^2^ ≥ 0.01 (*P* < 0.05) were retained, and non-zero weights were stored using RSQLite (v.2.4.7)^114^. We applied models to test genotypes using MetaXcan^109^. As described in the INGENE section, we used an averaging strategy to consolidate predictions, resulting in a single imputed expression value for each gene.

#### Cross-dataset training of INGENE and MODULE

To enhance robustness and minimize dataset-specific effects, *trans* models were trained independently in LIBD, GTEx and CMC for each available brain region. All trained models were then applied to LIBD genotypes to generate predictions on a common individual-level reference panel.

For each gene *g*, this approach yielded three predicted-expression vectors over the same LIBD individuals:

$${\widehat{{Y}_{g}}}^{{LIBD}\to {LIBD}},{\widehat{{Y}_{g}}}^{{GTEx}\to {LIBD}},{\widehat{{Y}_{g}}}^{{CMC}\to {LIBD}}$$

Replicability was assessed by model-to-model agreement rather than prediction-to-observed expression. For each gene, we computed Pearson correlations between LIBD-trained predictions and those derived from GTEx or CMC:

$${{r}_{g}}^{{GTEx}\to {LIBD},{LIBD}\to {LIBD}}={cor}\left({\widehat{{Y}_{g}}}^{{GTEx}\to {LIBD}},{\widehat{{Y}_{g}}}^{{LIBD}\to {LIBD}}\right)$$

$${{r}_{g}}^{{CMC}\to {LIBD},{LIBD}\to {LIBD}}={cor}\left({\widehat{{Y}_{g}}}^{{CMC}\to {LIBD}},{\widehat{{Y}_{g}}}^{{LIBD}\to {LIBD}}\right)$$

Genes were retained if at least one cross-dataset correlation was positive (*r* > 0). This filtering step was applied to LIBD-trained models and did not incorporate testing datasets in predictive score generation, thereby avoiding information leakage.

#### Functional and regulomic analysis of *trans*-regulated genes

From each tissue-specific MODULE model, we selected *trans*-SNPs predicting genes validated in GTEx and retained *trans*-SNPs with absolute predictive weights of ≥0.01. We focused on SNPs that were also significant *cis*-eQTLs across all 49 GTEx tissues^53^. SNPs were stratified into quartiles based on absolute MODULE weights and linked to their *cis*-eGenes (GTEx catalog; Supplementary Table 3).

Gene Ontology enrichment was performed separately by brain region and for the highest-weight quartile using the ClusterProfiler package^115^ (v.3.22), restricting analyses to molecular function. The universe was defined as all unique GTEx genes.

For regulomic analysis, we used the gProfileR package^116^ (v.0.2.4) to identify transcription factors enriched among GTEx *cis*-eGenes linked to MODULE *trans*-SNPs. The background was defined as genes regulated in *trans* within the MODULE framework. Significance was assessed using hypergeometric testing (stats R package v.3.6.2), with FDR and Bonferroni corrections applied for multiple comparisons.

#### Correlation between predictive SNP weights and PGC3 odds ratios

We obtained GWAS summary statistics for SCZ, bipolar disorder and major depressive disorder from the PGC European ancestry meta-analysis (links in Supplementary Table 9). For SNPs predicting genes validated in GTEx, we computed mean absolute elastic-net weights from CIS, EpiXcan and MODULE models. GWAS summary statistics were restricted to SNPs with *P* < 0.05 and overlapping model-specific SNP sets (Supplementary Table 4).

We assessed the correlation between SNP weights and log(odds ratio) using Pearson correlation (two-tailed). Correlation coefficients were transformed using Fisher’s *z* to stabilize variance. Differences between models were evaluated using:

$${\rm{z}}=\frac{|{z}_{{cis}}-{z}_{{trans}}|}{\sqrt{{s.e.m{.}^{2}}_{{cis}}+{s.e.m{.}^{2}}_{{trans}}}}$$

where standard errors were derived from the number of SNPs contributing to each model. Two-tailed *P* values were obtained from the standard normal distribution.

### Model evaluation

#### ANOVA analysis in GTEx

We assessed whether combining *cis* and *trans* predictions improved explanatory power using maximum likelihood estimation (car R package^117^ v.3.1-5). For each brain region, observed GTEx expression (log transcripts per million) was modeled with covariates including sex, mean age, RNA integrity number, RNA rate, overall mapping rate, post-mortem interval, the first three principal components and the first five genomic eigenvariates.

For genes predicted by a single model (CIS (C), EpiXcan (E), INGENE (I) or MODULE (M)), we compared:

$${H}_{0}:y \sim {covariates}$$

$${H}_{1}:y \sim {y}_{C,E,I,M}+{covariates}$$

For genes with both *cis* and *trans* components, we compared:

$${H}_{0}:y \sim {y}_{{cis}}+{covariates}$$

$${H}_{1}:y \sim {y}_{{cis}}+{y}_{{trans}}+{covariates}$$

For *trans*-only genes:

$${H}_{0}:y \sim {covariates}$$

$${H}_{1}:y \sim {y}_{{trans}}+{covariates}$$

The model (*cis*-only, *cis*–*trans* or *trans*-only) yielding the most significant improvement in fit was selected for each gene (maximum likelihood estimation, α = 0.05).

### coTWAS analysis in PGC3 cohorts

#### Gene prediction and selection

For each PGC site, we imputed gene expression using tissue-specific CIS, EpiXcan, INGENE and MODULE models. When a gene was predicted by multiple networks, we averaged predictions. We retained only genes validated in GTEx (adjusted *R*^2^ and Pearson’s *r* > 0). Genes present in only one model were classified as unimodal; those included in at least two were classified as multimodal.

#### Association testing

Within each PGC cohort, we performed gene-wise logistic regression with diagnosis as the outcome and imputed expression as the predictor, covarying for sex and genomic eigenvariates previously associated with diagnosis^5^. Unimodal genes were tested using model-specific predictions (equation 1). Multimodal genes were tested using the combined *cis*–*trans* predictor trained in GTEx and validated in CMC (equation 2).

1 $${Dx}\left(Y\right)={Unimodal}({C|E|M|I})+{sex}+{GEs}$$

2 $${Dx}\left(Y\right)={Combined}(C+E+M+I)+{sex}+{GEs}$$

To ensure cross-cohort consistency, we focused on gene predictors available in ≥32 PGC3 sites. Site-specific β from logistic regression were meta-analyzed using the Stouffer method (metap v.1.12 (ref. ^118^)), applying weights proportional to the square root of the sample size for each cohort^119^. To assess heterogeneity, we computed Cochran’s *Q*-test and retained genes with a *P* value threshold of >1 × 10^−3^ across PGC3 sites. In total, 96,535 tissue–gene tests were evaluated.

#### Conditional analysis

To identify independent associations and control for correlation among predicted genes arising from linkage disequilibrium between model SNPs, we performed a conditional analysis following GCTA-COJO theory^120^ using CoCo, an R implementation of GCTA-COJO as previously implemented^13^. We defined 315 genomic regions (1 Mb window), including the MHC, based on the 96,535 meta-analyzed tissue–gene pairs.

Within each region, we generated a predicted expression correlation matrix for all tissue–gene pairs by aggregating cohort-specific correlations using root-effective sample size (*n*_eff_, equation (3) weighting, restricted to cohorts with available predictions.

3 $${n}_{\mathrm{eff}}=\frac{4}{\left(\frac{1}{{n}_{\mathrm{cases}}}+\frac{1}{{n}_{\mathrm{controls}}}\right)}$$

Forward stepwise joint regression was applied to all tissue–gene pairs passing collinearity filtering (*r* < 0.9). Genes were iteratively added based on the meta-analytic *P* value until no additional gene met the significance threshold or no joint statistic reached the significance threshold. We corrected the final output list for genome-wide multiple comparisons using the FDR at α = 0.01.

We calculated effect sizes and odds ratios for SCZ-associated genes by adjusting CoCo betas to have unit variance (equation 4):

4 $$\mathrm{Adjusted}\beta ={\beta }_{{CoCo}}\times \sqrt{{GVAR}}$$

where GVAR is the pooled *n*_eff_ weighted variance of the genetically predicted expression of each gene across all cohorts.

#### coTWAS replicability analysis

Given that we did not have access to a PGC-independent dataset, we performed leave-one-site-out validation across the 62 PGC cohorts for gene–tissue pairs passing conditional FDR ≤ 0.01. For each gene, we evaluated directional concordance, defined as the proportion of sites in which the β of the logistic regression fitted on all sites but one has the same direction (same sign) as the β obtained from the model on the held-out site, and the *R*^2^ of the correlation between leave-one-out and held-out β estimates across sites.

Statistical significance was assessed by permuting held-out β values 10,000 times to generate null distributions for both metrics. Replicable genes were defined as those exceeding the 95^th^ percentile (one-tailed α = 0.05) for both directional concordance and *R*^2^.

#### Cell-type specificity analysis

We used cell-type specificity indices derived from single-nucleus RNA-seq of human brains^54^, which distinguish between ten different cell types (neurons and glia). Enrichment was assessed using the mean-rank gene set test implemented in limma (v.3.46)^121^. Multiple comparisons across genes and cell types were controlled using FDR (α = 0.05).

### Reporting summary

Further information on research design is available in the Nature Portfolio Reporting Summary linked to this article.

## Online content

Any methods, additional references, Nature Portfolio reporting summaries, source data, extended data, supplementary information, acknowledgements, peer review information; details of author contributions and competing interests; and statements of data and code availability are available at 10.1038/s41588-026-02646-3.

## Supplementary information

Supplementary Information Supplementary Notes and Supplementary Figs. 1–28 (Supplementary Tables 1–9 provided separately as an Excel file).

Reporting Summary

Peer Review File

Supplementary Table 1 Supplementary Tables 1–9.

Supplementary Data 1 Excel workbook containing the coexpression network data used in this study. The first worksheet lists the reference and source for each network included in the analyses, and subsequent worksheets provide the genes and module assignments for each network.

## Data Availability

All data supporting the conclusions of this study are available within the article and its Supplementary Information. LIBD post-mortem RNA-seq data from the BrainSeq Phase I–III studies for caudate nucleus, DLPFC and hippocampus are available through the Database of Genotypes and Phenotypes (dbGaP) and Globus collections (caudate nucleus, phs003495.v1.p1, https://www.ncbi.nlm.nih.gov/projects/gap/cgi-bin/study.cgi?study_id=phs003495.v1.p1; DLPFC, jhpce#bsp2-dlpfc, http://research.libd.org/globus/jhpce_bsp2-dlpfc/index.html; hippocampus, jhpce#bsp2-hippo, http://research.libd.org/globus/jhpce_bsp2-hippo/index.html). LIBD genotype data are available through dbGaP under accession phs000979.v3.p2 (https://www.ncbi.nlm.nih.gov/projects/gap/cgi-bin/study.cgi?study_id=phs000979.v3.p2). RNA-seq data for amygdala and sACC from the BipSeq study are available through the PsychENCODE Consortium through Synapse (syn5844980) and are managed by the National Institute of Mental Health Repository and Genomics Resource. Raw and processed data for DLPFC, amygdala and dACC samples from the Department of Veterans Affairs Posttraumatic Stress Disorder study are available upon request through the PTSD Brain Bank Resource Request process (https://www.research.va.gov/programs/tissue_banking/ptsd/default.cfm). GTEx post-mortem processed RNA-seq data are publicly available through the GTEx Portal (release v.8). Individual genotype data are available with controlled access through dbGaP under study accession phs000424.v8.p2. CMC MSSM-PENN-Pitt RNA-seq data were obtained upon request through Synapse (DLPFC release 3.0, syn18097439; ACC release 6.0, syn29442240). Corresponding genotype data were accessed at https://www.synapse.org/Synapse:syn18097441. PGC3 individual-level phenotype and genotype data were obtained upon request and are subject to controlled access. Access details can be found at https://pgc.unc.edu/for-researchers/data-access-committee/data-access-information. Researchers must apply for access and comply with the data use agreement. The GWAS summary statistics are publicly available from the PGC for SCZ, bipolar disorder and major depressive disorder through Figshare repositories. GTEx *cis*-eQTLs data for 59 tissues are publicly available at https://gtexportal.org/home/downloads/adult-gtex/qtl. The study also used previously published WGCNA coexpression networks as detailed in the article and in Supplementary Table 9. Coexpression networks are also provided as Supplementary Data 1 to facilitate reproducibility. Gene expression prediction models and analysis outputs generated in this study are available in the Zenodo repository associated with this work (10.5281/zenodo.14959416)^122^. These include prediction models trained in the LIBD dataset (MODULE, INGENE, CIS and EpiXcan) across six brain regions, combined *cis*–*trans* prediction models validated in GTEx and coTWAS association results, including cross-validation outputs.

## Code availability

Custom code developed for the coTWAS analysis pipeline is publicly available at Zenodo (10.5281/zenodo.14959416)^122^. The actively maintained version is hosted on GitHub (https://github.com/fabiana1rossi/coTWAS). This study also used publicly available software, including MetaXcan and PrediXcan (https://github.com/hakyimlab/MetaXcan) and EpiXcan (https://bitbucket.org/roussoslab/epixcan/src/master). A complete list of software packages, tools and resources is provided in Supplementary Table 9.

## References

[1] Sullivan, P. F., Kendler, K. S. & Neale, M. C. Schizophrenia as a complex trait: evidence from a meta-analysis of twin studies. *Arch. Gen. Psychiatry* **60**, 1187–1192 (2003). 14662550 10.1001/archpsyc.60.12.1187

[2] Purcell, S. M. et al. Common polygenic variation contributes to risk of schizophrenia and bipolar disorder. *Nature* **460**, 748–752 (2009). 19571811 10.1038/nature08185 PMC3912837

[3] Allen, N. C. et al. Systematic meta-analyses and field synopsis of genetic association studies in schizophrenia: the SzGene database. *Nat. Genet.* **40**, 827–834 (2008). 18583979 10.1038/ng.171

[4] Ripke, S. et al. Biological insights from 108 schizophrenia-associated genetic loci. *Nature* **511**, 421–427 (2014). 25056061 10.1038/nature13595 PMC4112379

[5] Trubetskoy, V. et al. Mapping genomic loci implicates genes and synaptic biology in schizophrenia. *Nature* **604**, 502–508 (2022). 35396580 10.1038/s41586-022-04434-5 PMC9392466

[6] Mostafavi, H., Spence, J. P., Naqvi, S. & Pritchard, J. K. Systematic differences in discovery of genetic effects on gene expression and complex traits. *Nat. Genet.* **55**, 1866–1875 (2023). 37857933 10.1038/s41588-023-01529-1 PMC12270542

[7] Maurano, M. T. et al. Systematic localization of common disease-associated variation in regulatory DNA. *Science* **337**, 1190–1195 (2012). 22955828 10.1126/science.1222794 PMC3771521

[8] Gandal, M. J., Leppa, V., Won, H., Parikshak, N. N. & Geschwind, D. H. The road to precision psychiatry: translating genetics into disease mechanisms. *Nat. Neurosci.* **19**, 1397–1407 (2016). 27786179 10.1038/nn.4409 PMC9012265

[9] Gusev, A. et al. Integrative approaches for large-scale transcriptome-wide association studies. *Nat. Genet.* **48**, 245–252 (2016). 26854917 10.1038/ng.3506 PMC4767558

[10] Zhu, Z. et al. Integration of summary data from GWAS and eQTL studies predicts complex trait gene targets. *Nat. Genet.* **48**, 481–487 (2016). 27019110 10.1038/ng.3538

[11] Gamazon, E. R. et al. A gene-based association method for mapping traits using reference transcriptome data. *Nat. Genet.* **47**, 1091–1098 (2015). 26258848 10.1038/ng.3367 PMC4552594

[12] Zhang, W. et al. Integrative transcriptome imputation reveals tissue-specific and shared biological mechanisms mediating susceptibility to complex traits. *Nat. Commun.* **10**, 3834 (2019). 31444360 10.1038/s41467-019-11874-7 PMC6707297

[13] Huckins, L. M. et al. Gene expression imputation across multiple brain regions provides insights into schizophrenia risk. *Nat. Genet.* **51**, 659–674 (2019). 30911161 10.1038/s41588-019-0364-4 PMC7034316

[14] Nicolae, D. L. et al. Trait-associated SNPs are more likely to be eQTLs: annotation to enhance discovery from GWAS. *PLoS Genet.* **6**, e1000888 (2010). 20369019 10.1371/journal.pgen.1000888 PMC2848547

[15] Pergola, G. et al. DRD2 co-expression network and a related polygenic index predict imaging, behavioral and clinical phenotypes linked to schizophrenia. *Transl. Psychiatry* **7**, e1006 (2017). 28094815 10.1038/tp.2016.253 PMC5545721

[16] Pergola, G. et al. Combined effect of genetic variants in the GluN2B coding gene (*GRIN2B*) on prefrontal function during working memory performance. *Psychol. Med.* **46**, 1135–1150 (2016). 26690829 10.1017/S0033291715002639

[17] Umans, B. D., Battle, A. & Gilad, Y. Where are the disease-associated eQTLs? *Trends Genet.* **37**, 109–124 (2021). 32912663 10.1016/j.tig.2020.08.009 PMC8162831

[18] Connally, N. J. et al. The missing link between genetic association and regulatory function. *Elife* **11**, e74970 (2022). 36515579 10.7554/eLife.74970 PMC9842386

[19] Chun, S. et al. Limited statistical evidence for shared genetic effects of eQTLs and autoimmune-disease-associated loci in three major immune-cell types. *Nat. Genet.* **49**, 600–605 (2017). 28218759 10.1038/ng.3795 PMC5374036

[20] Yao, D. W., O’Connor, L. J., Price, A. L. & Gusev, A. Quantifying genetic effects on disease mediated by assayed gene expression levels. *Nat. Genet.* **52**, 626–633 (2020). 32424349 10.1038/s41588-020-0625-2 PMC7276299

[21] Loh, P. R. et al. Contrasting genetic architectures of schizophrenia and other complex diseases using fast variance-components analysis. *Nat. Genet.* **47**, 1385–1392 (2015). 26523775 10.1038/ng.3431 PMC4666835

[22] Liu, X., Li, Y. I. & Pritchard, J. K. Trans effects on gene expression can drive omnigenic inheritance. *Cell* **177**, 1022–1034.e6 (2019). 31051098 10.1016/j.cell.2019.04.014 PMC6553491

[23] Liu, X. et al. Functional architectures of local and distal regulation of gene expression in multiple human tissues. *Am. J. Hum. Genet.* **100**, 605–616 (2017). 28343628 10.1016/j.ajhg.2017.03.002 PMC5384099

[24] Battle, A. et al. Characterizing the genetic basis of transcriptome diversity through RNA-sequencing of 922 individuals. *Genome Res.* **24**, 14–24 (2014). 24092820 10.1101/gr.155192.113 PMC3875855

[25] Friston, K., Brown, H. R., Siemerkus, J. & Stephan, K. E. The dysconnection hypothesis (2016). *Schizophr. Res.* **176**, 83–94 (2016). 27450778 10.1016/j.schres.2016.07.014 PMC5147460

[26] Pierce, B. L. et al. Mediation analysis demonstrates that *trans*-eQTLs are often explained by *cis*-mediation: a genome-wide analysis among 1,800 South Asians. *PLoS Genet.* **10**, e1004818 (2014). 25474530 10.1371/journal.pgen.1004818 PMC4256471

[27] Borcuk, C. et al. Network-wide risk convergence in gene co-expression identifies reproducible genetic hubs of schizophrenia risk. *Neuron* **112**, 3551–3566.e6 (2024). 39236717 10.1016/j.neuron.2024.08.005

[28] Pergola, G., Penzel, N., Sportelli, L. & Bertolino, A. Lessons learned from parsing genetic risk for schizophrenia into biological pathways. *Biol. Psychiatry* **94**, 121–130 (2023). 36740470 10.1016/j.biopsych.2022.10.009

[29] Pergola, G. et al. Consensus molecular environment of schizophrenia risk genes in coexpression networks shifting across age and brain regions. *Sci. Adv.* **9**, eade2812 (2023). 37058565 10.1126/sciadv.ade2812 PMC10104472

[30] Panagiotakos, G. & Pasca, S. P. A matter of space and time: emerging roles of disease-associated proteins in neural development. *Neuron* **110**, 195–208 (2022). 34847355 10.1016/j.neuron.2021.10.035 PMC8776599

[31] Cameron, D. et al. Single-nuclei RNA sequencing of 5 regions of the human prenatal brain implicates developing neuron populations in genetic risk for schizophrenia. *Biol. Psychiatry* **93**, 157–166 (2023). 36150908 10.1016/j.biopsych.2022.06.033 PMC10804933

[32] Jaffe, A. E. et al. Profiling gene expression in the human dentate gyrus granule cell layer reveals insights into schizophrenia and its genetic risk. *Nat. Neurosci.* **23**, 510–519 (2020). 32203495 10.1038/s41593-020-0604-z

[33] Hartl, C. L. et al. Coexpression network architecture reveals the brain-wide and multiregional basis of disease susceptibility. *Nat. Neurosci.* **24**, 1313–1323 (2021). 34294919 10.1038/s41593-021-00887-5 PMC10263365

[34] Gandal, M. J. et al. Shared molecular neuropathology across major psychiatric disorders parallels polygenic overlap. *Science* **359**, 693–697 (2018). 29439242 10.1126/science.aad6469 PMC5898828

[35] Ramaswami, G. et al. Integrative genomics identifies a convergent molecular subtype that links epigenomic with transcriptomic differences in autism. *Nat. Commun.* **11**, 4873 (2020). 32978376 10.1038/s41467-020-18526-1 PMC7519165

[36] Radulescu, E. et al. Identification and prioritization of gene sets associated with schizophrenia risk by co-expression network analysis in human brain. *Mol. Psychiatry* **25**, 791–804 (2020). 30478419 10.1038/s41380-018-0304-1

[37] Fromer, M. et al. Gene expression elucidates functional impact of polygenic risk for schizophrenia. *Nat. Neurosci.* **19**, 1442–1453 (2016). 27668389 10.1038/nn.4399 PMC5083142

[38] Pergola, G. et al. Prefrontal coexpression of schizophrenia risk genes is associated with treatment response in patients. *Biol. Psychiatry* **86**, 45–55 (2019). 31126695 10.1016/j.biopsych.2019.03.981

[39] Pergola, G. et al. A miR-137-related biological pathway of risk for schizophrenia is associated with human brain emotion processing. *Biol. Psychiatry Cogn. Neurosci. Neuroimaging* **9**, 356–366 (2024). 38000716 10.1016/j.bpsc.2023.11.001

[40] Torretta, S. et al. NURR1 and ERR1 modulate the expression of genes of a DRD2 coexpression network enriched for schizophrenia risk. *J. Neurosci.* **40**, 932–941 (2020). 31811028 10.1523/JNEUROSCI.0786-19.2019 PMC6975285

[41] Fazio, L. et al. Transcriptomic context of DRD1 is associated with prefrontal activity and behavior during working memory. *Proc. Natl Acad. Sci. USA* **115**, 5582–5587 (2018). 29735686 10.1073/pnas.1717135115 PMC6003490

[42] Kolberg, L., Kerimov, N., Peterson, H. & Alasoo, K. Co-expression analysis reveals interpretable gene modules controlled by *trans*-acting genetic variants. *Elife* **9**, e58705 (2020). 32880574 10.7554/eLife.58705 PMC7470823

[43] Nath, A. P. et al. An interaction map of circulating metabolites, immune gene networks, and their genetic regulation. *Genome Biol.* **18**, 146 (2017). 28764798 10.1186/s13059-017-1279-y PMC5540552

[44] Esmaili, S. et al. Core liver homeostatic co-expression networks are preserved but respond to perturbations in an organism- and disease-specific manner. *Cell Syst.* **12**, 432–445.e7 (2021). 33957084 10.1016/j.cels.2021.04.004

[45] Sportelli, L. et al. Dopamine signaling enriched striatal gene set predicts striatal dopamine synthesis and physiological activity in vivo. *Nat. Commun.* **15**, 3342 (2024). 38688917 10.1038/s41467-024-47456-5 PMC11061310

[46] Selvaggi, P. et al. Genetic variation of a DRD2 co-expression network is associated with changes in prefrontal function after D2 receptors stimulation. *Cereb. Cortex* **29**, 1162–1173 (2019). 29415163 10.1093/cercor/bhy022

[47] Antonucci, L. A. et al. Thalamic connectivity measured with fMRI is associated with a polygenic index predicting thalamo-prefrontal gene co-expression. *Brain Struct. Funct.* **224**, 1331–1344 (2019). 30725232 10.1007/s00429-019-01843-7

[48] Hauberg, M. E., Roussos, P., Grove, J., Børglum, A. D. & Mattheisen, M. Analyzing the role of microRNAs in schizophrenia in the context of common genetic risk variants. *JAMA Psychiatry* **73**, 369–377 (2016). 26963595 10.1001/jamapsychiatry.2015.3018 PMC7005318

[49] Torshizi, A. et al. Deconvolution of transcriptional networks identifies TCF4 as a master regulator in schizophrenia. *Sci. Adv.* **5**, eaau4139 (2019). 31535015 10.1126/sciadv.aau4139 PMC6739105

[50] Kundaje, A. et al. Integrative analysis of 111 reference human epigenomes. *Nature* **518**, 317–330 (2015). 25693563 10.1038/nature14248 PMC4530010

[51] Luningham, J. M. et al. Bayesian genome-wide TWAS method to leverage both *cis*- and *trans*-eQTL information through summary statistics. *Am. J. Hum. Genet.* **107**, 714–726 (2020). 32961112 10.1016/j.ajhg.2020.08.022 PMC7536614

[52] Bhattacharya, A., Li, Y. & Love, M. I. MOSTWAS: multi-omic strategies for transcriptome-wide association studies. *PLoS Genet.* **17**, e1009398 (2021). 33684137 10.1371/journal.pgen.1009398 PMC7971899

[53] GTEx Consortium. The GTEx Consortium atlas of genetic regulatory effects across human tissues. *Science* **369**, 1318–1330 (2020). 32913098 10.1126/science.aaz1776 PMC7737656

[54] Habib, N. et al. Massively parallel single-nucleus RNA-seq with DroNc-seq. *Nat. Methods* **14**, 955–958 (2017). 28846088 10.1038/nmeth.4407 PMC5623139

[55] Ma, L. et al. Schizophrenia risk variants influence multiple classes of transcripts of sorting nexin 19 (*SNX19*). *Mol. Psychiatry* **25**, 831–843 (2020). 30635639 10.1038/s41380-018-0293-0

[56] Cai, L. et al. Implications of newly identified brain eQTL genes and their interactors in schizophrenia. *Mol. Ther. Nucleic Acids* **12**, 433–442 (2018). 30195780 10.1016/j.omtn.2018.05.026 PMC6041437

[57] Sekar, A. et al. Schizophrenia risk from complex variation of complement component 4. *Nature* **530**, 177–183 (2016). 26814963 10.1038/nature16549 PMC4752392

[58] Gandal, M. J. et al. Transcriptome-wide isoform-level dysregulation in ASD, schizophrenia, and bipolar disorder. *Science* **362**, eaat8127 (2018). 30545856 10.1126/science.aat8127 PMC6443102

[59] Hall, L. S. et al. A transcriptome-wide association study implicates specific pre- and post-synaptic abnormalities in schizophrenia. *Hum. Mol. Genet.* **29**, 159–167 (2020). 31691811 10.1093/hmg/ddz253 PMC7416679

[60] Collado-Torres, L. et al. Regional heterogeneity in gene expression, regulation, and coherence in the frontal cortex and hippocampus across development and schizophrenia. *Neuron* **103**, 203–216.e8 (2019). 31174959 10.1016/j.neuron.2019.05.013 PMC7000204

[61] Singh, T. et al. Rare coding variants in ten genes confer substantial risk for schizophrenia. *Nature* **604**, 509–516 (2022). 35396579 10.1038/s41586-022-04556-w PMC9805802

[62] Gusev, A. et al. Transcriptome-wide association study of schizophrenia and chromatin activity yields mechanistic disease insights. *Nat. Genet.* **50**, 538–548 (2018). 29632383 10.1038/s41588-018-0092-1 PMC5942893

[63] Liu, H. et al. Integrated analysis of summary statistics to identify pleiotropic genes and pathways for the comorbidity of schizophrenia and cardiometabolic disease. *Front. Psychiatry* **11**, 256 (2020). 32425817 10.3389/fpsyt.2020.00256 PMC7212438

[64] Yang, Z. et al. The genome-wide risk alleles for psychiatric disorders at 3p21.1 show convergent effects on mRNA expression, cognitive function, and mushroom dendritic spine. *Mol. Psychiatry* **25**, 48–66 (2020). 31723243 10.1038/s41380-019-0592-0

[65] Parikshak, N. N., Gandal, M. J. & Geschwind, D. H. Systems biology and gene networks in neurodevelopmental and neurodegenerative disorders. *Nat. Rev. Genet.* **16**, 441–458 (2015). 26149713 10.1038/nrg3934 PMC4699316

[66] Liu, S. et al. Illuminating links between *cis*-regulators and *trans*-acting variants in the human prefrontal cortex. *Genome Med.* **14**, 133 (2022). 36424644 10.1186/s13073-022-01133-8 PMC9685876

[67] Bryois, J. et al. *Cis* and *trans* effects of human genomic variants on gene expression. *PLoS Genet.* **10**, e1004461 (2014). 25010687 10.1371/journal.pgen.1004461 PMC4091791

[68] Võsa, U. et al. Large-scale *cis*- and *trans*-eQTL analyses identify thousands of genetic loci and polygenic scores that regulate blood gene expression. *Nat. Genet.* **53**, 1300–1310 (2021). 34475573 10.1038/s41588-021-00913-z PMC8432599

[69] Zhou, X. Over-representation of potential SP4 target genes within schizophrenia-risk genes. *Mol. Psychiatry* **27**, 849–854 (2022). 34750502 10.1038/s41380-021-01376-8 PMC9054665

[70] Zhou, X. et al. Transcription factor SP4 is a susceptibility gene for bipolar disorder. *PLoS ONE* **4**, e5196 (2009). 19401786 10.1371/journal.pone.0005196 PMC2674320

[71] Khavari, B. & Cairns, M. J. Epigenomic dysregulation in schizophrenia: in search of disease etiology and biomarkers. *Cells* **9**, 1837 (2020). 32764320 10.3390/cells9081837 PMC7463953

[72] Gavin, D. P. & Sharma, R. P. Histone modifications, DNA methylation, and schizophrenia. *Neurosci. Biobehav. Rev.* **34**, 882–888 (2010). 19879893 10.1016/j.neubiorev.2009.10.010 PMC2848916

[73] Sharma, R. P., Grayson, D. R. & Gavin, D. P. Histone deactylase 1 expression is increased in the prefrontal cortex of schizophrenia subjects: analysis of the National Brain Databank microarray collection. *Schizophr. Res.* **98**, 111–117 (2008). 17961987 10.1016/j.schres.2007.09.020 PMC2254186

[74] Zhang, J. & Zhao, H. eQTL studies: from bulk tissues to single cells. *J. Genet. Genomics* **50**, 925–933 (2023). 37207929 10.1016/j.jgg.2023.05.003 PMC10656365

[75] Ramasamy, A. et al. Genetic variability in the regulation of gene expression in ten regions of the human brain. *Nat. Neurosci.* **17**, 1418–1428 (2014). 25174004 10.1038/nn.3801 PMC4208299

[76] Walker, R. L. et al. Genetic control of expression and splicing in developing human brain informs disease mechanisms. *Cell* **179**, 750–771.e22 (2019). 31626773 10.1016/j.cell.2019.09.021 PMC8963725

[77] Bhattacharya, A. et al. Isoform-level transcriptome-wide association uncovers genetic risk mechanisms for neuropsychiatric disorders in the human brain. *Nat. Genet.* **55**, 2117–2128 (2023). 38036788 10.1038/s41588-023-01560-2 PMC10703692

[78] Skene, N. G. et al. Genetic identification of brain cell types underlying schizophrenia. *Nat. Genet.* **50**, 825–833 (2018). 29785013 10.1038/s41588-018-0129-5 PMC6477180

[79] Carroll, R. C., Beattie, E. C., von Zastrow, M. & Malenka, R. C. Role of AMPA receptor endocytosis in synaptic plasticity. *Nat. Rev. Neurosci.* **2**, 315–324 (2001). 11331915 10.1038/35072500

[80] Cao, Y.-Y. et al. Molecular mechanisms of AMPA receptor trafficking in the nervous system. *Int. J. Mol. Sci.* **25**, 111 (2023). 38203282 10.3390/ijms25010111 PMC10779435

[81] Parellada, E. & Gassó, P. Glutamate and microglia activation as a driver of dendritic apoptosis: a core pathophysiological mechanism to understand schizophrenia. *Transl. Psychiatry* **11**, 271 (2021). 33958577 10.1038/s41398-021-01385-9 PMC8102516

[82] Lányi, O. et al. Excitation/inhibition imbalance in schizophrenia: a meta-analysis of inhibitory and excitatory TMS–EMG paradigms. *Schizophrenia* **10**, 56 (2024). 38879590 10.1038/s41537-024-00476-y PMC11180212

[83] Purves-Tyson, T. D., Brown, A. M., Weissleder, C., Rothmond, D. A. & Shannon Weickert, C. Reductions in midbrain GABAergic and dopamine neuron markers are linked in schizophrenia. *Mol. Brain* **14**, 96 (2021). 34174930 10.1186/s13041-021-00805-7 PMC8235806

[84] Eyford, B. A. et al. Outside-in signaling through the major histocompatibility complex class-I cytoplasmic tail modulates glutamate receptor expression in neurons. *Sci. Rep.* **13**, 13079 (2023). 37567897 10.1038/s41598-023-38663-z PMC10421907

[85] Pardiñas, A. F. et al. Common schizophrenia alleles are enriched in mutation-intolerant genes and in regions under strong background selection. *Nat. Genet.* **50**, 381–389 (2018). 29483656 10.1038/s41588-018-0059-2 PMC5918692

[86] Wang, J. Q., Fibuch, E. E. & Mao, L. Regulation of mitogen-activated protein kinases by glutamate receptors. *J. Neurochem.* **100**, 1–11 (2007). 17018022 10.1111/j.1471-4159.2006.04208.x

[87] Fryett, J. J., Morris, A. P. & Cordell, H. J. Investigation of prediction accuracy and the impact of sample size, ancestry, and tissue in transcriptome-wide association studies. *Genet. Epidemiol.* **44**, 425–441 (2020). 32190932 10.1002/gepi.22290 PMC8641384

[88] Batiuk, M. Y. et al. Identification of region-specific astrocyte subtypes at single cell resolution. *Nat. Commun.* **11**, 1220 (2020). 32139688 10.1038/s41467-019-14198-8 PMC7058027

[89] Ruzicka, W. B. et al. Single-cell multi-cohort dissection of the schizophrenia transcriptome. *Science* **384**, eadg5136 (2024). 38781388 10.1126/science.adg5136 PMC12772489

[90] Hoffman, G. E. et al. Sex differences in the human brain transcriptome of cases with schizophrenia. *Biol. Psychiatry* **91**, 92–101 (2022). 34154796 10.1016/j.biopsych.2021.03.020 PMC8463632

[91] Brown, A. S. The environment and susceptibility to schizophrenia. *Prog. Neurobiol.* **93**, 23–58 (2011). 20955757 10.1016/j.pneurobio.2010.09.003 PMC3521525

[92] Jaffe, A. E. et al. Developmental and genetic regulation of the human cortex transcriptome illuminate schizophrenia pathogenesis. *Nat. Neurosci.* **21**, 1117–1125 (2018). 30050107 10.1038/s41593-018-0197-y PMC6438700

[93] Jaffe, A. E. et al. Decoding shared versus divergent transcriptomic signatures across cortico-amygdala circuitry in PTSD and depressive disorders. *Am. J. Psychiatry* **179**, 673–686 (2022). 35791611 10.1176/appi.ajp.21020162 PMC10697016

[94] Daskalakis, N. P. et al. Systems biology dissection of PTSD and MDD across brain regions, cell types, and blood. *Science* **384**, eadh3707 (2024). 38781393 10.1126/science.adh3707 PMC11203158

[95] Benjamin, K. J. M. et al. Analysis of the caudate nucleus transcriptome in individuals with schizophrenia highlights effects of antipsychotics and new risk genes. *Nat. Neurosci.* **25**, 1559–1568 (2022). 36319771 10.1038/s41593-022-01182-7 PMC10599288

[96] Zandi, P. P. et al. Amygdala and anterior cingulate transcriptomes from individuals with bipolar disorder reveal downregulated neuroimmune and synaptic pathways. *Nat. Neurosci.* **25**, 381–389 (2022). 35260864 10.1038/s41593-022-01024-6 PMC8915427

[97] Taliun, D. et al. Sequencing of 53,831 diverse genomes from the NHLBI TOPMed Program. *Nature* **590**, 290–299 (2021). 33568819 10.1038/s41586-021-03205-y PMC7875770

[98] McCarthy, S. et al. A reference panel of 64,976 haplotypes for genotype imputation. *Nat. Genet.* **48**, 1279–1283 (2016). 27548312 10.1038/ng.3643 PMC5388176

[99] Human genomics. The Genotype-Tissue Expression (GTEx) pilot analysis: multitissue gene regulation in humans. *Science* **348**, 648–660 (2015). 25954001 10.1126/science.1262110 PMC4547484

[100] Howie, B. N., Donnelly, P. & Marchini, J. A flexible and accurate genotype imputation method for the next generation of genome-wide association studies. *PLoS Genet.* **5**, e1000529 (2009). 19543373 10.1371/journal.pgen.1000529 PMC2689936

[101] Auton, A. et al. A global reference for human genetic variation. *Nature* **526**, 68–74 (2015). 26432245 10.1038/nature15393 PMC4750478

[102] Altshuler, D. M. et al. Integrating common and rare genetic variation in diverse human populations. *Nature* **467**, 52–58 (2010). 20811451 10.1038/nature09298 PMC3173859

[103] Collado-Torres, L. et al. Reproducible RNA-seq analysis using recount2. *Nat. Biotechnol.* **35**, 319–321 (2017). 28398307 10.1038/nbt.3838 PMC6742427

[104] McKenzie, A. T. et al. Brain cell type specific gene expression and co-expression network architectures. *Sci. Rep.* **8**, 8868 (2018). 29892006 10.1038/s41598-018-27293-5 PMC5995803

[105] McCaw, Z. R., Lane, J. M., Saxena, R., Redline, S. & Lin, X. Operating characteristics of the rank-based inverse normal transformation for quantitative trait analysis in genome-wide association studies. *Biometrics* **76**, 1262–1272 (2019). 10.1111/biom.13214 PMC8643141 31883270

[106] Todorov, V. & Filzmoser, P. An object-oriented framework for robust multivariate analysis. *J. Stat. Softw.* **32**, 1–47 (2009).

[107] Maechler, M. sfsmisc: Utilities from ‘Seminar fuer Statistik’ ETH Zurich. R package version 1.1-24. *CRAN* https://cran.r-project.org/web/packages/sfsmisc/index.html (2026).

[108] Friedman, J., Hastie, T. & Tibshirani, R. Regularization paths for generalized linear models via coordinate descent. *J. Stat. Softw.* **33**, 1–22 (2010). 20808728 PMC2929880

[109] Barbeira, A. N. et al. Exploring the phenotypic consequences of tissue specific gene expression variation inferred from GWAS summary statistics. *Nat. Commun.* **9**, 1825 (2018). 29739930 10.1038/s41467-018-03621-1 PMC5940825

[110] Langfelder, P. & Horvath, S. WGCNA: an R package for weighted correlation network analysis. *BMC Bioinformatics* **9**, 559 (2008). 19114008 10.1186/1471-2105-9-559 PMC2631488

[111] Werling, D. M. et al. Whole-genome and RNA sequencing reveal variation and transcriptomic coordination in the developing human prefrontal cortex. *Cell Rep.* **31**, 107489 (2020). 32268104 10.1016/j.celrep.2020.03.053 PMC7295160

[112] Li, M. et al. Integrative functional genomic analysis of human brain development and neuropsychiatric risks. *Science* **362**, eaat7615 (2018). 30545854 10.1126/science.aat7615 PMC6413317

[113] de Leeuw, C. A., Mooij, J. M., Heskes, T. & Posthuma, D. MAGMA: generalized gene-set analysis of GWAS data. *PLoS Comput. Biol.* **11**, e1004219 (2015). 25885710 10.1371/journal.pcbi.1004219 PMC4401657

[114] Müller, K., Wickham, H., James, D. A. & Falcon, S. RSQLite: SQLite interface for R. R package version 2.4.7. *CRAN* https://cran.r-project.org/web/packages/RSQLite/index.html (2026).

[115] Yu, G., Wang, L.-G., Han, Y. & He, Q.-Y. clusterProfiler: an R package for comparing biological themes among gene clusters. *OMICS* **16**, 284–287 (2012). 22455463 10.1089/omi.2011.0118 PMC3339379

[116] Kolberg, L., Raudvere, U., Kuzmin, I., Vilo, J. & Peterson, H. gprofiler2—An R package for gene list functional enrichment analysis and namespace conversion toolset g:Profiler. *F1000Res.*10.12688/f1000research.24956.2 (2020). 10.12688/f1000research.24956.1 PMC7859841 33564394

[117] Dietrich, J. P. & Leoncio, W. citation: Software citation tools. R package version 0.12.2. *CRAN* https://cran.r-project.org/web/packages/citation/index.html (2025).

[118] Dewey, M. metap: Meta-analysis of significance values. R package version 1.13. *CRAN* https://cran.r-project.org/web/packages/metap/index.html (2025).

[119] Zaykin, D. V. Optimally weighted Z-test is a powerful method for combining probabilities in meta-analysis. *J. Evol. Biol.* **24**, 1836–1841 (2011). 21605215 10.1111/j.1420-9101.2011.02297.x PMC3135688

[120] Yang, J., Lee, S. H., Goddard, M. E. & Visscher, P. M. GCTA: a tool for genome-wide complex trait analysis. *Am. J. Hum. Genet.* **88**, 76–82 (2011). 21167468 10.1016/j.ajhg.2010.11.011 PMC3014363

[121] Ritchie, M. E. et al. limma powers differential expression analyses for RNA-sequencing and microarray studies. *Nucleic Acids Res.* **43**, e47 (2015). 25605792 10.1093/nar/gkv007 PMC4402510

[122] Rossi, F. et al. Co-expression-based models improve eQTL predictions for transcriptome-wide association studies and highlight new schizophrenia-associated genes (v.1.0). *Zenodo*10.5281/zenodo.14959416 (2026). 10.1038/s41588-026-02646-3 PMC13364706 42332269

