# Supplementary material for: Co-expression-based models improve eQTL predictions for transcriptome-wide association studies and highlight new schizophrenia-associated genes
Source: Nat Genet. 2026 Jun 22;58(7):1559–72. doi: 10.1038/s41588-026-02646-3 (PMC13364706; doi:10.1038/s41588-026-02646-3)
Supplement: Supplementary file 1 — Supplementary Notes and Supplementary Figs. 1–28 (Supplementary Tables 1–9 provided separately as an Excel file). [file 41588_2026_2646_MOESM1_ESM.pdf]

nature genetics

Supplementary information https://doi.org/10.1038/s41588-026-02646-3

Co-expression-based models improve eQTL predictions for transcriptome-wide association studies and highlight new schizophrenia-associated genes

In the format provided by the authors and unedited

Supplementary Information

Table of Contents

Supplementary Notes 1
Supplementary Figures 13
References 41

Supplementary Notes

Ethics statement

The research described herein complies with all relevant ethical regulations. Postmortem human brain tissues from the LIBD collection were primarily obtained by autopsy from the Offices of the Chief Medical Examiner of the District of Columbia and of the Commonwealth of Virginia, Northern District, all with informed consent from the legal next of kin (protocol 90-M-0142 approved by the National Institute of Mental Health (NIMH)/National Institutes of Health (NIH) Institutional Review Board). The National Institute of Child Health and Human Development Brain and Tissue Bank for Developmental Disorders (https://medschool.umaryland.edu/BTBank) provided infant, child, and adolescent brain tissue samples under the NO1-HD-43368 and NO1-HD-4-3383 contracts. Additionally, donations of postmortem human brain tissue from patients with SCZ were provided with informed consent by next of kin from the Office of the Chief Medical Examiner for the State of Maryland under protocol number 12–24 from the State of Maryland Department of Health and Mental Hygiene and from the Office of the Medical Examiner, Department of Pathology, Homer Stryker, Maryland School of Medicine under protocol number 20111080 from the Western Institute Review Board. The Institutional Review Board of the University of Maryland at Baltimore and the State of Maryland approved the study protocol. The Lieber Institute for

Brain Development (LIBD) received the tissues by donation under the terms of a material transfer agreement.

The replication cohort of GTEx data adhered to the requirements established in the GTEx pilot study requirments[1]. Explicit authorization for tissue donation was obtained from the next-of-kin or legally authorized representatives, even though research on deceased individuals is not legally classified as human subjects research. Biospecimen Source Sites (BSS) either submitted a research protocol for IRB review or determined that further review was unnecessary. Consent was obtained in person and over the phone, and a sub-study evaluated donor families' concerns regarding the process. Training materials were developed to support ethical interactions with donor families. To safeguard privacy and confidentiality, only de-identified data were shared, with strict access controls based on user roles. The project implemented a Material Transfer and Data Use Agreement to define responsibilities for privacy protection across all parties involved. The template agreement is posted publicly at http://biospecimens.cancer.gov/global/pdfs/caHUB_Material_Transfer_and_Data_Use_Agreement_072512-508.pdf.

For the CMC replication cohort, ethical approvals are detailed in the original paper[2]. Briefly, the "MSSM" brain specimens were obtained through the Mount Sinai NIH Brain Bank and Tissue Repository (NBTR) (http://icahn.mssm.edu/research/labs/neuropathology-and-brain-banking), which obtains brain specimens from the Pilgrim Psychiatric Center, collaborating nursing homes, Veteran Affairs Medical Centers and the Suffolk County Medical Examiner's Office. Informed consent is obtained from the next of kin. The brain bank procedures are approved by the ISMMS IRB and exempted from further IRB review due to the collection and distribution of postmortem specimens. The "Pitt" sample were obtained through the University of Pittsburgh Brain Tissue Donation Program during routine autopsies conducted at the Allegheny County Office of the Medical Examiner (Pittsburgh) following the consent of the next of kin. All procedures for Pitt samples have been approved by the University of Pittsburgh's Committee

for the Oversight of Research involving the Dead and Institutional Review Board for Biomedical Research. The "Penn" brain specimens were collected through the University of Pennsylvania Brain Bank of Psychiatric illnesses and Alzheimer's Disease Core Center (http://www.med.upenn.edu/cndr/biosamples-brainbank.shtml). All procedures for Penn are approved by the Committee on Studies Involving Human Beings of the University of Pennsylvania, and the use of control postmortem tissues was considered exempted research in accordance with CFR 46.101 (b), item 65 of Federal regulations and University policy.

Ethical approval protocols for each of the 62 PGC wave 3 study sites used in this research are described in the Supplementary Cohort Descriptions from Trubetskoy et al[3].

LIBD post-mortem sample description

Dorsolateral prefrontal cortex (DLPFC) samples were obtained from Brodmann Area (BA) 9/46 at the level of the rostrum of the corpus callosum[4-6]. Hippocampus (HP) samples included the mid-hippocampus proper, with dissections covering the dentate gyrus, CA3, CA2, CA1, and the subicular complex[5]. The caudate nucleus (CN) was dissected from its anterior "head" portion, representing the part most tightly connected to the prefrontal cortex[7]. Amygdala samples[6,8] were dissected from the medial temporal lobe, covering all subnuclei at the level of the largest circumference. Subgenual anterior cingulate cortex (sACC) samples were dissected from the ventral part of the corpus callosum to the dorsal part of the orbital frontal cortex (BA11)[8].

All amygdala, CN, dACC and sACC samples underwent sequencing via the Illumina Ribo-Zero Kit. For DLPFC, RNA-seq was performed using the Illumina Ribo-Zero kit, and the RNeasy kit (Qiagen). RNA-seq for HP samples was performed via the Illumina Ribo-Zero kit and the Illumina TruSeq Stranded Total RNA Library Prep Human/Mouse/Rat kit. When DLPFC or HP data were available with both techniques, we retained those with maximum RNA Integrity Number (RIN) and Ribo-Zero kit protocol.

Lambda tuning (MODULE training)

We used the same fold indices that we used in the "co-eQTL discovery" step to fine-tune the Enet lambda parameter. We used the *lambdaseq* function from the *tsutils* (v0.9.4) package to generate a sequence of lambdas for CV testing (alpha = 0.5, lambdaRatio = 1e-02, nLambda = 100). In each training set, we computed the PC1 and projected its loadings onto the testing set to obtain a testing PC1. For reproducibility, we set a seed and generate random 4-fold indices to perform an inner loop CV and tuned the optimal lambda for each outer loop. We fit the model on the projected testing-fold PC1 with the best lambda and computed various performance statics, including mean square error (MSE), R^2, adjusted R^2 and the Pearson's correlation between the observed target gene-expression level and the predicted fold PC1. After CV, we selected the optimal nested-CV lambda, corresponding to the fold with the minimum average MSE. Before training the final model on the genotype and PC1 computed on all data, we first controlled for the correlation sign between the gene and the PC1 of the co-expression matrix, as the sign of the PC1 can be arbitrary. If Pearson's correlation was negative, we inverted the sign of the PC1. We computed Pearson's correlation between the gene-level expression and the cv model when fitted on the training genotype.

SNP-proxy Linkage Disequilibrium

In this analysis, the objective was to maximize the predictive accuracy of all predictive models by compensating for the absence of predictive SNPs in the testing datasets. To achieve this, we identified SNPs that were in high LD with the missing SNPs from our predictive models. Using the 1000 Genomes Project[9] data as a reference, and the PLINK v2.00[10] we computed the LD values for all potential proxy SNPs within a 500 kb around each missing SNP with the following command:

```
--ld-snp-list SNPs_mismatch.txt --ld-window 1000 --ld-window-kb 500 --ld-window-r2 0.8 --out
```

```
--pfile g1000/g1000_eur --r-unphased
```

These proxy SNPs were then integrated into our models as substitutes for the missing SNPs. By incorporating these correlated proxies, we aimed to preserve the integrity and efficiency of our SNP-based predictions, ensuring that our models remain robust and accurate despite variations in SNP availability across different datasets.

Sample-size sensitivity analysis for CIS and EpiXcan

We evaluated the effect of training sample size on *cis*-model performance using LIBD, GTEx, and CMC cohorts. The goal was to estimate how the number of predictable genes and cross-validated accuracy scale with *n*, and whether enforcing cross-dataset replication would disproportionately penalize *cis* models due to reduced effective training size. For every pair of datasets in a region, we identified the intersection of predictable genes and calculated replicability rate as the proportion of shared genes relative to each dataset's total. For each between-dataset comparison we computed the sample-size ratio (max/min) and related it to (i) the absolute difference in mean adjusted R^2 between datasets and (ii) the replicability rate. Simple linear models (*lm* function in R) were fit with sample-size ratio as the predictor; 95% confidence intervals, significance and R^2 were obtained from the model fits.

PGC weight Ratio and Connectivity Enrichment

To evaluate whether genes more heavily influenced by SCZ-associated variants exhibit increased connectivity to PGC3-prioritized genes[3], we performed a permutation-based test of monotonic enrichment across quintiles of a PGC-weight metric.

PGC-weight Metric Calculation. For each gene predicted by CIS, EpiXcan, and MODULE models, we computed a PGC-weight metric defined as:

$$\text{PGC-weight ratio} = \left(\frac{\sum_{\text{PGC3 SNPs}} |\text{ model weight }|}{\sum_{\text{all SNPs}} |\text{ model weight }|}\right)$$

This metric captures both the relative contribution and density of SCZ-associated SNPs ($p < 0.05$ in PGC3 summary statistics[3]) to the gene's expression prediction.

Connectivity Quantification. Gene-wise connectivity scores were obtained from Borcuk et al.[11] who constructed transcriptomic co-expression networks across five brain regions (amygdala, DLPFC, CN, HP, sACC). Each score reflects the strength of connectivity between a given gene and the set of 120 PGC3-prioritized genes[3].

Quintile Binning and Trend Assessment. Genes were binned into five equal-sized quintiles based on their PGC-weight metric. For each model-region pair, we computed the mean connectivity within each quintile, resulting in a 5-point trajectory.

To assess monotonic trends, we calculated:

- Linear regression slope across quintile indices (1 to 5)
- Spearman's rank correlation coefficient (ρ) between quintile index and mean connectivity

Permutation Testing. To generate a null distribution, we performed 1,000 gene-level permutations per model-region pair. In each permutation:

1. Genes were randomly reassigned to quintiles (preserving bin sizes).
2. Mean connectivity was recalculated per permuted quintile.
3. Both the linear slope and Spearman's ρ were recomputed.

Empirical two-sided *p*-values were defined as the proportion of permuted statistics whose absolute value equaled or exceeded the observed statistic. This approach does not assume linearity and directly tests whether the observed ordering of quintile means departs from chance.

MAGMA Z-score derivation

We performed gene-based association analyses using MAGMA v1.09b[12] to derive gene-level Z-scores. SNP-to-gene mapping was conducted with the default MAGMA annotation files, using a window of 35 kb upstream and 10 kb downstream of each gene. As input, we used SNP *p*-values from the PGC3 SCZ GWAS summary statistics[3], together with the 1000 Genomes European reference panel[9] to account for linkage disequilibrium. MAGMA aggregates SNP-level association statistics within the defined cis-window, correcting for LD, to produce a gene-level *p*-value, which is then transformed into a standardized Z-score. In our study, these Z-scores were considered the MAGMA importance score, representing the strength of cis-genetic association between each gene and SCZ risk.

Cross-dataset replication of predictive models

To assess the reproducibility of predictive weights across independent brain collections, we evaluated training-level performance and cross-dataset replication for CIS, EpiXcan, INGENE, and MODULE using LIBD, GTEx, and CMC data.

Training-level overview. Sample sizes varied substantially, from 116 donors in CMC amygdala to nearly 600 in LIBD DLPFC (Supplementary Figure 3a), affecting eQTL discovery power and cross-validation metrics[13,14]. We compared the number of predictable genes (Supplementary Figure 3b) and cross-validated performance across datasets (Supplementary Figure 3c). Across regions, both *cis*- and *trans*-models exhibited heterogeneous CV adjusted R^2 values that reflected sample-size differences

rather than intrinsic model behaviour. Mean CV adjusted R^2 ranged from 0.11–0.78 for CIS, 0.10–0.32 for EpiXcan, 0.10–0.34 for INGENE, and 0.15–0.20 for MODULE (Supplementary Figure 3c). Because such variability may inflate apparent model fit, we next quantified independent cross-dataset replication for INGENE and MODULE models as a more reliable measure of generalizability.

Cross-dataset replication. We assessed reproducibility by comparing predictions across datasets using LIBD as the common reference (see Methods – *MODULE and INGENE Cross-dataset Training* for detailed procedures). Briefly, each gene predicted expression values from GTEx, or CMC were correlated with the corresponding predictions from LIBD. Two directional comparisons were performed: (i) GTEx → LIBD versus LIBD → LIBD, and (ii) CMC → LIBD versus LIBD → LIBD. Genes were retained as replicable if the Pearson correlation between external and LIBD predictions exceeded 0. This design isolates cross-cohort consistency while holding sample size and model complexity constant.

Across brain regions, both INGENE and MODULE displayed broad cross-dataset overlaps (Supplementary Figure 2a-b). For INGENE, reproducible gene counts ranged from 9,590 in amygdala to 16,560 in DLPFC. For MODULE, reproducibility ranged from 5,807 in HP to 12,599 in sACC. In regions represented in both external datasets (dACC, DLPFC, sACC), GTEx and CMC each contributed comparably to the reproducible gene pool: for INGENE, GTEx-validated genes accounted for 57–78 % and CMC-validated for 87–90 % of the total reproducible set (shared overlap ≈ 50–65 %); for MODULE, the respective contributions were 64–70 % for each dataset (shared overlap ≈ 30–40 %) (Supplementary Figure 2a–b).

Anchoring replication to LIBD as a shared reference and applying this correlation-based filtering ($r > 0$) delineated a subset of gene-level predictions consistently reproduced across independent postmortem brain cohorts. This subset was carried forward for downstream postmortem validation and coTWAS analyses.

Sample-size sensitivity of cis-based predictors

To evaluate the influence of training-sample size on the reproducibility of *cis*-based models, we examined cross-cohort consistency for CIS and EpiXcan predictors across LIBD, GTEx, and CMC datasets (see Methods: *Sample-size sensitivity analysis for CIS and EpiXcan*). Analyses quantified the relationship between training-sample size ratios and cross-dataset differences in prediction accuracy (Supplementary Figure 4).

1. CIS shows strong sample-size dependence. Across brain regions, CIS performance differences scaled positively with the ratio of training-sample sizes ($R^2 = 0.476$, $p = 0.019$; Supplementary Figure 4a). At the gene level, retention of overlapping predictions declined proportionally to sample-size imbalance (Supplementary Figure 4b). In DLPFC, where LIBD and CMC sample sizes were comparable (Supplementary Figure 3a), CIS retained ~53% of overlapping genes. In more unbalanced comparisons, retention dropped to ~25%. These results indicate that CIS predictors are particularly sensitive to differences in discovery-cohort size and may overfit small training datasets.

2. EpiXcan exhibits reduced sample-size sensitivity. EpiXcan showed no significant association between sample-size ratio and cross-cohort performance differences ($R^2 = 0.033$, $p = 0.62$; Supplementary Figure 4c). In the balanced DLPFC comparison (CMC–LIBD), EpiXcan retained > 55 % of overlapping genes, and in unbalanced regions the decline was modest (Supplementary Figure 4d). Across all regions, retention ranged from ~35 % to 60 %, consistently exceeding CIS.

3. Limited overlap across cohorts. Despite improved stability, overall cross-cohort overlap remained modest. Even under balanced sample sizes (e.g., CMC–LIBD DLPFC), only about half of overlapping genes were retained, and in most other pairs both CIS and EpiXcan lost 70–85 % of predictions. This limited reproducibility underscores the impact of dataset-specific variance on *cis*-based models.

Benchmarking with BGW-TWAS and MOSTWAS

To benchmark our *cis*- and *trans*-aware predictive frameworks against previously established models, we compared them with BGW-TWAS[15] (models downloaded from https://www.synapse.org/Synapse:syn22316791/wiki/605024) and MOSTWAS[16] (weights downloaded from https://zenodo.org/records/4314067), two reference methods that incorporate distal regulatory information. Analyses were performed in the DLPFC, the only brain region where all reference models were available.

BGW-TWAS model integration and application in testing datasets. The BGW models, trained in the ROS/MAP cohort[15], comprised approximately 28 million SNPs (6 million unique). SNP genomic coordinates were lifted from GRCh37 to GRCh38, and multi-allelic variants and indels were removed (842,781 unique and ~3.5 million non-unique values excluded). To optimize prediction in the GTEx and CMC testing datasets, we incorporated proxy variants in linkage disequilibrium (LD $r^2 \geq 0.8$) for missing SNPs. Final SNP weights were computed as the product of the estimated effect size (β) and its posterior probability (pp), i.e., $\beta/SE \times pp$[15] , and stored in an SQLite database compatible with the MetaXcan[17] *Predict.py* implementation. Only SNPs present in each testing dataset were retained, and genes were classified based on the regulatory origin of their predictors as *cis*, *trans*, or *cis* + *trans*.

In GTEx, BGW predicted 34 *cis*, 12,540 *cis* + *trans*, and 1,523 *trans* genes (total = 14,097). In CMC, the respective counts were 59 *cis*, 12,513 *cis* + *trans*, and 1,518 *trans* genes (total = 14,090). Predicted gene expression values were matched to observed DLPFC expression by intersecting common genes (GTEx: 14,062; CMC: 13,501) and scaled prior to analysis.

Performance was quantified by Pearson's r and adjusted R^2 using linear regression, computed separately for *cis*, *cis* + *trans*, and *trans* genes. In GTEx, 6,098 had Pearson's $r > 0$ and adjusted $R^2 > 0$ (N (*cis)* = 21; N(*cis* + *trans)* = 5.581; N(*trans)* = 496), with a mean adjusted R^2 of 0.05. In CMC, 5,849

met the same thresholds (N(*cis)* = 32; N (*cis* + *trans)* = 5.366; N(*trans)* = 451), yielding a mean adjusted R^2 of 0.04 (Supplementary Figure 7c-d).

MOST-TWAS models integration and application in testing datasets. Across all genes, MeTWAS incorporated 673,247 SNPs (445,816 unique) and DePMA 700,843 SNPs (536,183 unique). Because allele and coordinate information were not provided, these were retrieved from BGW annotations and the GRCh37 reference genome, designating the effective allele as the alternative. SNP genomic coordinates were lifted from GRCh37 to GRCh38, and multi-allelic SNPs and indels were removed (N = 75,569 unique for both models). *MetaXcan* was applied directly to the weights, and all remaining preprocessing steps were identical to those used for BGW-TWAS.

For MeTWAS, *cis*/*trans* annotations were corrected based on genomic distance, with variants located ±1 Mb from the gene start/end (or on another chromosome) reclassified as *trans*. Of 910 genes provided, 755 passed quality control and were retained for analysis.

For MeTWAS, in GTEx predictions were obtained for 278 *cis*, 460 *cis* + *trans*, and 9 *trans* genes (total = 747).; in CMC, 282 *cis*, 455 *cis* + *trans*, and 8 *trans* genes were predicted (total = 745). Predicted expression values were matched to observed DLPFC expression (GTEx: 697 genes; CMC: 624) and scaled prior to evaluation. In GTEx, 268 genes (N(*cis)* = 111; N (*cis* + *trans)* = 154; N(*trans)* =3), had adjusted $R^2 > 0$ (mean $R^2 = 0.02$), while in CMC, 275 (N (*cis)* =128; N (*cis* + *trans)* = 144; N (*trans)* =3), satisfied thresholds (mean adjusted $R^2 = 0.012$).

For DePMA, 2,941 genes were provided without *cis*/*trans* annotation and were classified using the same genomic-distance rule (± 1 Mb form gene start/end as *trans*). In GTEx, predictions were obtained for 136 *cis*, 2,223 *cis* + *trans*, and 567 *trans* genes (total = 2,926). In CMC, 214 *cis*, 2,081 *cis* + *trans*, and 615 *trans* genes were predicted (total = 2,910).

Predicted values were matched to observed DLPFC expression (GTEx: 2,710 genes; CMC: 2,412) and scaled. In GTEx, 1,122 (N(*cis)* = 67; N(*cis* + *trans)* =885; N(*trans)* = 170), had Pearson's *r* >0 and adjusted $R^2 > 0$ (mean adjusted $R^2 = 0.032$), while in CMC, 1,094 (N(*cis)* =106; N (*cis* + *trans)* = 828; N(*trans)* = 160), survived both thresholds (mean adjusted $R^2 = 0.023$) (Supplementary Figure 7c-d).

Supplementary Figures

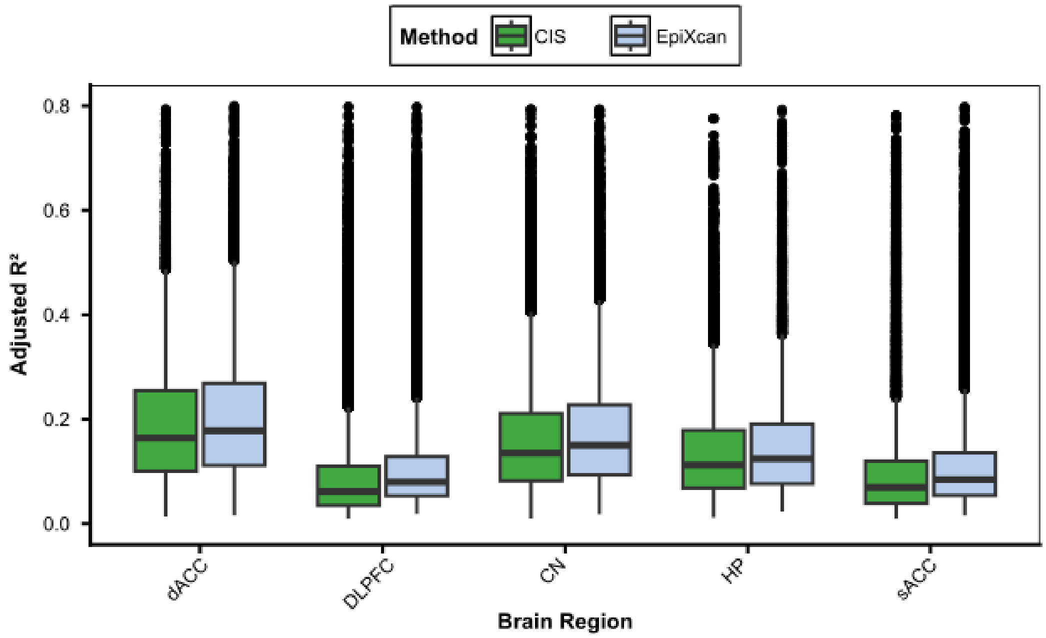

Supplementary Figure 1. EpiXcan improves cis-based prediction accuracy across brain regions. *Box plots show the distribution of cross-validated adjusted R^2 values (proportion of variance explained) for CIS (green) and EpiXcan (light blue) models trained in five LIBD brain regions: caudate nucleus (CN), dorsal anterior cingulate cortex (dACC), dorsolateral prefrontal cortex (DLPFC), hippocampus (HP), and subgenual anterior cingulate cortex (sACC). The central line represents the median; boxes indicate the interquartile range (25th–75th percentiles); whiskers extend to the most extreme values within 1.5× IQR; and points beyond the whiskers represent outliers. Sample sizes (n = number of genes) per region and model are: dACC (CIS, n = 13,681; EpiXcan, n = 14,000), DLPFC (CIS, n = 14,324; EpiXcan, n = 14,442), CN (CIS, n = 13,780; EpiXcan, n = 13,962), HP (CIS, n = 13,092; EpiXcan, n = 13,432), and sACC (CIS, n = 15,422; EpiXcan, n = 15,553).*

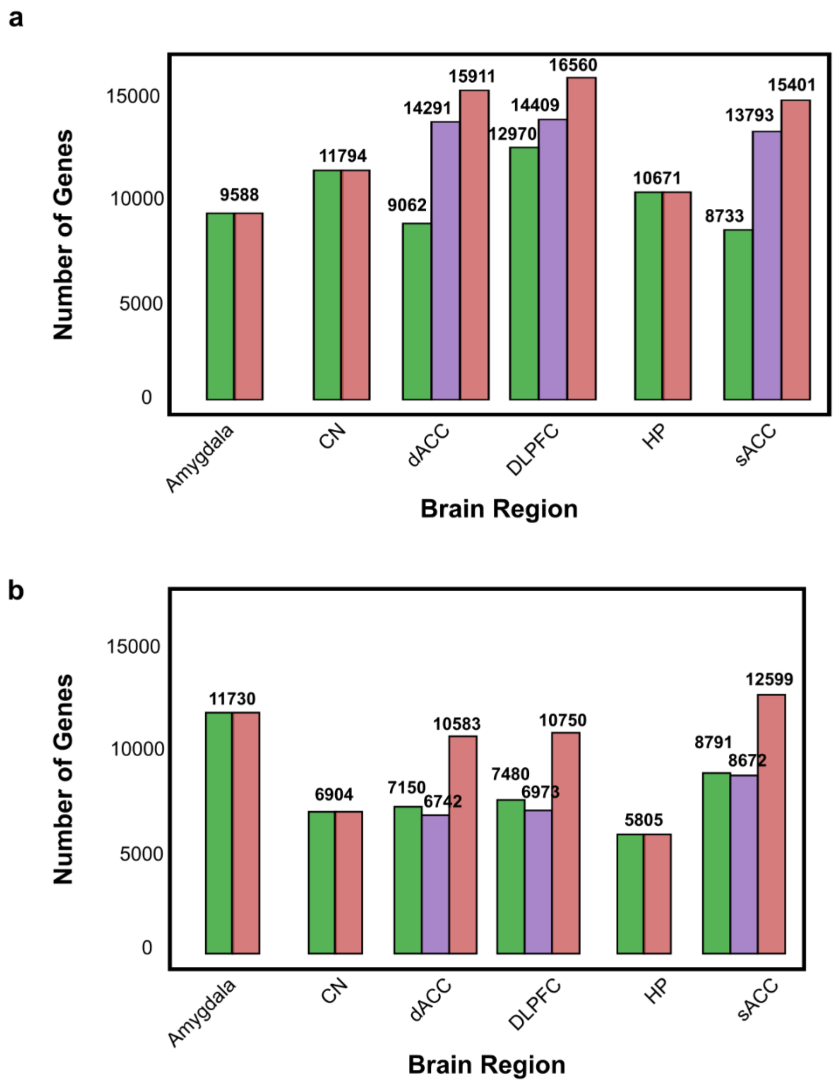

Supplementary Figure 2. Cross-dataset replication of trans-aware predictive models across brain regions. a*) INGENE and* ***b****) models were trained in LIBD and independently evaluated in GTEx and CMC to assess reproducibility of gene-level predictions across cohorts. Bars represent the number of genes (n = number of genes) satisfying the replication criterion, defined as a positive Pearson correlation (r > 0) between predicted gene expression in the external dataset (GTEx or CMC) and the corresponding LIBD-based prediction. Green bars indicate GTEx-evaluated predictions (GTEx → LIBD), violet bars indicate CMC-evaluated predictions (CMC → LIBD), and red bars denote the total number of reproducible genes across external datasets for each brain region.*

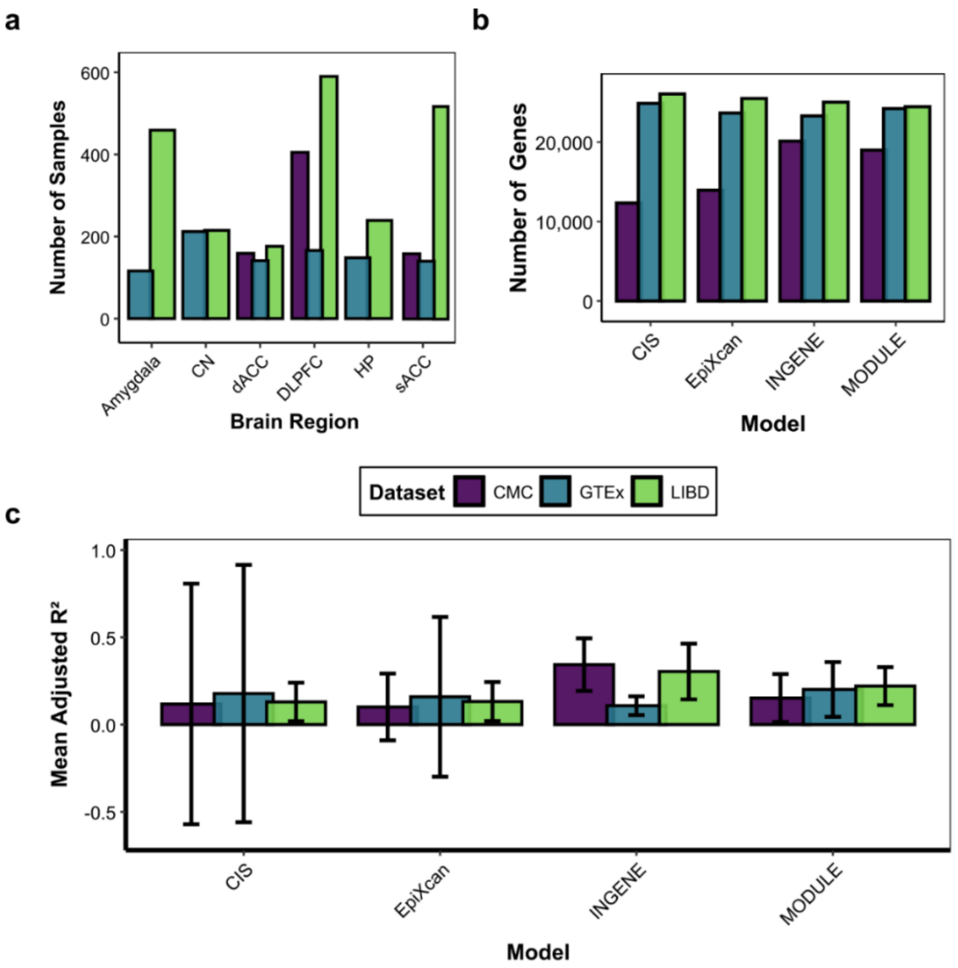

Supplementary Figure 3. Training performance of predictive models across cohorts and brain regions. ***a)*** *Number of samples (n = number of individuals) available for training across brain regions in CMC, GTEx, and LIBD, illustrating variability in sample size and statistical power.* ***b)*** *Number of unique genes (n = number of genes) included in predictive models across cohorts for each framework (CIS, EpiXcan, INGENE, MODULE). Sample sizes per dataset and model are: CIS (CMC, n = 12,343 genes; GTEx, n = 24,893 genes; LIBD, n = 26,066 genes), EpiXcan (CMC, n = 13,951 genes; GTEx, n = 23,666 genes; LIBD, n = 25,501 genes), INGENE (CMC, n = 20,132 genes; GTEx, n = 23,306 genes; LIBD, n = 25,031 genes), and MODULE (CMC, n = 18,991 genes; GTEx, n = 24,228 genes; LIBD, n = 24,462 genes).* ***c)*** *Mean cross-validated adjusted R^2 (proportion of variance explained) across genes for each model and dataset. Error bars represent ±1 standard deviation calculated across genes within each dataset–model combination.*

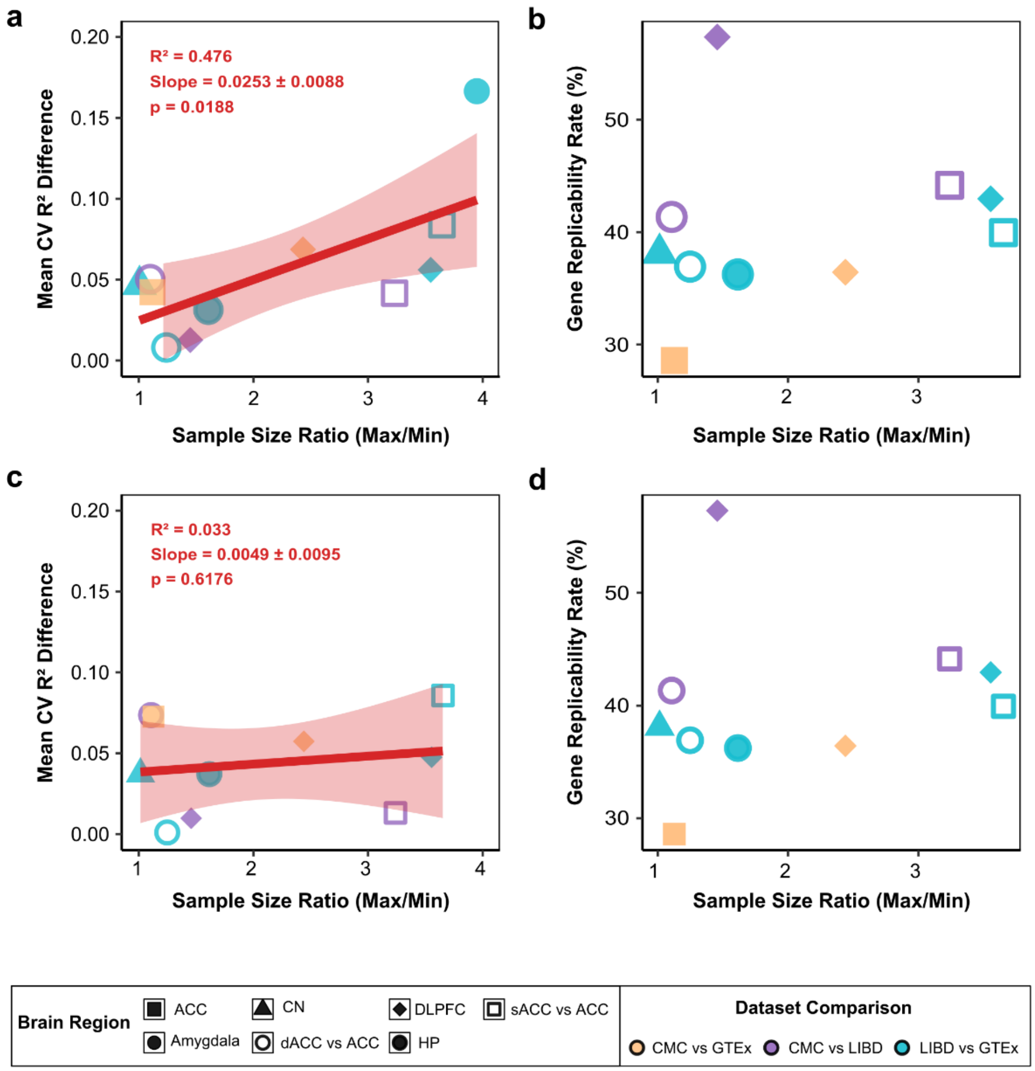

Supplementary Figure 4. Sample-size sensitivity of CIS and EpiXcan predictors. *a) Relationship between sample-size ratio (maximum/minimum training sample size across paired cohorts) and absolute difference in mean cross-validated adjusted R^2 between cohorts for CIS models. Each point represents one brain region–dataset comparison (n = number of cohort comparisons). The red line indicates the fitted linear regression (ordinary least squares), with shaded area representing the 95% confidence interval. Reported statistics correspond to the linear model (two-sided test).* ***b)*** *Gene-level replicability rate (%) for CIS across cohort comparisons as a function of sample-size ratio. Each point represents the percentage of overlapping predictable genes retained across paired cohorts.* ***c)*** *Relationship between sample-size ratio and absolute difference in mean cross-validated adjusted R^2 for EpiXcan models. Linear regression (two-sided test) shows no significant association ($R^2 = 0.033$, slope = 0.0049 ± 0.0095 (standard error), $p = 0.6176$); shaded area indicates the 95% confidence interval.* ***d)*** *Gene-level*

replicability rate (%) for EpiXcan across cohort comparisons. Each point represents one brain region–dataset comparison.

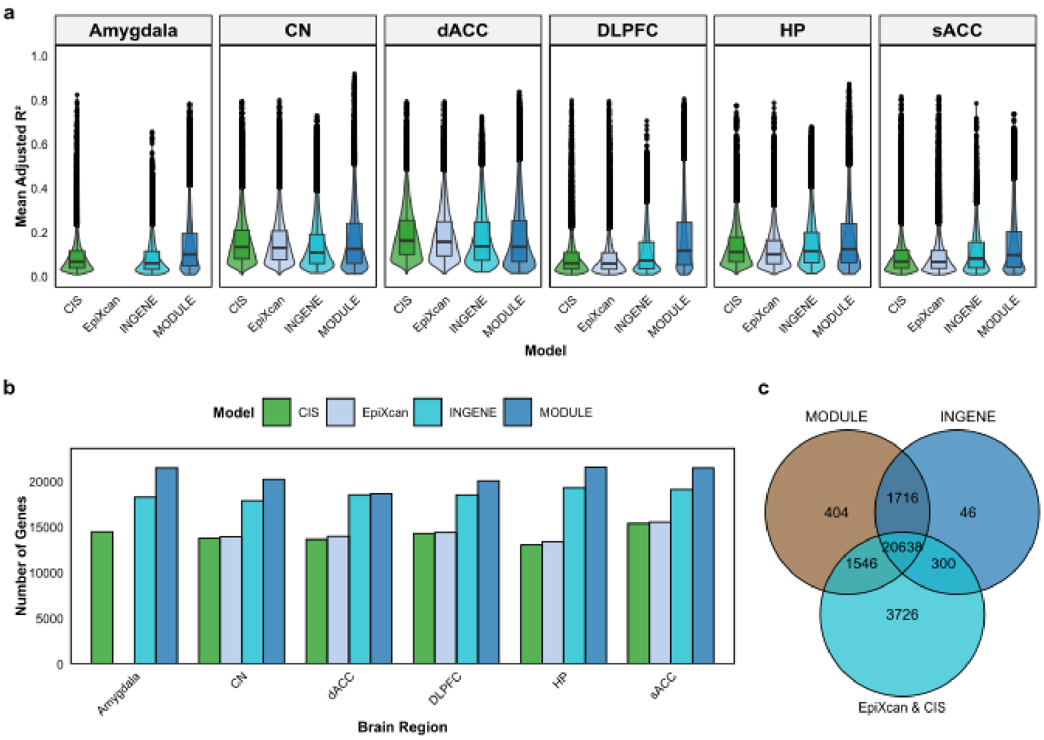

Supplementary Figure 5. Comparison of CIS, EpiXcan, INGENE and MODULE model training performance. a) *Violin and box plots showing the distribution of cross-validated adjusted R^2 values (proportion of variance explained) for CIS (green), EpiXcan (light blue), INGENE (cyan), and MODULE (dark blue) models across brain regions. Each distribution reflects gene-level prediction performance (n = number of genes per model and region; see panel b). Box plots display the median (centre line), interquartile range (box bounds, 25th–75th percentiles), whiskers extending to the most extreme values within 1.5× IQR, and points indicating outliers.*
b) *Number of genes (n = number of genes) retained within each predictive framework per brain region after applying the predefined cross-validation performance threshold (see Methods).* ***c)*** *Venn diagram showing overlap of predicted genes pooled across brain regions among the combined cis models (CIS + EpiXcan) and trans models (INGENE and MODULE).*

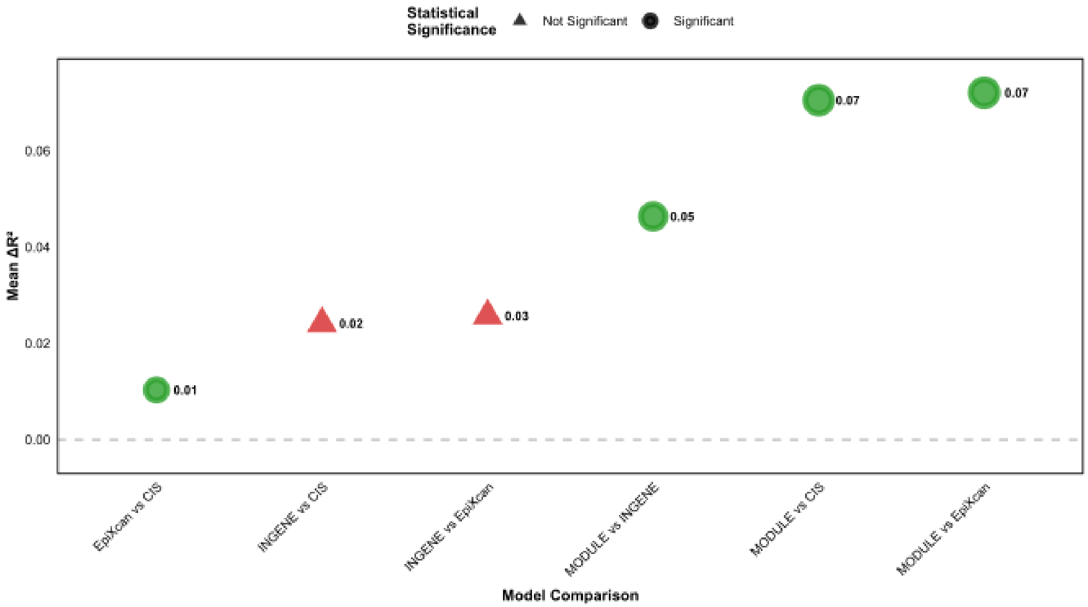

Supplementary Figure 6. Pairwise comparison of predictive performance across models*. Mean differences in cross-validated adjusted R^2 (ΔR^2) between predictive models are shown for n = 15,232 genes commonly predicted across all models and pooled across brain regions. For each comparison, ΔR^2 represents the mean paired difference in adjusted R^2 across genes (positive values indicate higher performance of the first-listed model). Statistical significance was assessed using a one-sided paired Wilcoxon signed-rank test testing whether the first-listed model outperformed the second-listed model. Green circles denote statistically significant differences ($\alpha = 0.05$), and red triangles denote non-significant comparisons. Exact p-values are: EpiXcan vs CIS (2.06×10^{-20}), INGENE vs CIS (0.155), INGENE vs EpiXcan (0.154), MODULE vs INGENE (5.04×10^{-16}), MODULE vs CIS (3.02×10^{-3}), and MODULE vs EpiXcan (1.46×10^{-4}). No correction for multiple comparisons was applied.*

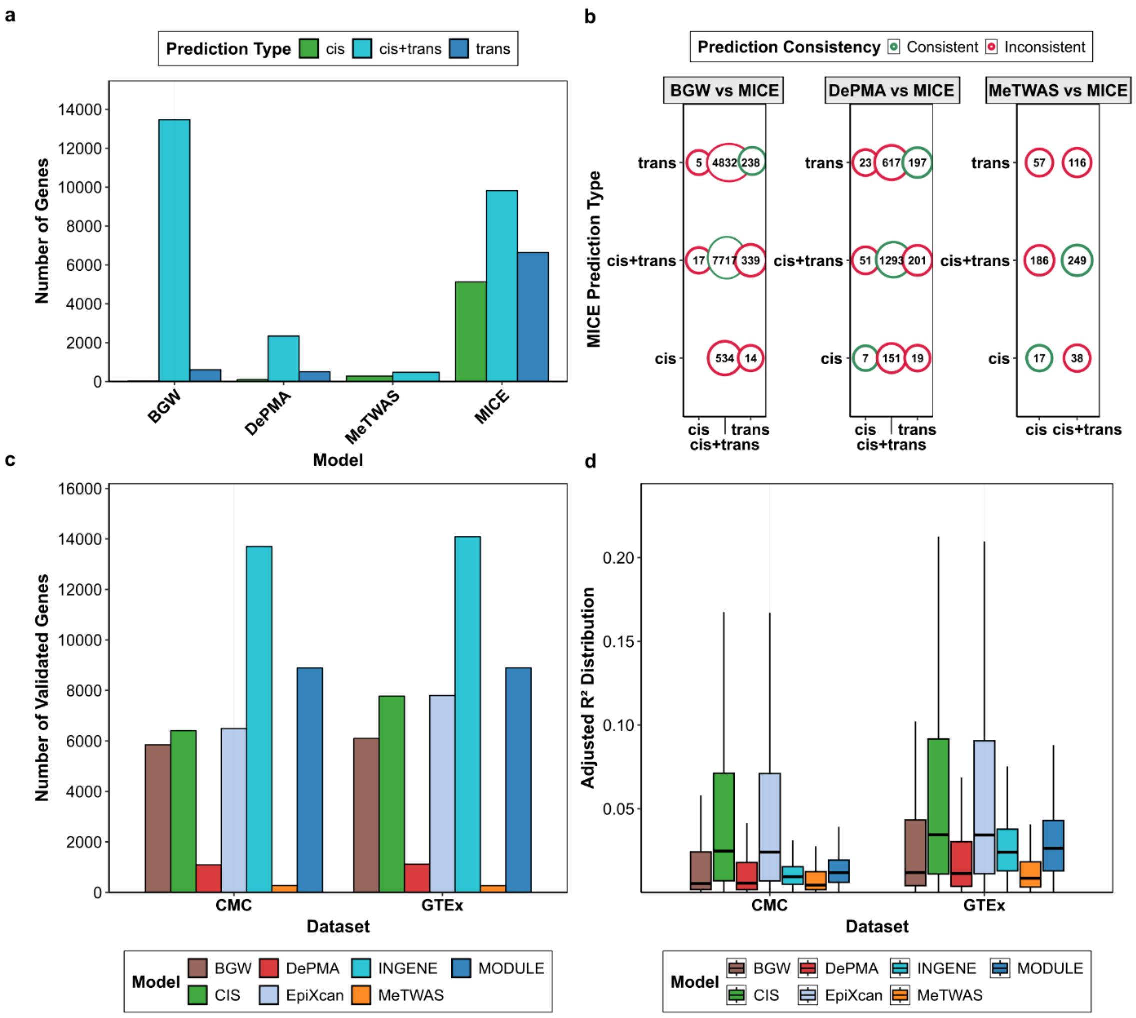

Supplementary Figure 7. Benchmark comparison with MOSTWAS and BGW-TWAS models. a) *Number of genes (n = number of genes) with trained prediction models in DLPFC across frameworks (BGW-TWAS, DePMA, MeTWAS, CIS, EpiXcan, INGENE, and MODULE).* ***b)*** *Consistency of gene-level regulatory classification between MICE (CIS/EpiXcan/INGENE/MODULE framework) and MOSTWAS/BGW-TWAS models. Genes were classified as cis, cis+trans, or trans based on regulatory contribution within each framework. Green circles indicate consistent classification across methods, and red circles indicate inconsistent classification. Numbers within circles represent gene counts.* ***c)*** *Number of validated genes (n = number of genes) in independent testing cohorts (CMC and GTEx). Genes were considered validated if Pearson correlation between predicted and observed expression was > 0 and adjusted $R^2 > 0$.* ***d)*** *Distribution of adjusted R^2 values in testing datasets (CMC and GTEx) for validated genes. Box plots display the median (centre line), interquartile range (25th–75th percentiles), whiskers extending to the most extreme values within 1.5× IQR, and points beyond the whiskers representing*

outliers. Sample sizes are: BGW-CMC (n = 5,849 genes), CIS-CMC (n = 6,406 genes), DePMA-CMC (n = 1,094 genes), EpiXcan-CMC (n = 6,492 genes), INGENE-CMC (n = 13,704 genes), MeTWAS-CMC (n = 275 genes), MODULE-CMC (n = 8,892 genes); BGW-GTEx (n = 6,098 genes), CIS-GTEx (n = 7,777 genes), DePMA-GTEx (n = 1,122 genes), EpiXcan-GTEx (n = 7,797 genes), INGENE-GTEx (n = 14,090 genes), MeTWAS-GTEx (n = 268 genes), MODULE-GTEx (n = 8,895 genes).

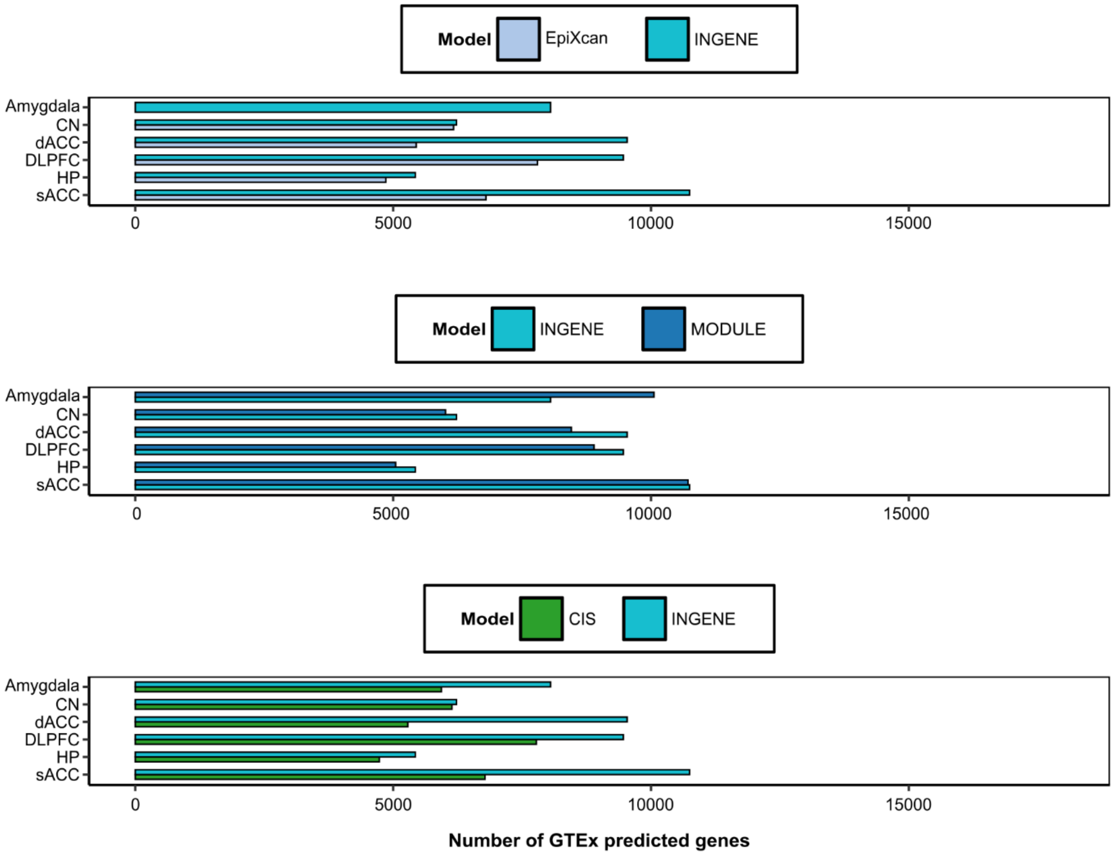

Supplementary Figure 8. Replication of predictive models in the GTEx testing dataset across brain regions. *Horizontal bar plots show the number of genes (n = number of genes) successfully predicted in the GTEx external dataset using models trained in LIBD. Genes were considered predicted if the correlation between predicted and observed expression was positive (Pearson $r > 0$) and adjusted $R^2 > 0$. Counts are shown for CIS (green), EpiXcan (light blue), INGENE (cyan), and MODULE (dark blue) across brain regions.*

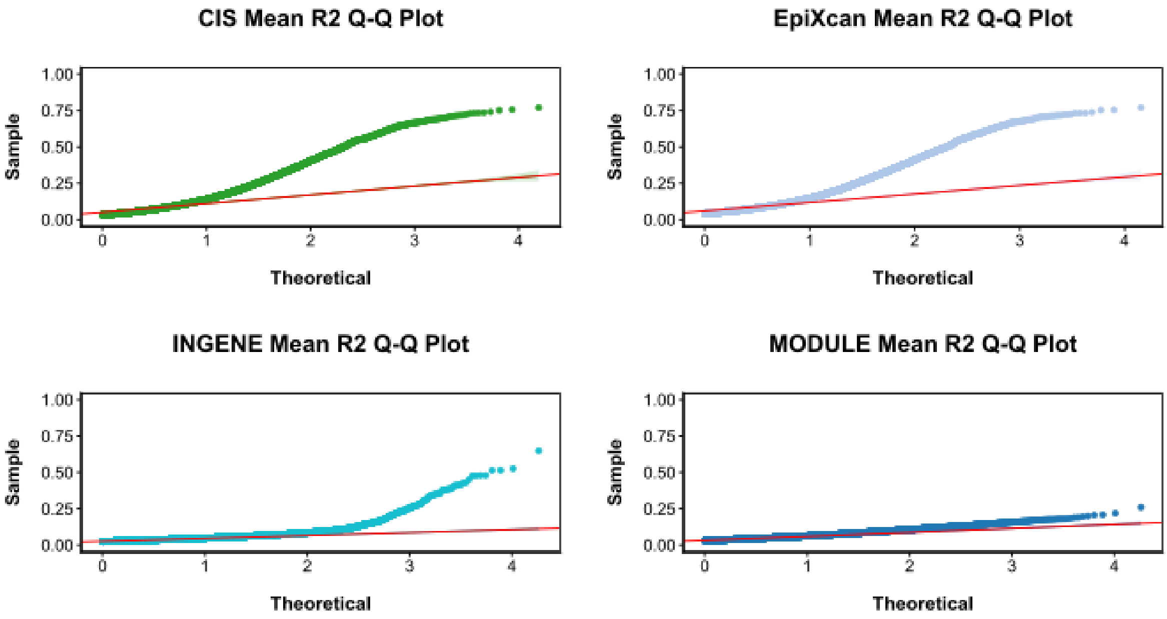

Supplementary Figure 9. Quantile–Quantile (Q–Q) plots of model performance in the GTEx testing dataset. *Q–Q plots comparing the distribution of gene-level adjusted* R^2 *values obtained in GTEx for CIS (green), EpiXcan (light blue), INGENE (cyan), and MODULE (dark blue) models trained in LIBD. Each point represents one gene (n = number of genes evaluated per model in GTEx; see Supplementary Figure 8). The y-axis shows observed adjusted* R^2 *values, and the x-axis shows theoretical quantiles from a standard normal distribution. The red line indicates the expected relationship under normality. Deviation from the reference line reflects departure of the observed* R^2 *distribution from a normal distribution.*

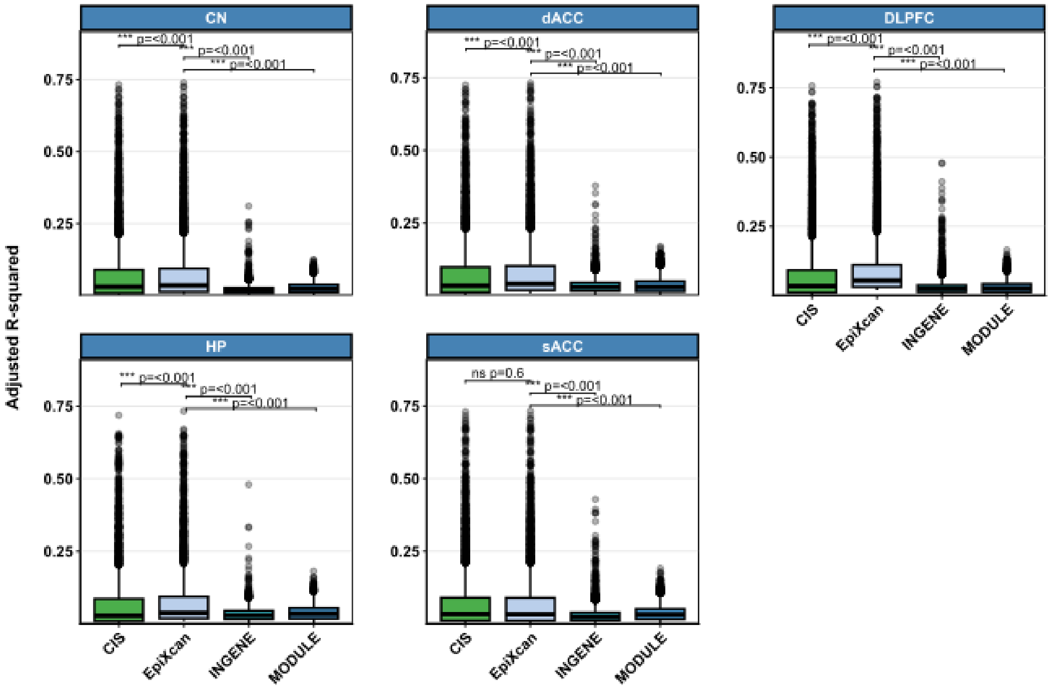

Supplementary Figure 10. Distribution of adjusted R^2 across prediction models by brain region. *Box plots show gene-level cross-validated adjusted R^2 values obtained in the GTEx testing dataset for CIS, EpiXcan, INGENE, and MODULE models trained in LIBD. For each box plot, the centre line denotes the median; box limits represent the interquartile range (25th–75th percentiles); whiskers extend to the most extreme values within 1.5 × IQR; and points beyond the whiskers indicate outliers. Brackets indicate one-sided paired Wilcoxon signed-rank tests comparing gene-level performance of EpiXcan to each alternative model within the same set of genes (paired across models). For each comparison, n denotes the number of paired genes included in the test. Exact p-values (unadjusted for multiple comparisons) are as follows: CN: EpiXcan vs MODULE (n = 1,404 genes, $p = 1.4 \times 10^{-27}$); EpiXcan vs INGENE (n = 1,479 genes, $p < 1 \times 10^{-50}$); EpiXcan vs CIS (n = 5,616 genes, $p < 1 \times 10^{-50}$). dACC: EpiXcan vs MODULE (n = 1,701 genes, $p = 2.5 \times 10^{-24}$); EpiXcan vs INGENE (n = 2,026 genes, $p = 6.5 \times 10^{-32}$); EpiXcan vs CIS (n = 4,908 genes, $p < 1 \times 10^{-50}$). DLPFC: EpiXcan vs MODULE (n = 2,587 genes, $p < 1 \times 10^{-50}$); EpiXcan vs INGENE (n = 2,948 genes, $p < 1 \times 10^{-50}$); EpiXcan vs CIS (n = 7,420 genes, $p < 1 \times 10^{-50}$). HP: EpiXcan vs MODULE (n = 934 genes, $p = 1.3 \times 10^{-7}$); EpiXcan vs INGENE (n = 1,018 genes, $p = 1.4 \times 10^{-12}$); EpiXcan vs CIS (n = 3,916 genes, $p < 1 \times 10^{-50}$). sACC: EpiXcan vs MODULE (n = 2,837 genes, $p = 4.0 \times 10^{-10}$); EpiXcan vs INGENE (n = 3,014 genes, $p = 1.1 \times 10^{-37}$); EpiXcan vs CIS (n = 6,546 genes, p = 0.64).*

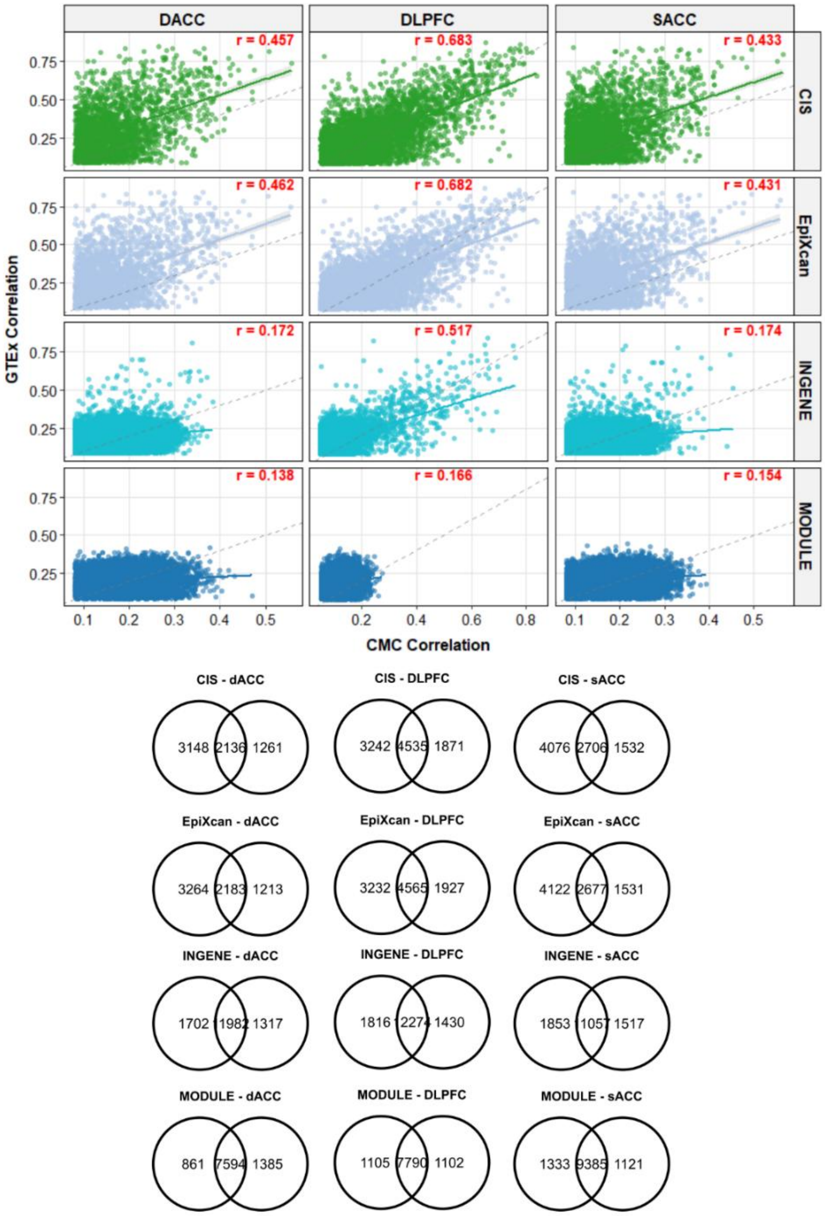

Supplementary Figure 11. Cross-cohort replicability of cis- and trans-prediction models. *Prediction performance of LIBD-trained models was evaluated independently in CMC and GTEx. Top panels show scatterplots of gene-level predictive accuracy in CMC (x-axis) versus GTEx (y-axis), where predictive accuracy is defined as the Pearson correlation (r) between predicted and observed expression for each gene. Each point represents one gene predicted in both cohorts (n = number of genes in the intersection for each model–region pair; see corresponding Venn diagrams). Bottom panels show Venn diagrams indicating the number of genes predicted in CMC (right circle), GTEx (left circle), and their intersection for each model and brain region (dACC, DLPFC, sACC).*

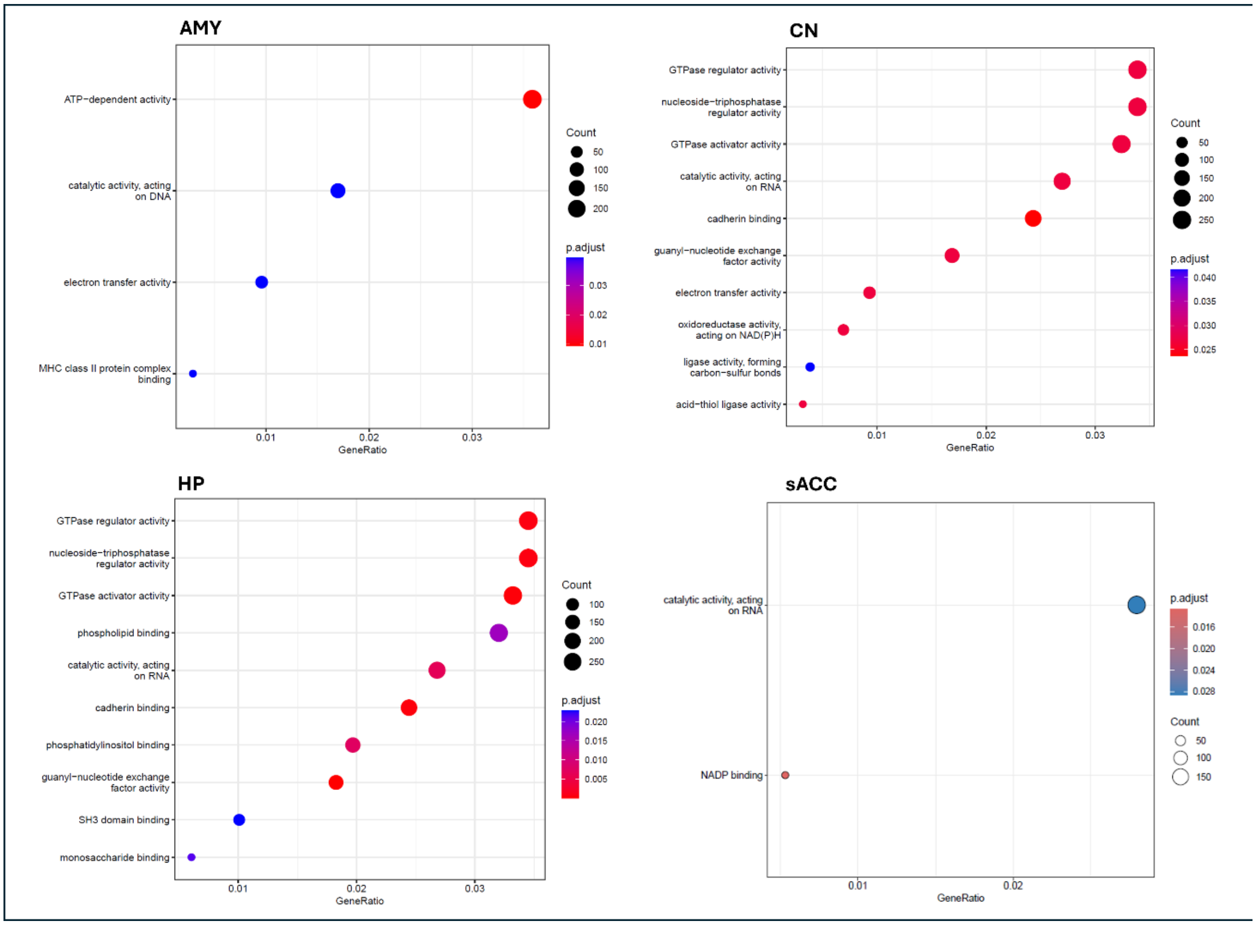

Supplementary Figure 12. Gene Ontology (GO) molecular function enrichment analysis of GTEx eGenes. Bubble plots show enriched GO molecular function categories for GTEx eGenes in amygdala (AMY), caudate nucleus (CN), hippocampus (HP), and subgenual anterior cingulate cortex (sACC). For each region, enrichment was tested using a two-sided hypergeometric test based on the set of GTEx eGenes as input (n = number of eGenes per region; see Methods) against the corresponding expressed gene background. P-values were adjusted for multiple comparisons within each region using the Benjamini–Hochberg false discovery rate (FDR) procedure.The x-axis shows the gene ratio, defined as the proportion of input genes assigned to a given GO category (number of overlapping genes divided by total input genes). Bubble size represents the number of overlapping genes (“Count”), and colour indicates the FDR-adjusted p-value.

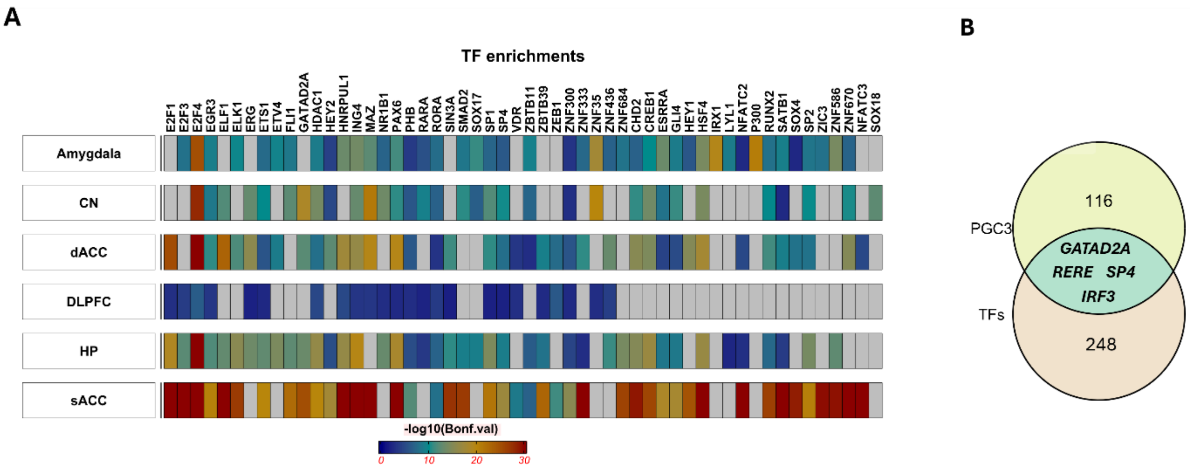

Supplementary Figure 13. Regulome enrichment analysis of GTEx cis-eGenes for MODULE trans-eQTLs. a) Transcription factor (TF) enrichment across brain regions (amygdala, CN, dACC, DLPFC, HP, sACC). For each region, enrichment of TF target sets among GTEx cis-eGenes (n = number of cis-eGenes per region; see Methods) was assessed using a one-sided hypergeometric test against the corresponding gene background. The top 20 TFs ranked by significance within each region are displayed. P-values were adjusted for multiple comparisons within each region using Bonferroni correction, and colour intensity represents −log10(Bonferroni-adjusted p-value). Grey blocks indicate TFs that did not remain statistically significant after correction. **b)** Venn diagram showing overlap between TFs significantly enriched in at least one brain region (after Bonferroni correction) and the 120 PGC3-prioritized genes. Numbers indicate gene counts within each set and their intersection.

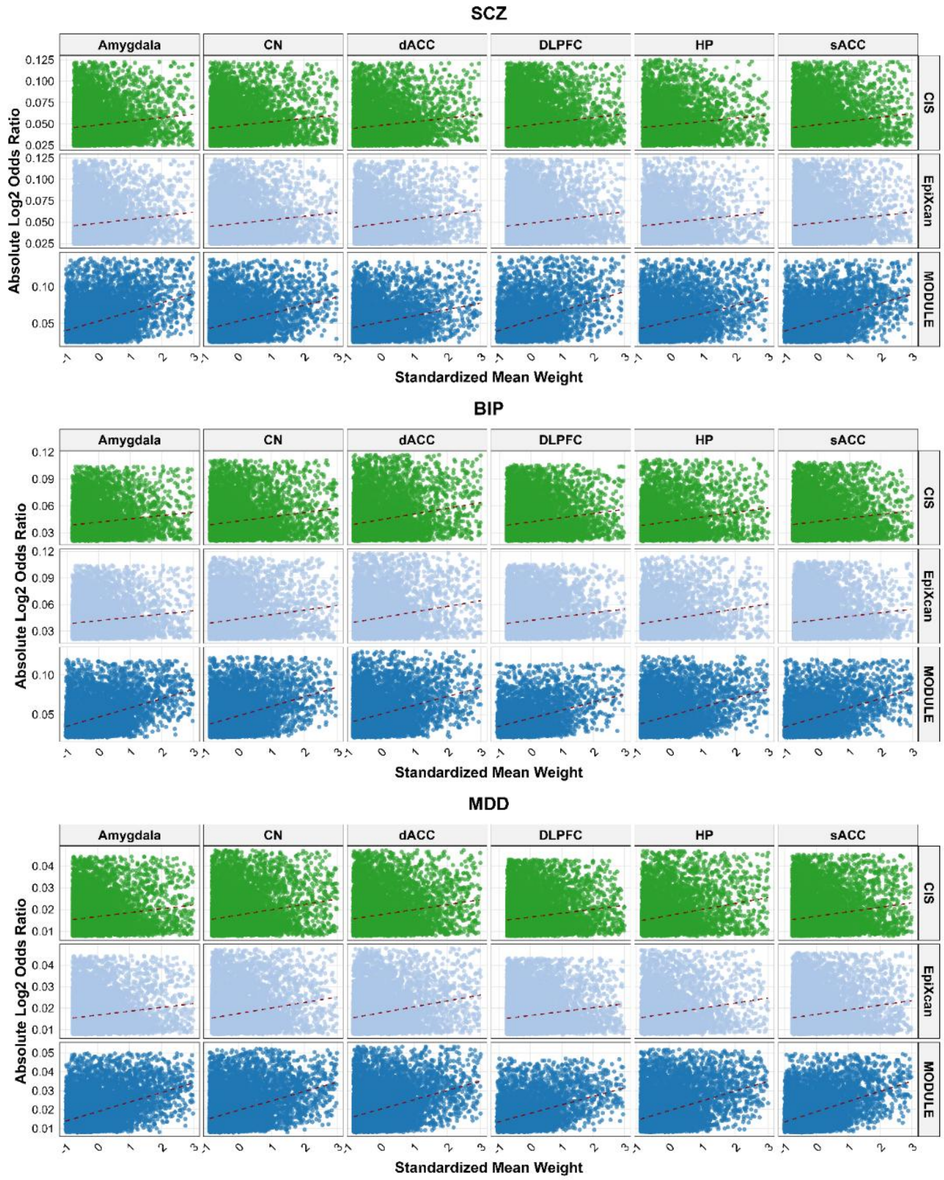

Supplementary Figure 14. Association between PGC3 SNP effect sizes and predictive model weights across brain regions. *Scatterplots show the relationship between SNP effect sizes from PGC3 GWAS summary statistics and corresponding predictive model weights for schizophrenia (SCZ), bipolar disorder (BIP), and major depressive disorder (MDD). Only SNPs with PGC3 association $p < 0.05$ were included (n = number of SNPs per trait–region–model combination; see Supplementary Table 4). The x-axis shows the standardized mean absolute SNP weight within each predictive model (CIS, green; EpiXcan, light blue; MODULE, dark blue), and the y-axis shows the absolute $\log_2$ odds ratio from PGC3 summary statistics. Each point represents one SNP. Red dashed lines indicate fitted linear regressions for visualization of the association. Brain regions are shown across columns (amygdala, CN, dACC, DLPFC, HP, sACC).*

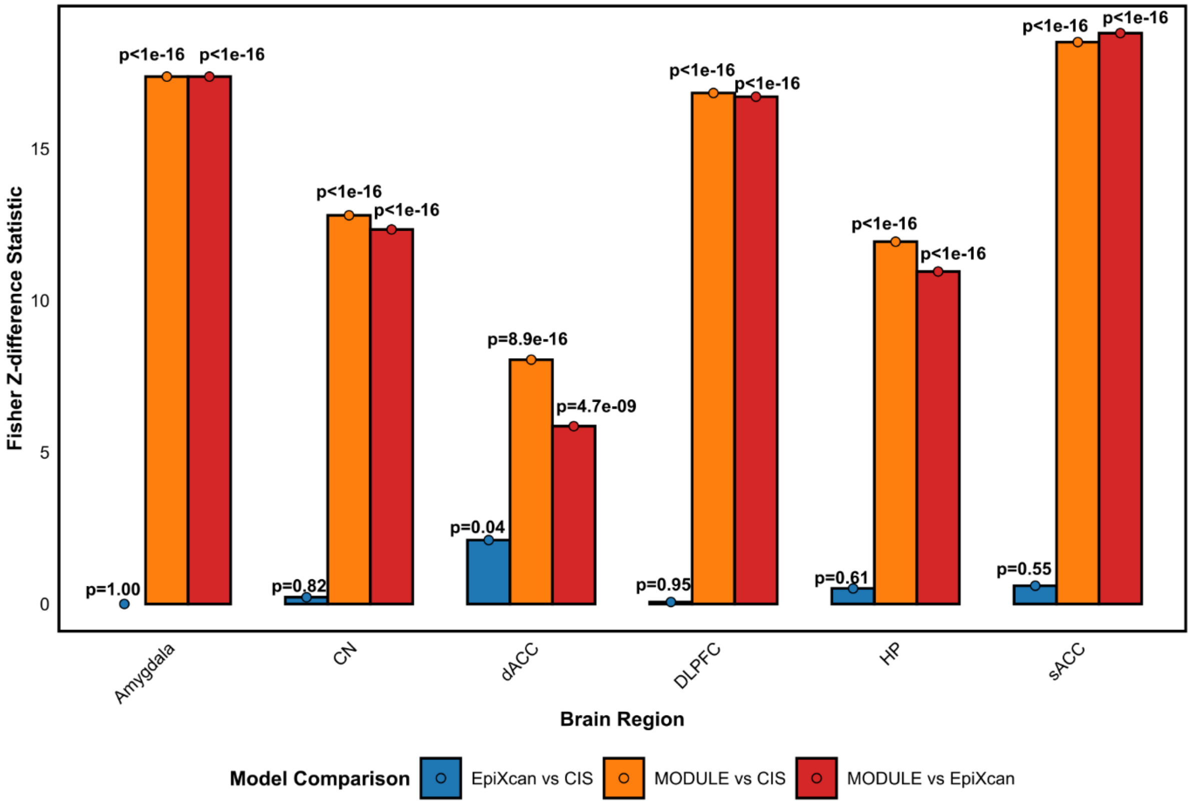

Supplementary Figure 15. Statistical comparison of model correlations with SCZ PGC3 effect sizes using Fisher's Z-transformation. *Bar plots show Fisher Z statistics comparing Pearson correlation coefficients between absolute* $\log_2$ *odds ratios from PGC3 schizophrenia GWAS and standardized mean absolute SNP weights from predictive models (CIS, EpiXcan, MODULE) across brain regions. Correlations were computed using SNPs with PGC3* $p < 0.05$ *(n = number of SNPs per region; see Supplementary Table 4). For each region, Fisher's r-to-z transformation was applied to compare pairs of correlation coefficients (EpiXcan vs CIS, MODULE vs CIS, and MODULE vs EpiXcan). Positive Z values indicate stronger correlation for the first-listed model. Statistical significance was assessed using a two-sided Fisher Z test. Exact p-values (unadjusted for multiple comparisons) are shown above each bar.*

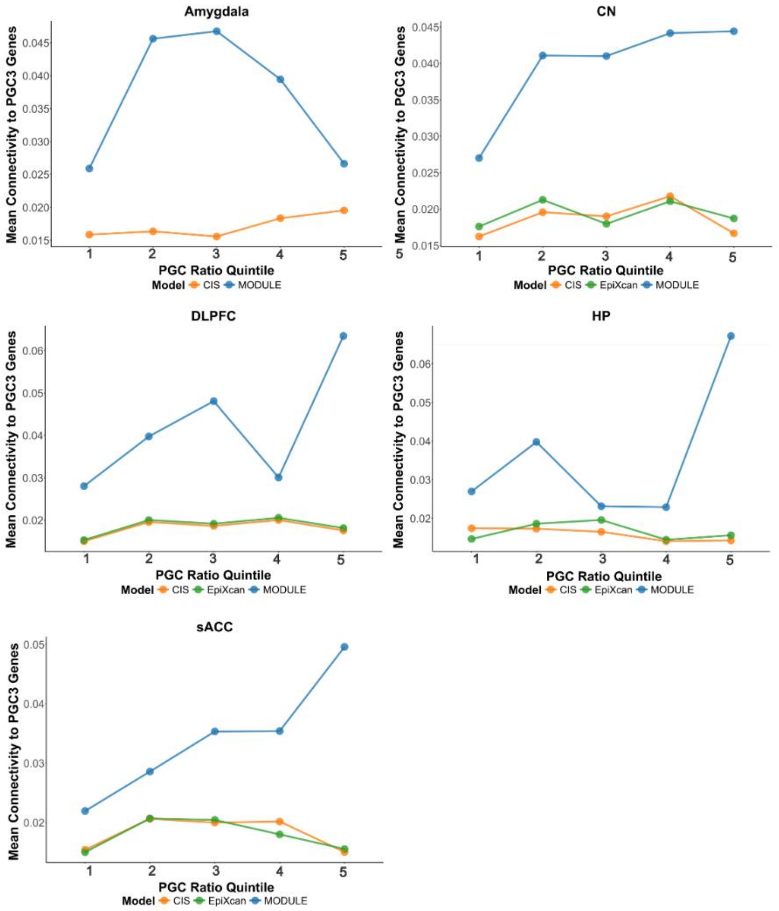

Supplementary Figure 16. Connectivity trends across PGC-weight quintiles for predicted genes. *Genes predicted by CIS, EpiXcan, and MODULE models were stratified into quintiles (1–5) based on the PGC-weight metric derived from SCZ PGC3 SNPs ($p < 0.05$). For each quintile, the mean connectivity to the 120 PGC3-prioritized genes was computed using the connectivity measure reported by Borcuk et al.[11]. The y-axis shows mean connectivity, and the x-axis shows PGC-weight quintile (1 = lowest, 5 = highest).Sample sizes (n = number of genes) per model and region are: MODULE—DLPFC (n = 5,410), Amygdala (n = 9,767), CN (n = 6,002), HP (n = 4,982), sACC (n = 10,718); CIS—DLPFC (n = 3,219), Amygdala (n = 2,129), CN (n = 2,202), HP (n = 1,617), sACC (n = 2,836); EpiXcan—DLPFC (n = 3,214), CN (n = 2,255), HP (n = 1,681), sACC (n = 2,814). Monotonic trends across ordered quintiles were assessed using (i) linear regression of mean connectivity on quintile index and (ii) Spearman rank correlation. Significance was evaluated using 1,000 gene-level permutations per model–region pair to generate empirical null distributions under no trend. Exact p-values for linear, Spearman, and permutation-based tests are reported in Supplementary Table 5.*

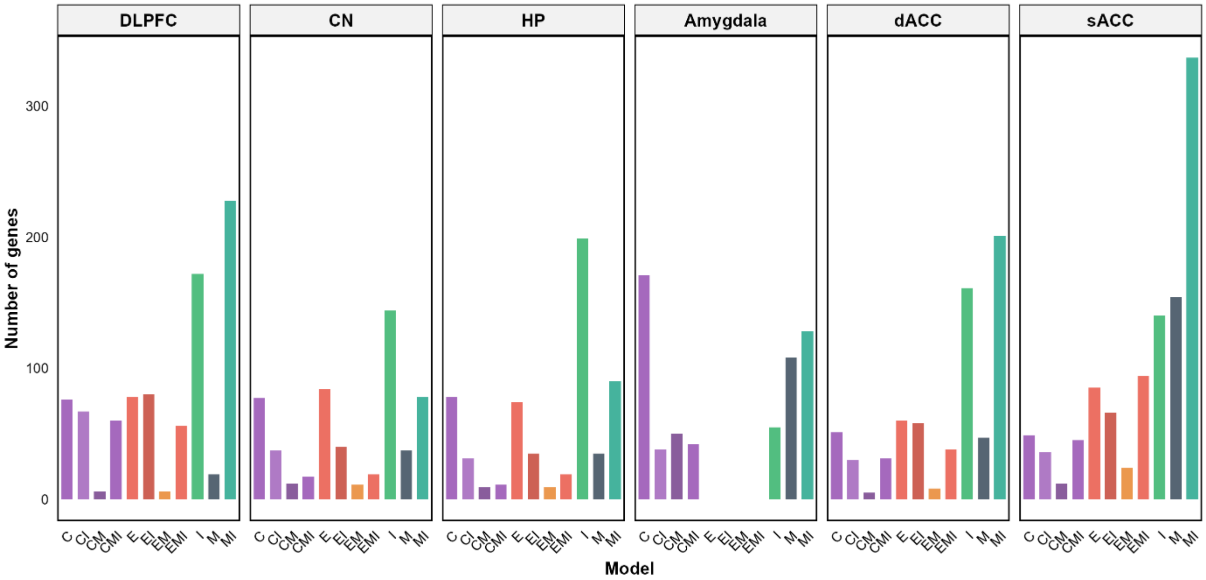

Supplementary Figure 17. Distribution of predicted genes across brain regions and predictive models in PGC3 cohorts. *predicted by each model across brain regions (DLPFC, CN, HP, amygdala, dACC, sACC) when applied across cohorts. Counts reflect all genes for which predictive models were available and generated expression predictions, irrespective of downstream association significance.Models are indicated as C (CIS), E (EpiXcan), I (INGENE), and M (MODULE). Combinations of letters denote multimodal support; for example, CMI indicates genes predicted by CIS, MODULE, and INGENE simultaneously. Bars represent the number of unique genes predicted by each individual model or multimodal combination within each brain region.*

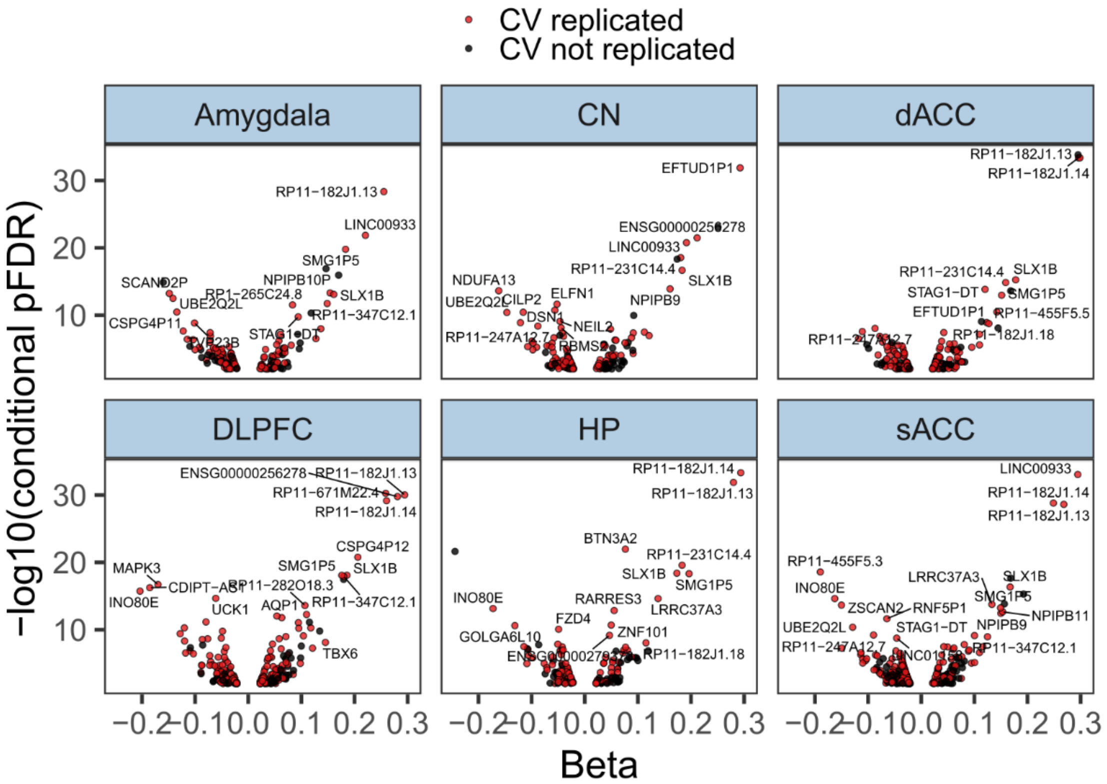

Supplementary Figure 18. Cross-validation replicability of coTWAS associations across brain regions. *Scatterplots display gene–tissue pairs identified by coTWAS in each brain region (amygdala, CN, dACC, DLPFC, HP, sACC) that passed conditional FDR ≤ 0.01. Each point represents one significant gene–tissue association (n = number of significant associations per region; see Supplementary Table 7). The x-axis shows the coTWAS effect size (β), and the y-axis shows $-\log_{10}$ of the FDR-adjusted conditional p-value. Replicability was assessed using leave-site-out validation across the 62 PGC cohorts. For each gene–tissue pair, two metrics were evaluated: (i) directional concordance, defined as the proportion of cohorts in which the leave-one-out β estimate had the same sign as the β from the held-out cohort; and (ii) the R^2 of the correlation between leave-one-out and held-out β estimates across cohorts. Statistical significance for both metrics was assessed using 10,000 permutations of held-out β values to generate null distributions. Associations were considered replicated (red points) if they exceeded the 95th percentile of the permutation-derived null (one-tailed α = 0.05) for both directional concordance and R^2; non-replicated associations are shown in black.*

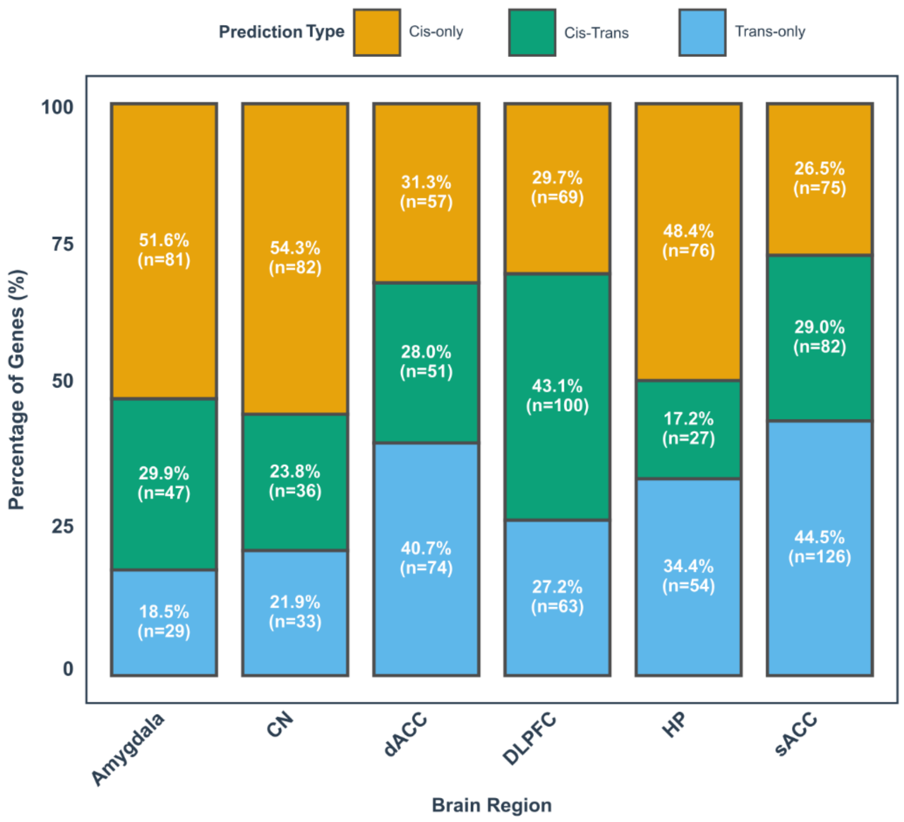

Supplementary Figure 19. Distribution of prediction types among coTWAS-significant associations. *Stacked bar plots show the distribution of coTWAS gene–tissue associations passing conditional FDR ≤ 0.01 across brain regions (amygdala, CN, dACC, DLPFC, HP, sACC). Percentages are calculated relative to the total number of significant associations within each region. Absolute counts are indicated within each bar (n = number of significant gene–tissue pairs per region). Associations are classified based on the contributing predictive component: cis-only (orange), trans-only (light blue), or cis–trans (green), indicating whether the association signal was driven exclusively by cis predictors, exclusively by trans predictors, or by a combination of both components.*

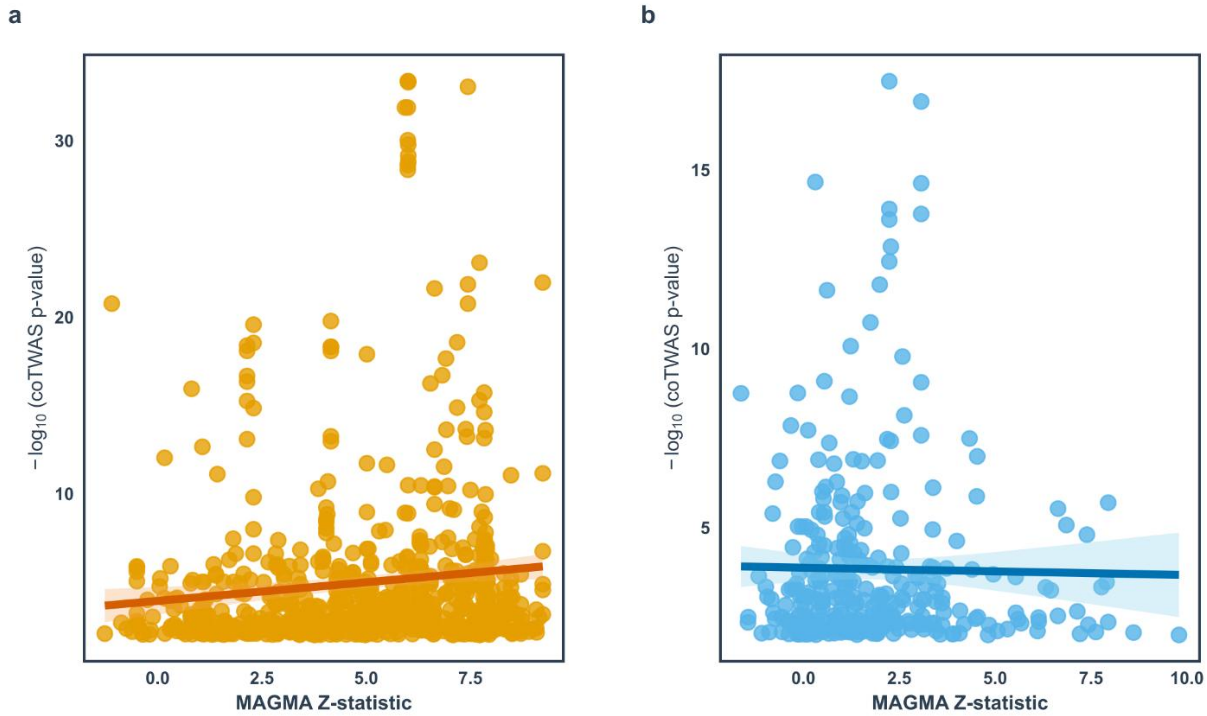

Supplementary Figure 20. Association between MAGMA gene-based statistics and coTWAS significance. *Scatterplots show the relationship between MAGMA gene-based Z-scores and coTWAS statistical significance (-log_{10} p-value) among gene–tissue associations passing conditional FDR ≤ 0.01 with available MAGMA scores (N = 1,148 genes). a) Cis-predictions (n = 796 genes). Pearson correlation between MAGMA Z-score and -log_{10}(coTWAS p-value): r = 0.108, p = 0.0034 (two-sided test). b) Trans-predictions (n = 379 genes). Pearson correlation: r = -0.016, p = 0.78 (two-sided test). Solid lines indicate linear regression fits; shaded areas represent 95% confidence intervals.*

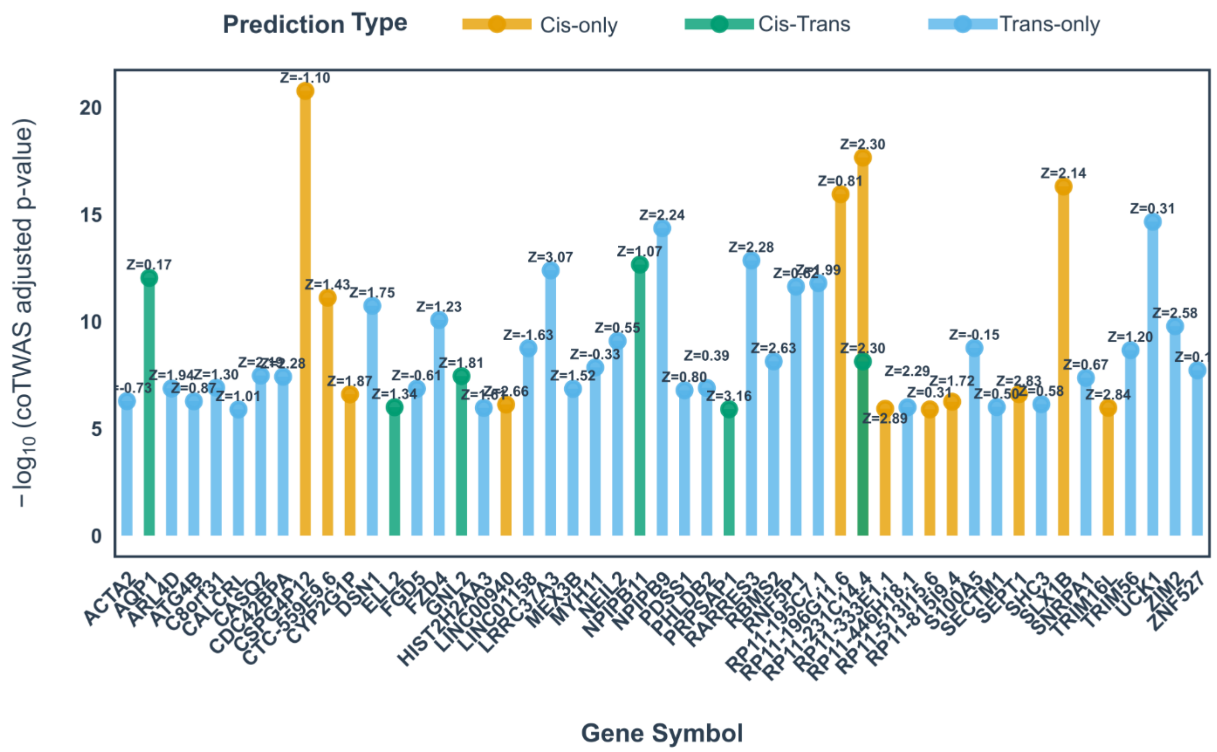

Supplementary Figure 21. Top coTWAS-prioritized genes with weak MAGMA association. *Plot showing the top 50 genes (n = 50 genes) with strong coTWAS evidence ($-\log_{10}$ FDR-adjusted p-value > 4) but weak MAGMA gene-based association ($|Z| < 4$). Genes were selected from coTWAS-significant associations (conditional FDR ≤ 0.01; see Methods) and ranked by coTWAS significance. The y-axis shows $-\log_{10}$ of the FDR-adjusted coTWAS p-value. Numbers above each point indicate the corresponding MAGMA Z-score. Points are colored by prediction type: cis-only (orange), cis–trans (green), and trans-only (light blue).*

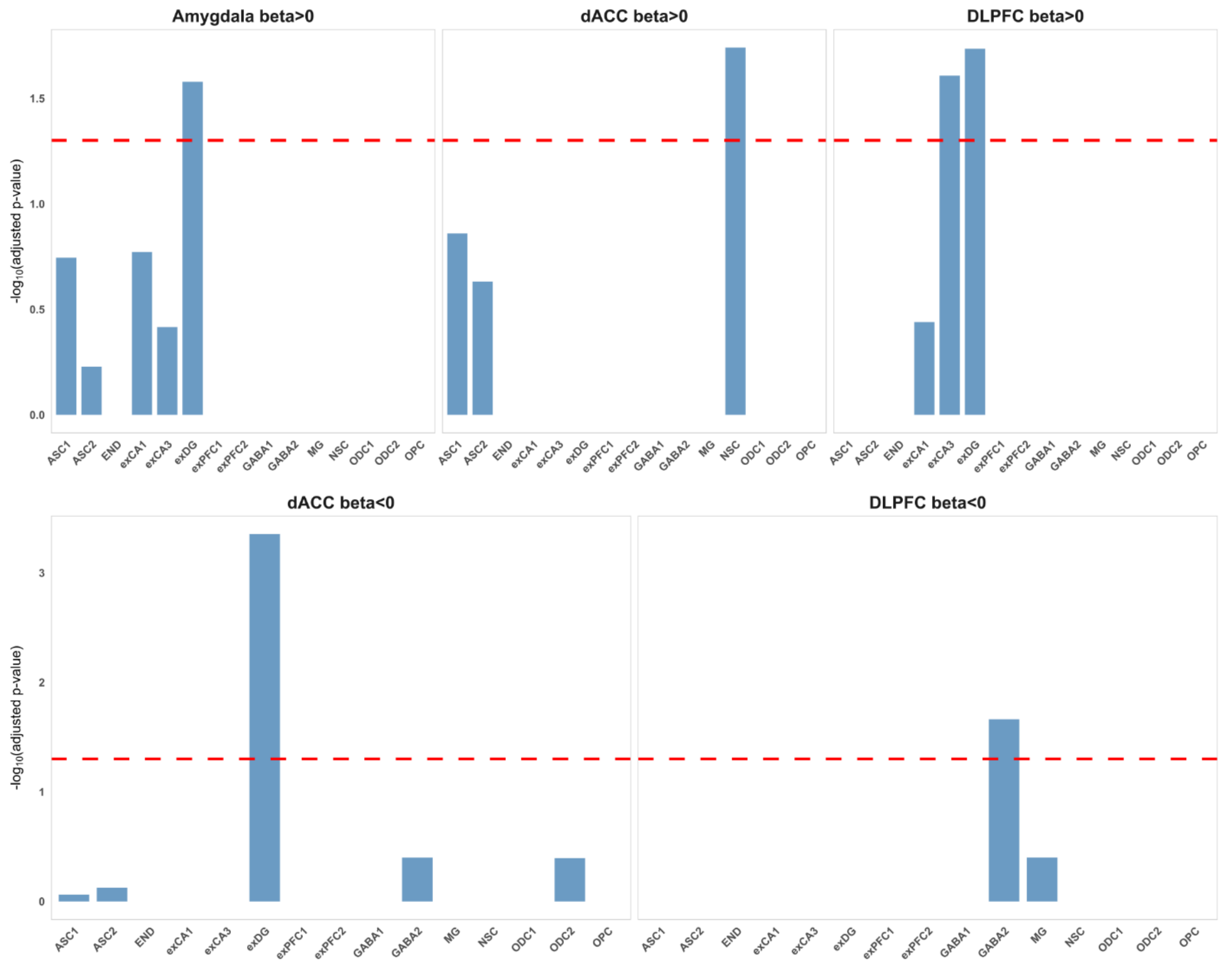

Supplementary Figure 22. Cell-type specificity of coTWAS-significant genes using the human single-cell atlas. *Cell-type enrichment analysis was performed for genes identified by coTWAS (conditional FDR ≤ 0.01) using the mean-rank gene set test implemented in the limma R package (v3.63.13). Analyses were conducted separately for genes with positive ($\beta > 0$) and negative ($\beta < 0$) coTWAS effect sizes from the SCZ logistic regression model. Bar plots show $-\log_{10}$ of FDR-adjusted enrichment p-values for each cell type within brain regions (amygdala, dACC, DLPFC). FDR correction was applied across tested cell types within each region and direction. The red dashed line indicates the significance threshold corresponding to FDR-adjusted $p = 0.05$. Top panels show enrichment results for positively associated genes ($\beta > 0$), and bottom panels show results for negatively associated genes ($\beta < 0$). n = number of coTWAS-significant genes tested per region and direction (see Table 1).*

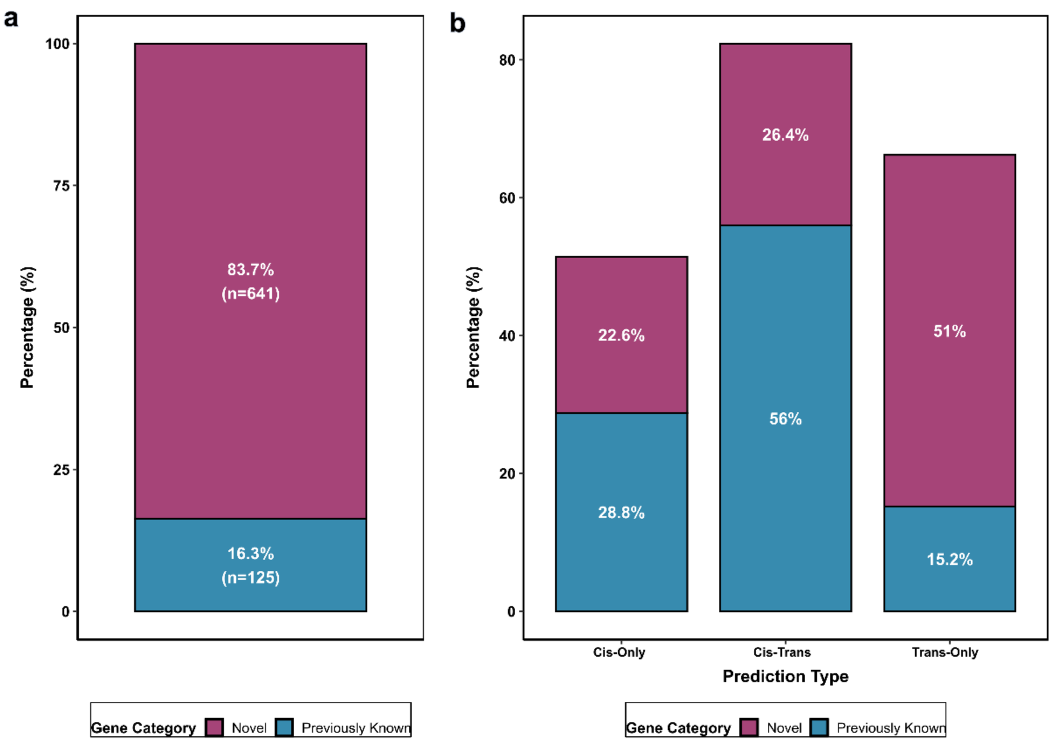

Supplementary Figure 23. Contribution of prediction type to novel vs previously reported SCZ associations. a) *Proportion of coTWAS-significant genes (conditional FDR ≤ 0.01; N = 766 genes) classified as novel (n = 641) or previously reported (n = 125) based on overlap with an externally curated list of SCZ-associated genes from published SCZ studies described in the main text (see Supplementary Figure 27). Percentages are calculated relative to the total number of significant genes.* ***b)*** *Distribution of novel and previously reported genes stratified by prediction type (cis-only, cis-trans, trans-only). Percentages are calculated within each prediction type. Previously reported genes were more frequently observed among cis-only and cis-trans predictions, whereas novel genes comprised a larger proportion of trans-only predictions.*

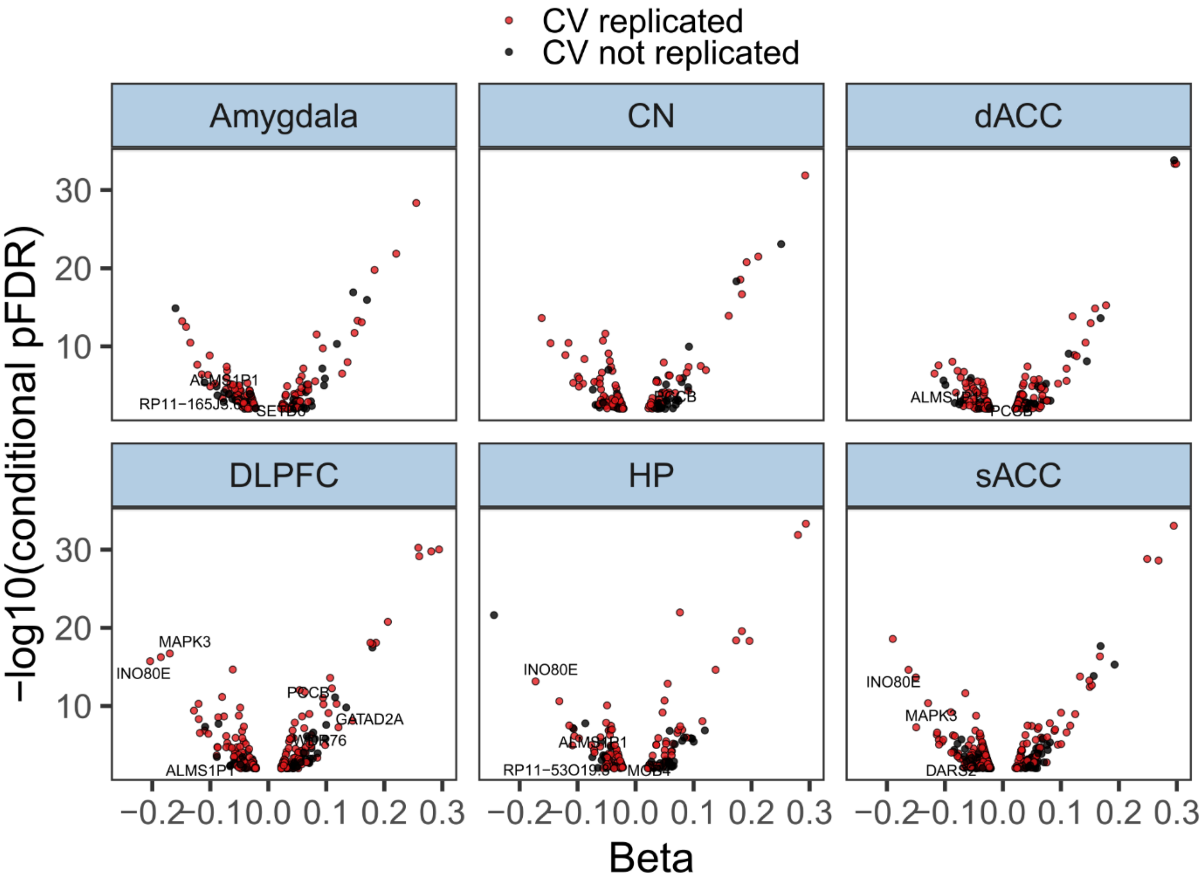

Supplementary Figure 24. Intersection between PGC3 120 prioritized genes and coTWAS significant genes across tissues*. Scatterplots display gene–tissue associations identified by coTWAS (conditional FDR ≤ 0.01) that overlap with the 120 PGC3-prioritized genes[3]. Panels are shown for each brain region (amygdala, CN, dACC, DLPFC, HP, sACC). Each point represents one significant gene-tissue pair within the intersecting gene set. The x-axis shows the coTWAS effect size (β), and the y-axis shows -log$_{10}$ of the FDR-adjusted conditional p-value. Red points indicate associations that are replicated in the leave-site-out cross-validation analysis as defined in Supplementary Figure 18; black points indicate non-replicated associations.*

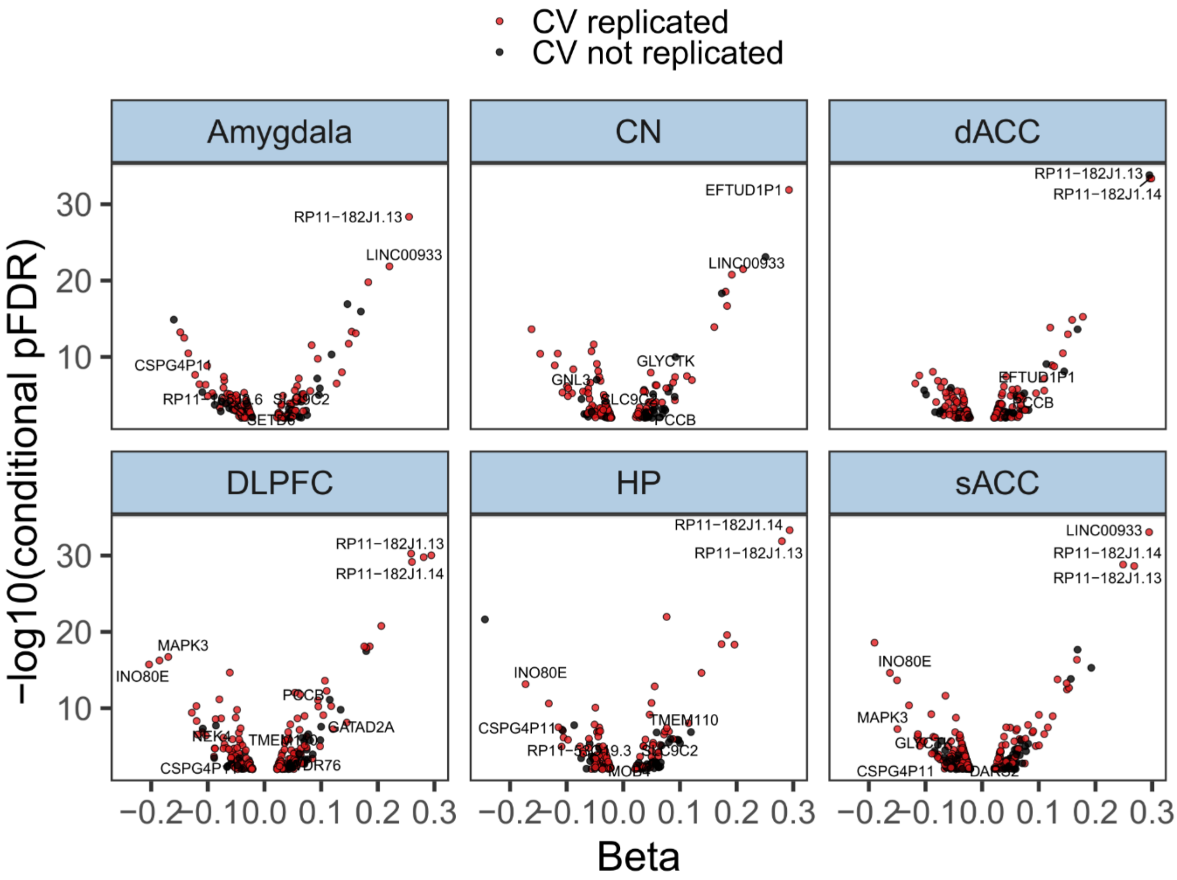

Supplementary Figure 25. Intersection between PGC3 Mendelian Randomization genes and coTWAS significant genes across tissues*. Scatterplots display gene–tissue associations identified by coTWAS (conditional FDR $\leq$ 0.01) that overlap with the MR prioritized genes[3]. Panels are shown for each brain region (amygdala, CN, dACC, DLPFC, HP, sACC). Each point represents one significant gene-tissue pair within the intersecting gene set. The x-axis shows the coTWAS effect size (β), and the y-axis shows -$\log_{10}$ of the FDR-adjusted conditional p-value. Red points indicate associations that are replicated in the leave-site-out cross-validation analysis as defined in Supplementary Figure 18; black points indicate non-replicated associations.*

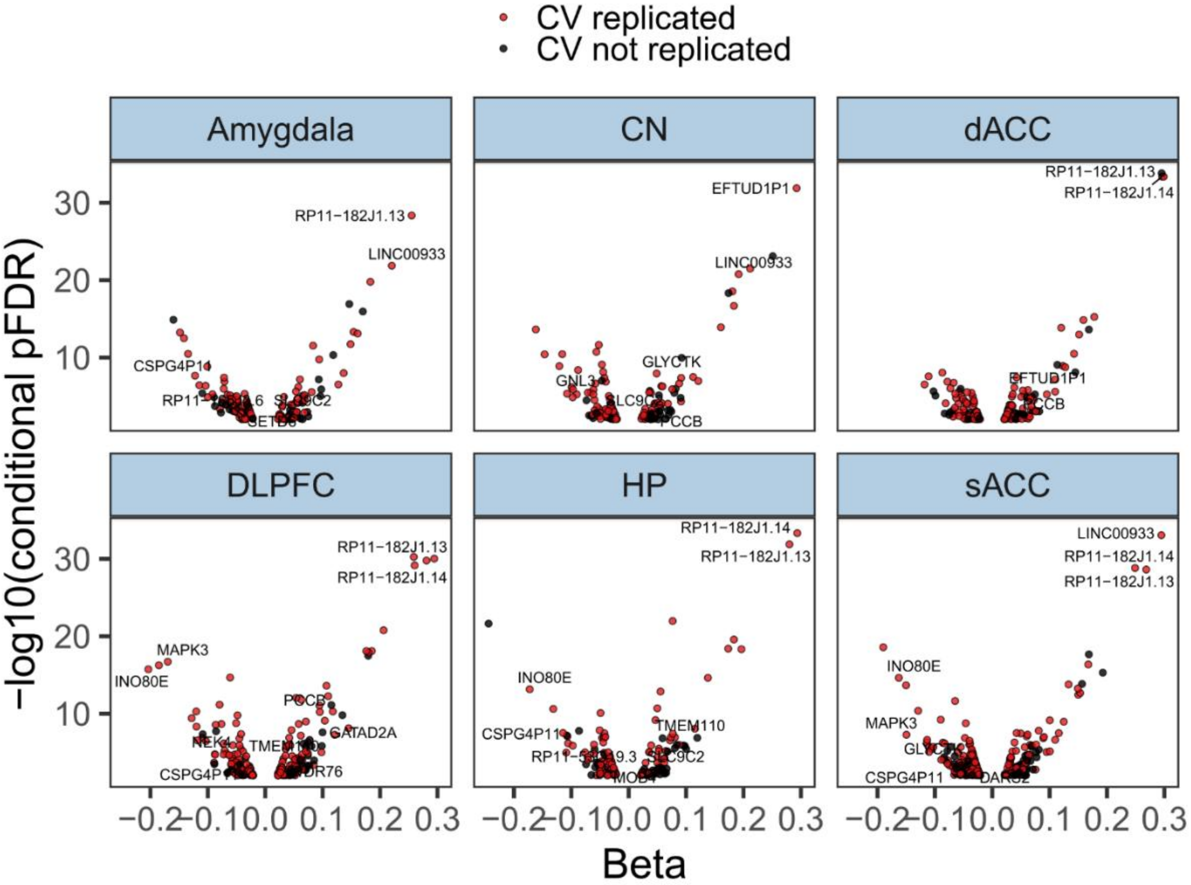

Supplementary Figure 26. Intersection between PGC3 Fine Mapping (FM) genes and coTWAS significant genes across tissues. *Scatterplots display gene–tissue associations identified by coTWAS (conditional FDR ≤ 0.01) that overlap with the FM prioritized genes[3]. Panels are shown for each brain region (amygdala, CN, dACC, DLPFC, HP, sACC). Each point represents one significant gene-tissue pair within the intersecting gene set. The x-axis shows the coTWAS effect size (β), and the y-axis shows -log$_{10}$ of the FDR-adjusted conditional p-value. Red points indicate associations that are replicated in the leave-site-out cross-validation analysis as defined in Supplementary Figure 18; black points indicate non-replicated associations as defined in Supplementary Figure 18; black points indicate non-replicated associations.*

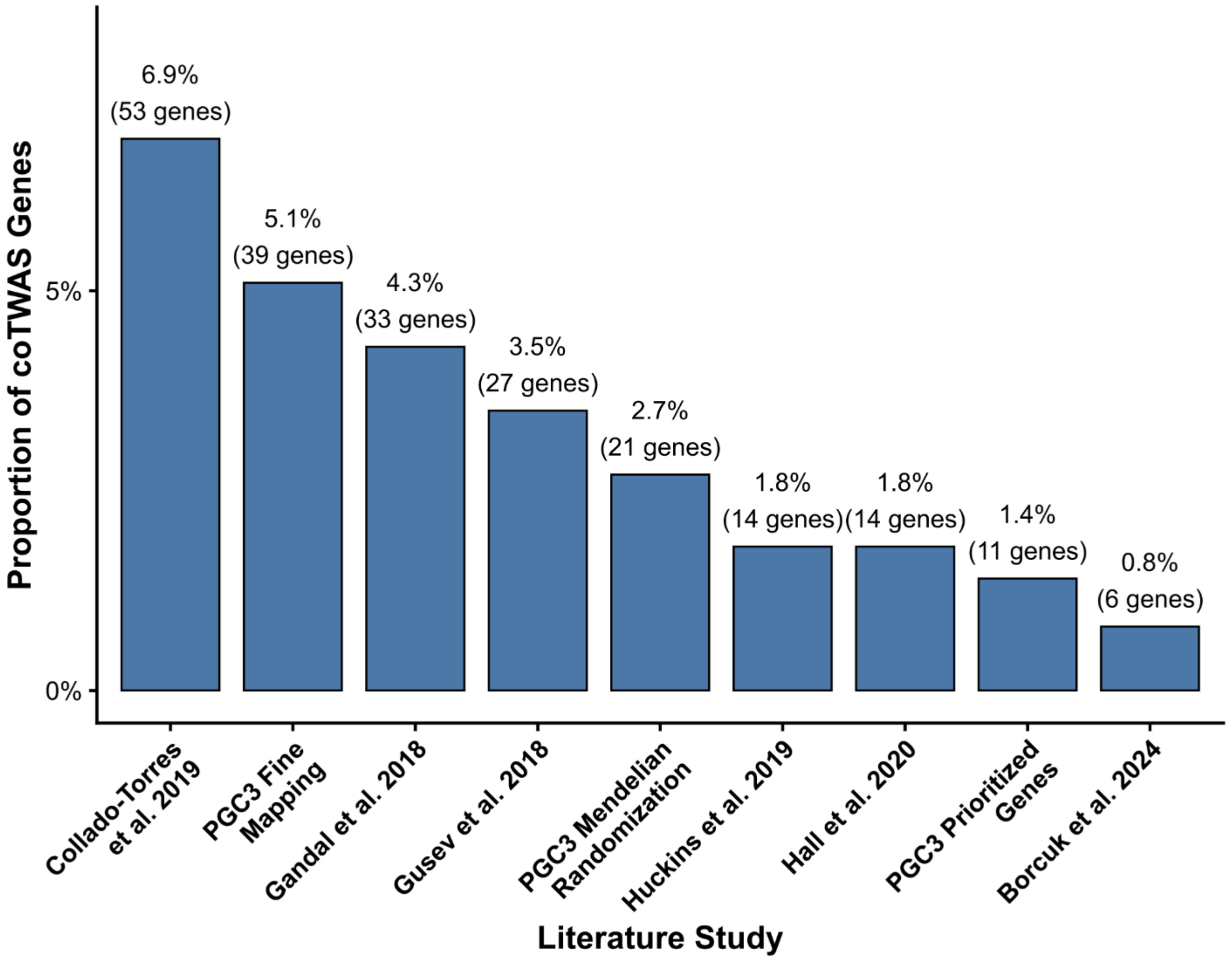

Supplementary Figure 27. Overlap of coTWAS-significant genes with previously reported SCZ-associated gene sets*. Bar plot showing the proportion of coTWAS-significant genes (conditional FDR ≤ 0.01; N = 766 genes) overlapping gene sets reported in published SCZ studies (x-axis). Percentages are calculated relative to the total number of coTWAS-significant genes, and absolute counts of overlapping genes are shown above each bar. Overlap was defined at the gene symbol level.*

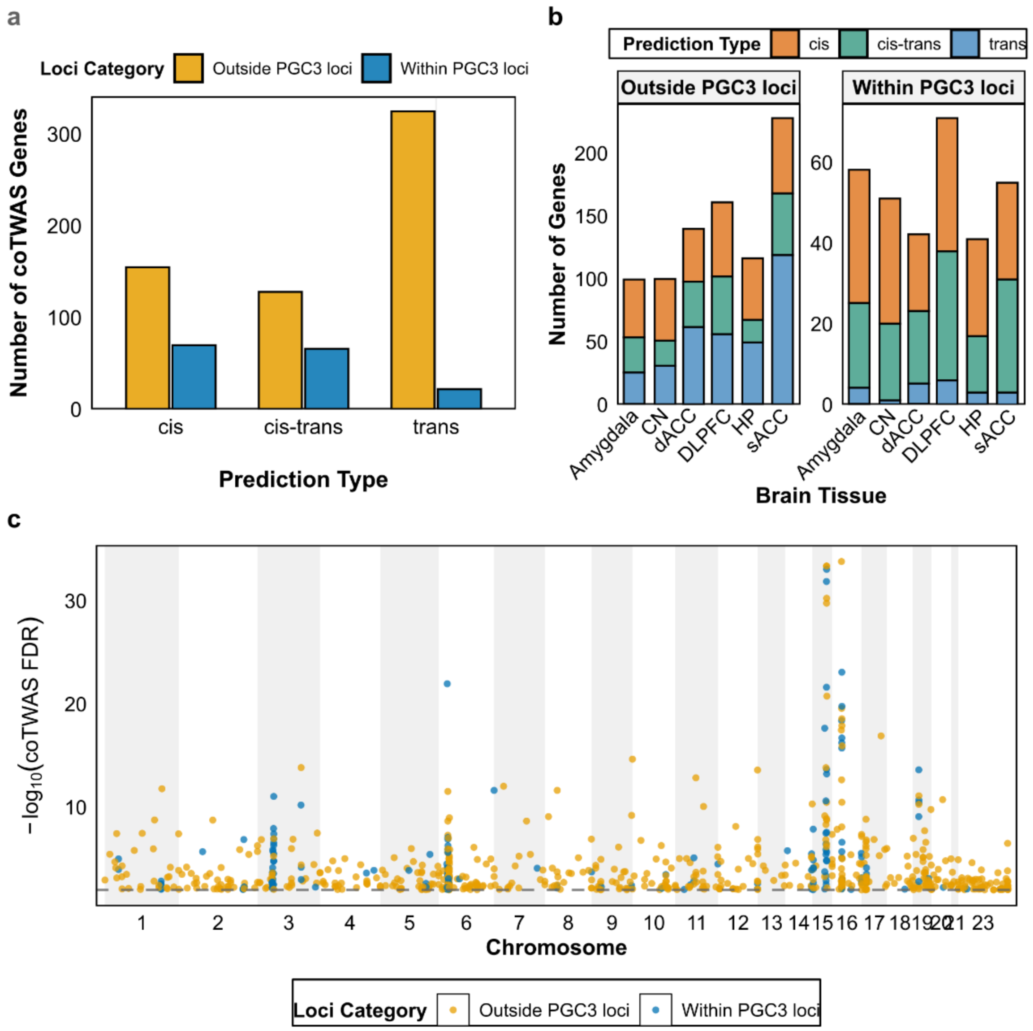

Supplementary Figure 28. Distribution of coTWAS-significant genes within and outside PGC3 schizophrenia loci. a) *Number of coTWAS-significant genes (conditional FDR ≤ 0.01; $N = 766$ genes) stratified by prediction type (cis-only, cis–trans, trans-only) and genomic location relative to genome-wide significant loci identified in the PGC3 SCZ GWAS[3]. Genes were classified as "within PGC3 loci" if their genomic coordinates overlapped defined PGC3 risk loci, and as "outside PGC3 loci" otherwise.* ***b)*** *Distribution of coTWAS-significant genes across brain tissues, shown separately for genes located outside (left panel) and within (right panel) PGC3 loci. Bars represent the number of significant genes per tissue and prediction type.* ***C)*** *Manhattan-style plot showing the chromosomal distribution of coTWAS-significant genes. The y-axis shows -$\log_{10}$ of the FDR-adjusted coTWAS p-value, and the x-axis indicates chromosomal position. Each point represents a significant gene, colored by locus category (blue, within PGC3 loci; yellow, outside PGC3 loci).*

References

1. Carithers, L.J. *et al.* A Novel Approach to High-Quality Postmortem Tissue Procurement: The GTEx Project. *Biopreserv Biobank* **13**, 311-9 (2015).
2. Fromer, M. *et al.* Gene expression elucidates functional impact of polygenic risk for schizophrenia. *Nat Neurosci* **19**, 1442-1453 (2016).
3. Trubetskoy, V. *et al.* Mapping genomic loci implicates genes and synaptic biology in schizophrenia. *Nature* **604**, 502-508 (2022).
4. Jaffe, A.E. *et al.* Developmental and genetic regulation of the human cortex transcriptome illuminate schizophrenia pathogenesis. *Nat Neurosci* **21**, 1117-1125 (2018).
5. Collado-Torres, L. *et al.* Regional Heterogeneity in Gene Expression, Regulation, and Coherence in the Frontal Cortex and Hippocampus across Development and Schizophrenia. *Neuron* **103**, 203-216.e8 (2019).
6. Jaffe, A.E. *et al.* Decoding Shared Versus Divergent Transcriptomic Signatures Across Cortico-Amygdala Circuitry in PTSD and Depressive Disorders. *Am J Psychiatry* **179**, 673-686 (2022).
7. Benjamin, K.J.M. *et al.* Analysis of the caudate nucleus transcriptome in individuals with schizophrenia highlights effects of antipsychotics and new risk genes. *Nat Neurosci* **25**, 1559-1568 (2022).
8. Zandi, P.P. *et al.* Amygdala and anterior cingulate transcriptomes from individuals with bipolar disorder reveal downregulated neuroimmune and synaptic pathways. *Nat Neurosci* **25**, 381-389 (2022).
9. Auton, A. *et al.* A global reference for human genetic variation. *Nature* **526**, 68-74 (2015).
10. Chang, C.C. *et al.* Second-generation PLINK: rising to the challenge of larger and richer datasets. *GigaScience* **4**(2015).
11. Borcuk, C. *et al.* Network-wide risk convergence in gene co-expression identifies reproducible genetic hubs of schizophrenia risk. *Neuron* (2024).
12. de Leeuw, C.A., Mooij, J.M., Heskes, T. & Posthuma, D. MAGMA: generalized gene-set analysis of GWAS data. *PLoS Comput Biol* **11**, e1004219 (2015).
13. Vabalas, A., Gowen, E., Poliakoff, E. & Casson, A.J. Machine learning algorithm validation with a limited sample size. *PLoS One* **14**, e0224365 (2019).
14. Varoquaux, G. Cross-validation failure: Small sample sizes lead to large error bars. *Neuroimage* **180**, 68-77 (2018).
15. Luningham, J.M. *et al.* Bayesian Genome-wide TWAS Method to Leverage both cis- and trans-eQTL Information through Summary Statistics. *Am J Hum Genet* **107**, 714-726 (2020).
16. Bhattacharya, A., Li, Y. & Love, M.I. MOSTWAS: Multi-Omic Strategies for Transcriptome-Wide Association Studies. *PLoS Genet* **17**, e1009398 (2021).
17. Barbeira, A.N. *et al.* Integrating predicted transcriptome from multiple tissues improves association detection. *PLoS Genet* **15**, e1007889 (2019).
18. Hartl, C.L. *et al.* Coexpression network architecture reveals the brain-wide and multiregional basis of disease susceptibility. *Nat Neurosci* **24**, 1313-1323 (2021).
19. Pergola, G. *et al.* Prefrontal Coexpression of Schizophrenia Risk Genes Is Associated With Treatment Response in Patients. *Biol Psychiatry* **86**, 45-55 (2019).
20. Pergola, G. *et al.* Consensus molecular environment of schizophrenia risk genes in coexpression networks shifting across age and brain regions. *Sci Adv* **9**, eade2812 (2023).
21. Radulescu, E. *et al.* Identification and prioritization of gene sets associated with schizophrenia risk by co-expression network analysis in human brain. *Mol Psychiatry* **25**, 791-804 (2020).
22. Gandal, M.J. *et al.* Transcriptome-wide isoform-level dysregulation in ASD, schizophrenia, and bipolar disorder. *Science* **362**(2018).

23. Gandal, M.J. *et al.* Shared molecular neuropathology across major psychiatric disorders parallels polygenic overlap. *Science* **359**, 693-697 (2018).
24. Werling, D.M. *et al.* Whole-Genome and RNA Sequencing Reveal Variation and Transcriptomic Coordination in the Developing Human Prefrontal Cortex. *Cell Rep* **31**, 107489 (2020).
25. Li, M. *et al.* Integrative functional genomic analysis of human brain development and neuropsychiatric risks. *Science* **362**(2018).
26. Walker, R.L. *et al.* Genetic Control of Expression and Splicing in Developing Human Brain Informs Disease Mechanisms. *Cell* **179**, 750-771.e22 (2019).
27. Daskalakis, N.P. *et al.* Systems biology dissection of PTSD and MDD across brain regions, cell types, and blood. *Science* **384**, eadh3707 (2024).
28. Purcell, S. *et al.* PLINK: a tool set for whole-genome association and population-based linkage analyses. *Am J Hum Genet* **81**, 559-75 (2007).
29. Chang, C.C. *et al.* Second-generation PLINK: rising to the challenge of larger and richer datasets. *Gigascience* **4**, 7 (2015).
30. Barbeira, A.N. *et al.* Exploring the phenotypic consequences of tissue specific gene expression variation inferred from GWAS summary statistics. *Nat Commun* **9**, 1825 (2018).
31. Zhang, W. *et al.* Integrative transcriptome imputation reveals tissue-specific and shared biological mechanisms mediating susceptibility to complex traits. *Nat Commun* **10**, 3834 (2019).
32. Yu, G., Wang, L.-G., Han, Y. & He, Q.-Y. clusterProfiler: an R Package for Comparing Biological Themes Among Gene Clusters. *OMICS: A Journal of Integrative Biology* **16**, 284-287 (2012).
33. Dietrich, J.P. & Leoncio, W. citation: Software Citation Tools. 0.12.2 edn (R package version 0.12.2, 2025).
34. Collado-Torres, L. *et al.* Reproducible RNA-seq analysis using recount2. *Nature Biotechnology* **35**, 319-321 (2017).
35. Kolberg, L., Raudvere, U., Kuzmin, I., Vilo, J. & Peterson, H. gprofiler2-- an R package for gene list functional enrichment analysis and namespace conversion toolset g:Profiler. *F1000Research* **9 (ELIXIR)**(2020).
36. Friedman, J.H., Hastie, T. & Tibshirani, R. Regularization Paths for Generalized Linear Models via Coordinate Descent.
37. Ritchie, M.E. *et al.* limma powers differential expression analyses for RNA-sequencing and microarray studies. *Nucleic Acids Res* **43**, e47 (2015).
38. Todorov, V. & Filzmoser, P. An Object-Oriented Framework for Robust Multivariate Analysis.
39. Maechler, M. sfsmisc: Utilities from 'Seminar fuer Statistik' ETH Zurich. 1.1-24 edn (R package version 1.1-24, 2026).
40. Dewey, M. metap: Meta-Analysis of Significance Values. 1.13 edn (R package version 1.13, 2025).
41. McKenzie, A.T. *et al.* Brain Cell Type Specific Gene Expression and Co-expression Network Architectures. *Scientific Reports* **8**, 8868 (2018).
42. McCaw, Z.R., Lane, J.M., Saxena, R., Redline, S. & Lin, X. Operating Characteristics of the Rank-Based Inverse Normal Transformation for Quantitative Trait Analysis in Genome-Wide Association Studies. *Biometrics* **76**, 1262-1272 (2019).
43. Kourentzes, N. tsutils: Time Series Exploration, Modelling and Forecasting. 0.9.4 edn (R package version 0.9.4, 2023).
44. Müller, K., Wickham, H., James, D.A. & Falcon, S. RSQLite: SQLite Interface for R. 2.4.7 edn (R package version 2.4.7, 2026).
