# Supplementary material for: Co-expression-based models improve eQTL predictions for transcriptome-wide association studies and highlight new schizophrenia-associated genes
Source: Nat Genet. 2026 Jun 22;58(7):1559–72. doi: 10.1038/s41588-026-02646-3 (PMC13364706; doi:10.1038/s41588-026-02646-3)
Supplement: Supplementary file 2 — Reporting Summary [file 41588_2026_2646_MOESM2_ESM.pdf]

nature portfolio

Corresponding author(s): Giulio Pergola

Last updated by author(s): Apr 1, 2026

Reporting Summary

Nature Portfolio wishes to improve the reproducibility of the work that we publish. This form provides structure for consistency and transparency in reporting. For further information on Nature Portfolio policies, see our Editorial Policies and the Editorial Policy Checklist.

Statistics

For all statistical analyses, confirm that the following items are present in the figure legend, table legend, main text, or Methods section.

n/a	Confirmed	
☐	☒	The exact sample size (*n*) for each experimental group/condition, given as a discrete number and unit of measurement
☐	☒	A statement on whether measurements were taken from distinct samples or whether the same sample was measured repeatedly
☐	☒	The statistical test(s) used AND whether they are one- or two-sided<br>*Only common tests should be described solely by name; describe more complex techniques in the Methods section.*
☐	☒	A description of all covariates tested
☐	☒	A description of any assumptions or corrections, such as tests of normality and adjustment for multiple comparisons
☐	☒	A full description of the statistical parameters including central tendency (e.g. means) or other basic estimates (e.g. regression coefficient) AND variation (e.g. standard deviation) or associated estimates of uncertainty (e.g. confidence intervals)
☐	☒	For null hypothesis testing, the test statistic (e.g. *F*, *t*, *r*) with confidence intervals, effect sizes, degrees of freedom and *P* value noted<br>*Give P values as exact values whenever suitable.*
☒	☐	For Bayesian analysis, information on the choice of priors and Markov chain Monte Carlo settings
☐	☒	For hierarchical and complex designs, identification of the appropriate level for tests and full reporting of outcomes
☐	☒	Estimates of effect sizes (e.g. Cohen's *d*, Pearson's *r*), indicating how they were calculated

Our web collection on statistics for biologists contains articles on many of the points above.

Software and code

Policy information about availability of computer code

Data collection	No custom software was developed for primary data collection.

Data analysis

Data analyses were conducted using publicly available software and custom scripts implemented in R (v4.5.0) and Python (v3.10.19). Genotype processing was performed using PLINK (v1.09 and v2.0). MAGMA (v1.09b) was used to map SNPs to genes and compute gene-level MAGMA Z-scores from GWAS summary statistics.

Gene-expression prediction analyses were implemented using EpiXcan (https://bitbucket.org/roussoslab/epixcan/src/master/) and custom predictive frameworks implemented in R. The CIS predictor followed the PrediXcan modelling framework (https://github.com/hakyimlab/MetaXcan), whereas the INGENE and MODULE predictors were developed in-house by modifying and extending the PrediXcan source code to incorporate trans-regulatory and network-based predictors. MetaXcan (Predict.py; https://github.com/hakyimlab/MetaXcan) was used to apply predictive models to testing genotypes. Conditional association analyses were performed using CoCo (GCTA-COJO implementation) (https://github.com/theboocock/coco).

Statistical analyses and data processing relied on R packages including glmnet (v4.1.8), limma (v3.63.13), recount (v3.22), ClusterProfiler (v3.22), gprofiler2 (v0.2.4), metap (v1.12), robustbase (v0.99.4.1), sfsmisc (v1.1.20), BRETIGEA (v1.0.3), RNOmni (v1.0.1.2), tsutils (v0.9.4) and RSQLite (v2.4.7). Additional custom utility functions were obtained from the jaffelab R repository maintained by the Lieber Institute for Brain Development (https://github.com/LieberInstitute/jaffelab). All software and packages used in this study, together with their versions and sources, are listed in the Key Resource Table in the Supplementary Information.

Custom code implementing the coTWAS analysis pipeline was developed for this study and archived at Zenodo (DOI: 10.5281/zenodo.14959416). The actively maintained version of the repository is available at https://github.com/fabiana1rossi/coTWAS.

For manuscripts utilizing custom algorithms or software that are central to the research but not yet described in published literature, software must be made available to editors and reviewers. We strongly encourage code deposition in a community repository (e.g. GitHub). See the Nature Portfolio guidelines for submitting code & software for further information.

Data

Policy information about availability of data

All manuscripts must include a data availability statement. This statement should provide the following information, where applicable:

- Accession codes, unique identifiers, or web links for publicly available datasets
- A description of any restrictions on data availability
- For clinical datasets or third party data, please ensure that the statement adheres to our policy

All data supporting the conclusions of this study are available within the article and its Supplementary Information, including the Key Resource Table.

LIBD post-mortem RNA-Seq data from the BrainSeq Phase I- III studies for CN, DLPFC, and HP are available through the database of Genotypes and Phenotypes (dbGap) and Globus collections (CN: phs003495.v1.p1, https://www.ncbi.nlm.nih.gov/projects/gap/cgi-bin/study.cgi?study_id=phs003495.v1.p1; DLPFC: jhpce#bsp2-dlpfc, http://research.libd.org/globus/jhpce_bsp2-dlpfc/index.html; HP: jhpce#bsp2-hippo, http://research.libd.org/globus/jhpce_bsp2-hippo/index.html). LIBD genotype data are available through dbGap under accession phs000979.v3.p2 (https://www.ncbi.nlm.nih.gov/projects/gap/cgi-bin/study.cgi?study_id=phs000979.v3.p2). RNA-seq data for amygdala and sACC from the BipSeq study are available through the PsychENCODE Consortium via Synapse (syn5844980) and are managed by the NIMH Repository and Genomics Resource (NRGR). Raw and processed data for DLPFC, amygdala and dACC samples from the VA PTSD study are available upon request through the PTSD Brain Bank Resource Request process (https://www.research.va.gov/programs/tissue_banking/ptsd/default.cfm).

GTEx post-mortem processed RNA-seq data used in this study are publicly available through GTEx Portal (release v8). Individual genotype data are available through controlled access via dbGaP under study accession phs000424.v8.p2. CommonMind Consoritium (CMC) MSSM-PENN-Pitt RNA-seq data were obtained upon request through Synapse (DLPFC release 3.0: syn18097439; ACC release 6.0: syn29442240). Corresponding genotype data were accessed under accession syn18097441.

PGC3 individual-level phenotype and genotype data used in this study were obtained upon request and are subject to controlled access. Access details can be found at https://pgc.unc.edu/for-researchers/data-access-committee/data-access-information/. Researchers must apply for access and comply with the data use agreement.

The GWAS summary statistics used in this study are publicly available from the PGC for SCZ, BP, and MDD through Figshare repositories. GTEx cis-eQTLs data for 59 tissues is publicly available at https://gtexportal.org/home/downloads/adult-gtex/qtl.

The study also used previously published WGCNA co-expression networks as detailed in the article, and Key Resource Table. They are also provided as Supplementary Data 1 to facilitate reproducibility.

Gene expression prediction models and analysis outputs generated in this study are available in the Zenodo repository associated with this work (DOI: 10.5281/zenodo.14959416). These include prediction models trained in the LIBD dataset (MODULE, INGENE, CIS and EpiXcan) across six brain regions, combined cis-trans prediction models validated in GTEx, and coTWAS association results including cross-validation outputs.

Research involving human participants, their data, or biological material

Policy information about studies with human participants or human data. See also policy information about sex, gender (identity/presentation), and sexual orientation and race, ethnicity and racism.

Reporting on sex and gender

Sex information for donors was recorded in the original source datasets (LIBD, GTEx, CommonMind Consortium, and PGC cohorts) and is reported in the corresponding cohort descriptions and in Supplementary Tables 2 and 6. In all analyses performed in this study, sex was included as a covariate and regressed out during gene expression preprocessing and statistical modelling steps to control for potential sex-related effects on gene expression. Therefore, the results reported here reflect associations estimated in combined samples of male and female donors after adjustment for sex effects.

Reporting on race, ethnicity, or other socially relevant groupings	Race and ethnicity were not analysed as variables in this study. All study participants included in this work were of European genetic ancestry (Table 2 and Supplementary Table 6). Genetic ancestry was determined through principal component analysis of genotype data using a cross-validation algorithm for a lasso and elastic-net regularized linear model with HapMap3 as a reference panel. Eigenvalues were computed and superpopulations were assigned based on the overlap of principal components between the study samples and the reference panel. Further details are provided in the Methods section. The restriction to individuals of European ancestry was applied to minimize potential confounding due to population stratification in genetic analyses.
Population characteristics	The study analysed previously collected post-mortem and genotype datasets from the LIBD, GTEx, CommonMind Consortium (CMC), and Psychiatric Genomics Consortium (PGC) resources. Participants included neurotypical controls and individuals diagnosed with schizophrenia, bipolar disorder, or major depressive disorder in the LIBD and CMC cohorts, while the GTEx cohort consisted of neurotypical donors. The PGC dataset included individuals with schizophrenia and controls. All individuals included in this work were of European genetic ancestry (Table 2 and Supplementary Table 6). Age and sex distributions for each cohort are reported in Table 2. Sex was included as a covariate in the analyses.
Recruitment	Participants were recruited and samples collected as part of the original LIBD, GTEx, CommonMind Consortium, and PGC studies. Detailed recruitment procedures, inclusion criteria, and ethical approvals are described in the respective primary publications and cohort documentation. The present study analysed previously generated genotype and RNA-sequencing datasets and did not involve new participant recruitment.
Ethics oversight	The research described herein complies with all relevant ethical regulations. Postmortem human brain tissues from the Lieber Institute for Brain Development (LIBD) collection were obtained with informed consent from the legal next of kin through the Offices of the Chief Medical Examiner of the District of Columbia and the Commonwealth of Virginia, Northern District, under protocol 90-M-0142 approved by the National Institute of Mental Health (NIMH)/National Institutes of Health (NIH) Institutional Review Board. Additional postmortem brain tissues were provided by the National Institute of Child Health and Human Development Brain and Tissue Bank for Developmental Disorders and by the Office of the Chief Medical Examiner for the State of Maryland with appropriate institutional approvals.<br><br>GTEx samples were collected with authorization from next of kin or legally authorized representatives under protocols approved by the respective Biospecimen Source Site Institutional Review Boards, as described in the GTEx pilot study (Carithers et al., 2015).<br><br>CommonMind Consortium (CMC) brain specimens were obtained through the Mount Sinai NIH Brain and Tissue Repository, the University of Pittsburgh Brain Tissue Donation Program, and the University of Pennsylvania Brain Bank, with informed consent from next of kin and approval from the respective institutional review boards (Fromer et al., 2016).<br><br>Ethical approval for the Psychiatric Genomics Consortium (PGC) wave 3 cohorts was obtained independently at each participating study site as described in Trubetskoy et al. (2022) and detailed in the Supplementary Cohort Descriptions.

Note that full information on the approval of the study protocol must also be provided in the manuscript.

Field-specific reporting

Please select the one below that is the best fit for your research. If you are not sure, read the appropriate sections before making your selection.

☒ Life sciences ☐ Behavioural & social sciences ☐ Ecological, evolutionary & environmental sciences

For a reference copy of the document with all sections, see nature.com/documents/nr-reporting-summary-flat.pdf

Life sciences study design

All studies must disclose on these points even when the disclosure is negative.

Sample size	No statistical methods were used to predetermine sample size. Sample sizes for the discovery and validation postmortem datasets (LIBD, GTEx, and CommonMind Consortium) were determined by the availability of brain tissue samples with both RNA-sequencing and genotype data, with sample sizes for each brain region reported in Table 2. To ensure consistency across datasets and maximize statistical power for the predictive modeling framework, analyses were restricted to individuals of European genetic ancestry aged 17 years or older, matching the characteristics of the primary replication dataset (GTEx).<br><br>Association analyses were conducted using 62 cohorts from the Psychiatric Genomics Consortium (PGC) wave 3 dataset, for which genotype data were available; sample sizes for these cohorts are reported in Supplementary Table 6. These datasets represent some of the largest publicly available resources of postmortem brain transcriptomic and genomic data and are widely used in human genetics studies.
Data exclusions	Data exclusions followed predefined quality-control procedures applied to genotype and RNA-sequencing data. For genotype datasets, variants with minor allele frequency <0.01, Hardy–Weinberg equilibrium $P < 1\times10^{-6}$, high missingness rates, or poor imputation quality were removed. Individuals with low sample call rate (<95%), heterozygosity outliers, non-matching genetic sex, or evidence of relatedness were excluded following standard RICOPILI quality-control procedures.Gene expression data underwent standard preprocessing, including filtering of low-expression genes, removal of mitochondrial genes, and outlier detection based on inter-array distance. Tissue-specific RNA-seq outlier samples deviating more than three standard deviations from the mean were excluded to ensure high-quality expression data. Additionally, individuals younger than 17 years were excluded from the LIBD discovery dataset to align the age distribution with the GTEx validation cohort.Because the analyses focused on individuals of European genetic ancestry, ancestry was inferred from genotype data and individuals not assigned to the European ancestry group were excluded.

Replication	The reproducibility of the transcriptome imputation and association pipeline was evaluated at multiple levels. First, cross-dataset replicability of trans-prediction models (INGENE and MODULE) was assessed across the LIBD, GTEx, and CommonMind Consortium datasets, retaining genes showing consistent prediction direction and significant predictive performance across datasets. Second, independent validation of all predictive frameworks (CIS, EpiXcan, INGENE, and MODULE) was performed by imputing gene expression from genotype data in external postmortem cohorts (GTEx and CMC) and comparing predicted and observed expression levels. Third, gene–trait associations were evaluated across 62 independent cohorts from the Psychiatric Genomics Consortium (PGC) wave 3 dataset using meta-analysis and leave-one-site-out validation analyses. Replication analyses confirmed consistent model performance and gene–trait associations across independent datasets and cohorts.
Randomization	Randomization was not applicable to this study because the analyses were conducted on previously generated observational datasets and did not involve experimental allocation of participants or samples to groups.
Blinding	Blinding was not applicable to this study because the analyses involved computational processing of previously generated genomic and transcriptomic datasets without investigator-driven experimental group assignment.

Reporting for specific materials, systems and methods

We require information from authors about some types of materials, experimental systems and methods used in many studies. Here, indicate whether each material, system or method listed is relevant to your study. If you are not sure if a list item applies to your research, read the appropriate section before selecting a response.

Materials & experimental systems

n/a	Involved in the study
☒	☐ Antibodies
☒	☐ Eukaryotic cell lines
☒	☐ Palaeontology and archaeology
☒	☐ Animals and other organisms
☒	☐ Clinical data
☒	☐ Dual use research of concern
☒	☐ Plants

Methods

n/a	Involved in the study
☒	☐ ChIP-seq
☒	☐ Flow cytometry
☒	☐ MRI-based neuroimaging

Plants

Seed stocks	*Report on the source of all seed stocks or other plant material used. If applicable, state the seed stock centre and catalogue number. If plant specimens were collected from the field, describe the collection location, date and sampling procedures.*
Novel plant genotypes	*Describe the methods by which all novel plant genotypes were produced. This includes those generated by transgenic approaches, gene editing, chemical/radiation-based mutagenesis and hybridization. For transgenic lines, describe the transformation method, the number of independent lines analyzed and the generation upon which experiments were performed. For gene-edited lines, describe the editor used, the endogenous sequence targeted for editing, the targeting guide RNA sequence (if applicable) and how the editor was applied.*
Authentication	*Describe any authentication procedures for each seed stock used or novel genotype generated. Describe any experiments used to assess the effect of a mutation and, where applicable, how potential secondary effects (e.g. second site T-DNA insertions, mosiacism, off-target gene editing) were examined.*
