# Supplementary material for: Co-expression-based models improve eQTL predictions for transcriptome-wide association studies and highlight new schizophrenia-associated genes
Source: Nat Genet. 2026 Jun 22;58(7):1559–72. doi: 10.1038/s41588-026-02646-3 (PMC13364706; doi:10.1038/s41588-026-02646-3)
Supplement: Supplementary file 3 — Peer Review File [file 41588_2026_2646_MOESM3_ESM.pdf]

nature portfolio

Peer Review File

Co-expression-based models improve eQTL predictions for transcriptome-wide association studies and highlight new schizophrenia-associated genes

Corresponding Author: Professor Giulio Pergola

Version 0:

Decision Letter:

9th May 2025

Dear Giulio,

Your Article "Co-expression-based models improve eQTL predictions and highlight many novel transcriptome-wide genes associated with schizophrenia" has been seen by two referees. You will see from their comments below that, while they find your work of potential interest, they have raised substantial concerns that must be addressed. In light of these comments, we cannot accept the manuscript for publication at this time, but we would be interested in considering a suitably revised version that addresses the referees' concerns.

We hope you will find the referees' comments useful as you decide how to proceed. If you wish to submit a substantially revised manuscript, please bear in mind that we will be reluctant to approach the referees again in the absence of major revisions.

To guide the scope of the revisions, the editors discuss the referee reports in detail within the team, including with the chief editor, with a view to identifying key priorities that should be addressed in revision and sometimes overruling referee requests that are deemed beyond the scope of the current study. In this case, we ask that you address all technical queries related to the co-expression-based prediction models and their downstream applications, extend the benchmarking analyses as requested to ensure fair comparisons with alternate methods, and perform additional analyses to assess validity and reproducibility. We hope you will find this prioritized set of referee points to be useful when revising your study. Please do not hesitate to get in touch if you would like to discuss these issues further.

If you choose to revise your manuscript taking into account all reviewer and editor comments, please highlight all changes in the manuscript text file. At this stage, we will need you to upload a copy of the manuscript in MS Word .docx or similar editable format.

We are committed to providing a fair and constructive peer-review process. Do not hesitate to contact us if there are specific requests from the reviewers that you believe are technically impossible or unlikely to yield a meaningful outcome.

If revising your manuscript:

*1) Include a “Response to referees” document detailing, point-by-point, how you addressed each referee comment. If no action was taken to address a point, you must provide a compelling argument. This response will be sent back to the referees along with the revised manuscript.

*2) If you have not done so already, please begin to revise your manuscript so that it conforms to our Article format instructions, available here.
Refer also to any guidelines provided in this letter.

*3) Include a revised version of any required Reporting Summary: https://www.nature.com/documents/nr-reporting-summary.pdf
It will be available to referees (and, potentially, statisticians) to aid in their evaluation if the manuscript goes back for peer review.
A revised checklist is essential for re-review of the paper.

Please be aware of our guidelines on digital image standards.

EXTENDED DATA FIGURES

When re-submitting your manuscript, please ensure that any supplementary figures and tables that are crucial to the manuscript's conclusions are converted into Extended Data figures and tables to increase visibility of these data. Extended Data figures and tables are online-only (present in the online PDF and full-text HTML versions of the paper), peer-reviewed display items that provide essential background to the article but are not included in the main article due to space constraints. A maximum of ten Extended Data display items (figures and tables) is permitted.

You may use the link below to submit your revised manuscript and related files:

Link Redacted

Note: This URL links to your confidential home page and associated information about manuscripts you may have submitted, or that you are reviewing for us. If you wish to forward this email to co-authors, please delete the link to your homepage.

If you wish to submit a suitably revised manuscript, we hope to receive it within 3-6 months. If you cannot send it within this time, please let us know. We will be happy to consider your revision so long as nothing similar has been accepted for publication at Nature Genetics or published elsewhere. Should your manuscript be substantially delayed without notifying us in advance and your article is eventually published, the received date would be that of the revised, not the original, version.

Please do not hesitate to contact me if you have any questions or would like to discuss the required revisions further.

Nature Genetics is committed to improving transparency in authorship. As part of our efforts in this direction, we are now requesting that all authors identified as 'corresponding author' on published papers create and link their Open Researcher and Contributor Identifier (ORCID) with their account on the Manuscript Tracking System (MTS), prior to acceptance. ORCID helps the scientific community achieve unambiguous attribution of all scholarly contributions. You can create and link your ORCID from the home page of the MTS by clicking on 'Modify my Springer Nature account'. For more information, please visit www.springernature.com/orcid.

Thank you for the opportunity to review your work.

Sincerely,
Kyle

Kyle Vogan, PhD
Senior Editor
Nature Genetics
https://orcid.org/0000-0001-9565-9665

Referee expertise:

Referee #1: Genetics, statistical methods, TWAS

Referee #2: Genetics, psychiatric diseases, multi-omics

Reviewers' Comments:

Reviewer #1 (Remarks to the Author):

The authors introduce two new approaches INGENE and MODULE to model additional trans-eQTL effects for predicting genetically regulated gene expression. Although there is value by leveraging both cis- and trans- eQTL effects for predicting genetically regulated gene expression, the results of this work are concerning and lack rigorous comparison with alternative methods. My major concerns are as follows:

1. Besides EpiXcan which leverages epigenetic data to improve the predictivity of genetically regulated gene expression by the standard Elastic-net model with cis-SNPs, there are a couple of other published tools that can also model trans-eQTL effects, such as MOSTWAS (PMID: 33684137) and BGW-TWAS (PMID: 32961112). Even if just compared to EpiXcan, the EpiXcan tool can be applied to all reference LIBD data of six considered brain regions, which can then be compared to the gene expression prediction models fitted by the combined/Cis-only/INGENE/MODULE. In the current paper, the authors only compare a set of pre-trained EpiXcan gene expression prediction models from the CMC reference data of the DLPFC tissue.

Even with these not-quite-comparable EpiXcan prediction models, the performance for predicting gene expression in DLPFC and other brain regions is quite good, as shown in Figure 3 and Supplementary Figure 5.

2. To leverage co-expression by INGENE and MODULE, one must first construct/estimate the co-expression gene network. Different methods would provide different co-expression networks, which will influence the findings by INGENE and MODULE. How such co-expression networks were constructed and how the uncertainty of co-expression network construction are not described nor discussed.

3. Using cis-eQTL of co-expressed genes to predict target gene expression by INGENE, and identifying co-eQTL based on their association with the eigenvalue (first principal component) of the target gene's co-expression module by MODULE, sound reasonable. However, the authors need to be clear on how such cis-eQTL and co-eQTL are determined.

4. Need to describe how MAGMA Z-score is derived, and why significant correlation is only observed for cis-predicting-eQTL, but not for trans-predicting-eQTL.

5. Previous TWAS studies (PMID: 38824563) showed that only ~30% of significant TWAS risk genes are only driven by trans-eQTL, different from the 50% reported by this study. This work detected a total of 1,764 unique TWAS risk genes, including only 249 that were previously implicated in other studies, and 1,515 unique novel findings. This is not concordant with previous findings, where most TWAS studies only detect around 10% novel findings. The findings of these 1,515 unique novel TWAS risk genes need to be justified or replicated. No gene ontology enrichment was significant for upregulated genes detected by coTWAS is concerning.

6. Because of the nature of overlapped eQTL for nearby genes, the TWAS results are known to be inflated. Significant risk genes including overlapped test eQTL are likely not independent significant signals. These need to be accounted for in reporting the number of novel findings.

7. It is shown that Mendelian Randomization and TWAS framework are aiming to test the same causal genetic effects that are mediated through gene expression (PMID: 32737316). It is expected that both analyses will obtain a large number of overlaps. So it is not true that the authors say MR is an independent method. The authors need to describe clearly how MR is conducted, and how the instrument variables (eQTL) are determined.

Reviewer #2 (Remarks to the Author):

The manuscript by Rossi et al. introduced two novel trans-eQTL models, INGENE and MODULE, designed to enhance gene expression predictions in schizophrenia (SCZ). These models leverage co-expression networks to capture trans-regulatory effects, which are hypothesized to account for a substantial portion of gene expression heritability. The study integrates these trans-models with cis-based models (CIS and EpiXcan) to improve predictive accuracy and identify novel SCZ-associated genes. It seems to be an important innovation targeting a broader regulation force of gene expression. However, several concerns exist:

1. The manuscript employs relatively loose statistical thresholds (adjusted $R^2 \geq 0.01$ and p-value < 0.05) for model/signal selection, which increases the likelihood of false positives. Why multiple test correction not considered for the gene prediction part? I understand that it is a common practice in several other existing tools, but dealing with trans-regulation, the risk of false positives may further increase with the dramatically increased number of tests. At least, at this stage, I'd love to see how many of the signals can survive multiple test correction.

2. The manuscript does not explicitly mention whether DNA genotype data were compared with RNA-seq genotype data to verify sample identity in their preprocessing. This step is critical in studies involving both genotypic and transcriptomic data to ensure that samples are correctly matched and to avoid errors in downstream analyses.

3. Regarding the CIS model's genetic variant selection, please clarify if the ±1 Mb window is defined relative to the transcription start site (TSS) or the gene body. This ambiguity could affect the interpretation of cis-eQTL boundaries and should be explicitly addressed.

4. On Page 19, the manuscript focuses on MODULE's functional enrichment of SCZ signals without adequately explaining why INGENE is not discussed.

5. When assessing replicability of the cis and trans models, the authors counted the number of genes that can be predicted in GTEx and CMC, also presented the number of genes shared by the two cohorts, and their Pearson correlation. However, they should show the percentage of sharing in the total genes for the three cohorts. My impression is that the % is not that high, meaning that there are many genes predicted only in one specific data. That could indicate the instability of prediction.

6. Since we are talking about the genetic regulators of gene expression, they should be stable across cohorts. The better test of replicability would be testing the weights/effect sizes of each gene's prediction models learned from three cohorts. If the training on different brain collections obtained consistent prediction models, I would be more convinced about their prediction. With that, they would need to train the models from three cohorts. It is a non-trivial job, but critical to prove their points about the reliable use of trans-regulation information. I would recommend that they take the time to do the right, diligent work.

Along with my 5th point above, comparing CIS, INGENE, and MODULE performance regarding their reproducibility is the most important part of this paper.

I see that they are discovering more genes, and some of them have meaningful functional implications. However, whether they are true positives needs more thoughtful validation. Having a small portion uncovered from different data is not convincing. Showing the effect sizes from different models are similar; or heritability from cis- and trans- regulations, are all not direct proof of model reproducibility.

The coTWAS identified 1,515 novel genes. Then, how do we know that they are valid findings.

7. Page 14 and Figure 3, they compared their CIS model to ExpXcan. If I understand it correctly, the only difference of these two models is the training data. Is that right? Both only used cis-eQTL. Please clarify.

8. Page 18, “40% MODULE trans-SNPs were GTEx cis-eQTLs, predicting 5,821 and 19,276 eGenes.” Quite some information was missing in this sentence. I don’t understand what they try to say. Trans-SNPs in MODULE are also cis-SNPs of those eGenes in GTEx, two brain regions?

9. If they just compare their coTWAS results to PGC3 MR gene list, which is based on cis- regions, the comparison is not meaningful. That cannot be used to prove their coTWAS based on trans- regulation, as they target very different regulations.

In summary, this is an exciting project. But I am not completely convinced about the reproducibility of their results. Additional tests and validations are required.

Reviewer #2 (Remarks on code availability):

cannot access, without permission.

Version 1:

Decision Letter:

Our ref: NG-A68300R

31st December 2025

Dear Giulio,

Your revised manuscript "Co-expression-based models improve eQTL predictions and highlight novel transcriptome-wide genes associated with schizophrenia" (NG-A68300R) has been seen by the original referees. As you will see from their comments below, they find that the paper has improved in revision, and therefore we will be happy in principle to publish your study in Nature Genetics as an Article pending final revisions to comply with our editorial and formatting guidelines.

We are now performing detailed checks on your paper, and we will send you a checklist detailing our editorial and formatting requirements soon. Please do not upload the final materials or make any revisions until you receive this additional information from us.

Thank you again for your interest in Nature Genetics. Please do not hesitate to contact me if you have any questions.

Sincerely,
Kyle

Kyle Vogan, PhD
Senior Editor
Nature Genetics
https://orcid.org/0000-0001-9565-9665

Reviewer #1 (Remarks to the Author):

All of my comments are well addressed. The extra benchmark comparison and validation analyses that the authors added in this revision make the manuscript stronger.

Reviewer #2 (Remarks to the Author):

All my concerns have been properly addressed. The paper is in excellent shape. It will be an important new tool for the community to use.

Reviewer #2 (Remarks on code availability):

I did not run all the codes, as it involves many setup and preparation. It will require significant efforts to test them. But they are well organized, documented.

Open Access This Peer Review File is licensed under a Creative Commons Attribution 4.0 International License, which permits use, sharing, adaptation, distribution and reproduction in any medium or format, as long as you give appropriate credit to the original author(s) and the source, provide a link to the Creative Commons license, and indicate if changes were made.
In cases where reviewers are anonymous, credit should be given to 'Anonymous Referee' and the source.
The images or other third party material in this Peer Review File are included in the article’s Creative Commons license, unless indicated otherwise in a credit line to the material. If material is not included in the article’s Creative Commons license and your intended use is not permitted by statutory regulation or exceeds the permitted use, you will need to obtain permission directly from the copyright holder.
To view a copy of this license, visit https://creativecommons.org/licenses/by/4.0/

Our response to the concerns raised are reported in red font below, and the corresponding revised sections in the manuscript are also in red font.

Reviewers' Comments:

Reviewer #1 (Remarks to the Author):

The authors introduce two new approaches INGENE and MODULE to model additional trans-eQTL effects for predicting genetically regulated gene expression. Although there is value by leveraging both cis- and trans- eQTL effects for predicting genetically regulated gene expression, the results of this work are concerning and lack rigorous comparison with alternative methods. My major concerns are as follows:

1. Besides EpiXcan which leverages epigenetic data to improve the predictivity of genetically regulated gene expression by the standard Elastic-net model with cis-SNPs, there are a couple of other published tools that can also model trans-eQTL effects, such as MOSTWAS (PMID: 33684137) and BGW-TWAS (PMID: 32961112). Even if just compared to EpiXcan, the EpiXcan tool can be applied to all reference LIBD data of six considered brain regions, which can then be compared to the gene expression prediction models fitted by the combined/Cis-only/INGENE/MODULE. In the current paper, the authors only compare a set of pre-trained EpiXcan gene expression prediction models from the CMC reference data of the DLPFC tissue. Even with these not-quite-comparable EpiXcan prediction models, the performance for predicting gene expression in DLPFC and other brain regions is quite good, as shown in Figure 3 and Supplementary Figure 5.

Response

We thank the reviewer for raising these important points. We concur that our data support EpiXcan as a good predictive tool, compared to other cis-eQTL techniques. We aimed to show that adding the trans-component we developed to cis-predictions increases the variance explained in gene expression, rather than conducting a comparison between cis-prediction tools.

In response to this comment, we performed two sets of new analyses: (i) we benchmarked our framework against MOSTWAS (PMID: 33684137) and BGW-TWAS (PMID: 32961112), and (ii) we retrained EpiXcan (PMID: 31444360) across all LIBD brain regions to allow its systematic integration into our pipeline. These analyses directly address the reviewer's requests.

Additionally, we retrained the INGENE model using our new EpiXcan predictions. The results strengthen the positioning of our framework relative to existing approaches. Importantly, INGENE and MODULE models have now been restricted to replicable genes, therefore the number of predicted genes has decreased.

(1) Benchmarking against MOSTWAS and BGW

We systematically compared MOSTWAS (MeTWAS, DePMA; weights downloaded from https://zenodo.org/records/4314067, and BGW-TWAS (models downloaded from https://www.synapse.org/Synapse:syn22316791/wiki/605024) to our integrated framework in the DLPFC region, the only brain region where all models are available.

Figure 1 compares the BGW-TWAS and MOSTWAS databases against our combined MODULE, INGENE, CIS, and EpiXcan (henceforth called the MICE model), trained in the LIBD dataset. For clarity, the MICE model refers to the *union of genes* predicted by each individual model.

At the training level (Fig. 1A-B), BGW predicted 14,097 genes, almost entirely through a *cis+trans* architecture; MICE predicted 21,581 unique genes, distributed as 5,128 (23.8%) *cis*, 9,816 (45.5%) *cis+trans*, and 6,637 (30.8%) *trans* predictions. Interestingly, MICE and BGW showed strong agreement in their predictions: panel B (Figure 1) highlights ~7,717 *cis+trans* genes identified by both models, representing the majority of MICE-predicted *cis+trans* genes. MOSTWAS models predicted 910 genes, mostly also captured by MICE (Figure 1B). Notably, the training cohorts differed (LIBD vs. ROS/MAP), besides the differences in methodological assumptions.

We further tested validation of predictions across independent cohorts (CMC and GTEx) (Fig. 1C–D), using the same validation thresholds we used in our study. The same trends were observed. BGW predictions overlapped considerably with MICE genes, while MICE extended coverage by ~10,000 additional replicable predictions. MOSTWAS predictions were largely encompassed within MICE. The convergence of our method with previously published benchmarks supports our current proposal, especially considering the larger number of genes predicted by MICE. We added the corresponding results and methodological details on pages 10-13 of the Supplementary Information (Supplementary Notes: Section 2.3). In addition, Figure 1 from this rebuttal has been incorporated as Supplementary Figure 7.

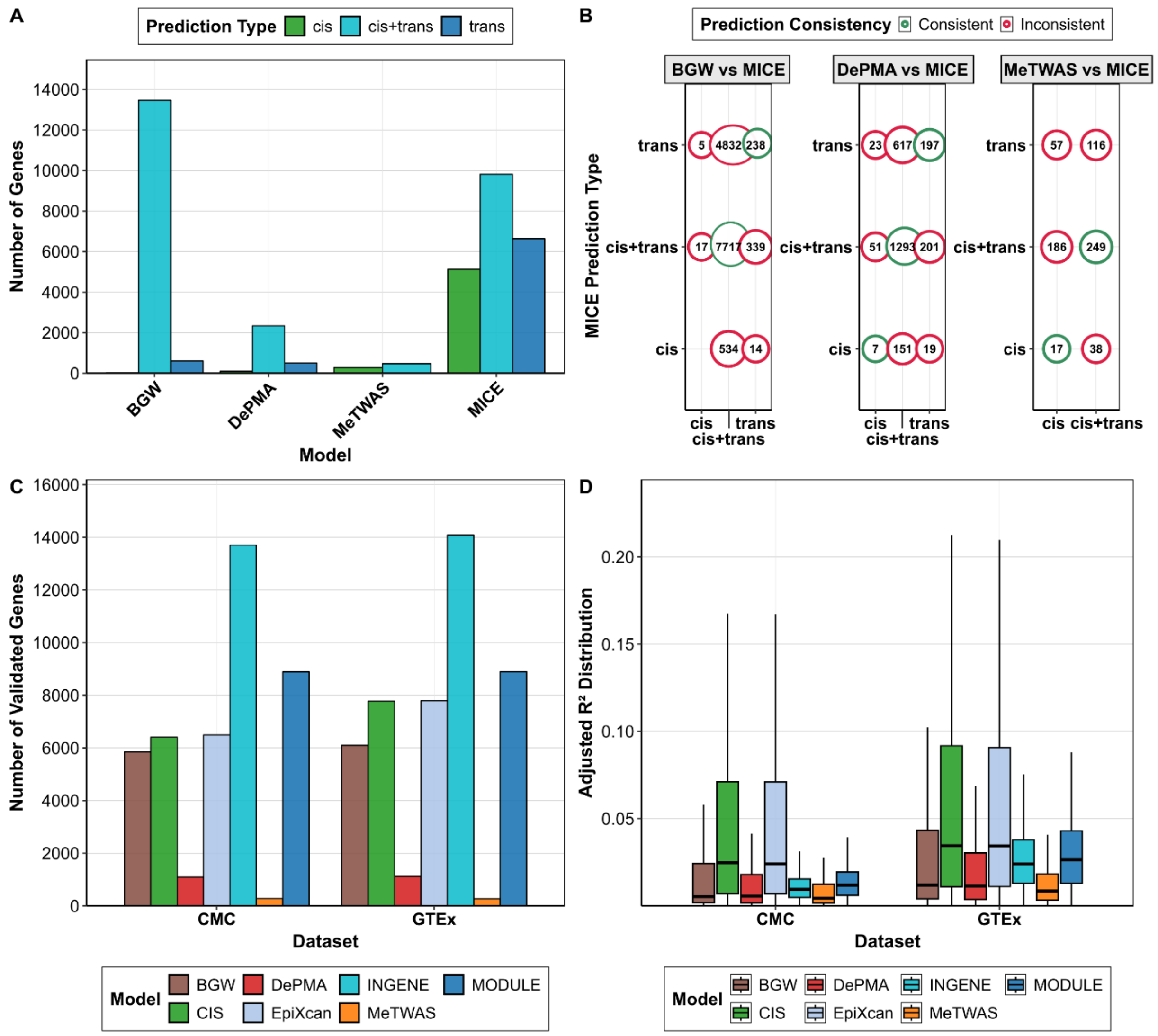

Figure 1. Comparison with MOSTWAS and BGW-TWAS models. (A) Number of genes with trained prediction models across frameworks. (B) Consistency of gene-level predictions between MICE and MOSTWAS/BGW-TWAS, measured in terms of predicted genes common across techniques. (C) Number of validated genes in independent cohorts (CMC and GTEx) across models. (D) Distribution of adjusted R^2 values for validated genes in CMC and GTEx, illustrating predictive performance across frameworks.

(2) Redesigned EpiXcan pipeline

To meet the reviewer's second request, we retrained EpiXcan across all LIBD brain regions (excluding amygdala due to missing functional annotations), obtaining fair comparisons with CIS, INGENE, and MODULE within the same training framework.

Replication of published CMC DLPFC models (Fig. 2A). We first validated that our implementation could faithfully reproduce the original EpiXcan model (PMID: 31444360) trained in CMC DLPFC. To this end, we retrieved epigenomic annotations from the Roadmap Epigenomics portal (https://egg2.wustl.edu/roadmap/web_portal). Model training was carried out using the publicly available source code from the EpiXcan Bitbucket repository (https://bitbucket.org/roussoslab/epixcan/src/master/). We downloaded CMC genotype and

RNA-seq data for the DLPFC from the same source used in the original EpiXcan study. Whenever methodological details were explicitly specified by the authors, we applied the same parameters to maximize comparability. Unspecified parameters and procedures were aligned with our pipeline to ensure internal consistency in our work (e.g., *cis*-eQTL identification).

Our retrained model predicted 10,448 unique genes, of which 10,027 achieved training significance based on adjusted R_{cv}^2. When intersecting our predictions with the 13,421 significant genes reported at the same threshold in the original EpiXcan publication (Fig. 2A), we recovered 8,074 overlapping significant genes. Quantitatively, adjusted R^2 values correlated at r = 0.74 across common genes; SNP–gene weights (N = 18,114) showed concordance at r = 0.72; and prior/penalty factor distributions for common SNPs were highly correlated (r > 0.8). Together, these results confirm published successful application of EpiXcan models developed in DLPFC, providing confidence that our downstream cross-region extensions are unbiased.

Extension across LIBD brain regions (Fig. 2D). Having confirmed reproducibility in CMC DLPFC, we then extended EpiXcan training to all available LIBD brain regions. Across CN, dACC, DLPFC, HP, and sACC, EpiXcan consistently outperformed the CIS model trained without functional annotations, as in the original publication. When we restricted the analyses to the 25,101 genes shared between CIS and EpiXcan across regions, the mean CV adjusted R^2 increased from 0.140 for CIS to 0.153 for EpiXcan, approximately a 9% relative gain in predictive accuracy. In terms of coverage, EpiXcan predicted 25,702 unique genes across regions, exceeding the 25,285 genes predicted by CIS. These results demonstrate that the benefits of EpiXcan extend robustly across multiple brain regions, not just in the originally published DLPFC setting, thereby justifying its inclusion in region-wide comparative analyses.
Based on these results, we incorporated LIBD-trained EpiXcan models into our pipeline, enabling direct region-wide comparisons with CIS, INGENE, and MODULE. The results of the EpiXcan integration have been added on pages 13-14, 16, and 18 of the manuscript. We also updated Figure 2 by adding panels A and B and included a new Supplementary Figure 1. Details of the EpiXcan training methods have been added on page 46.

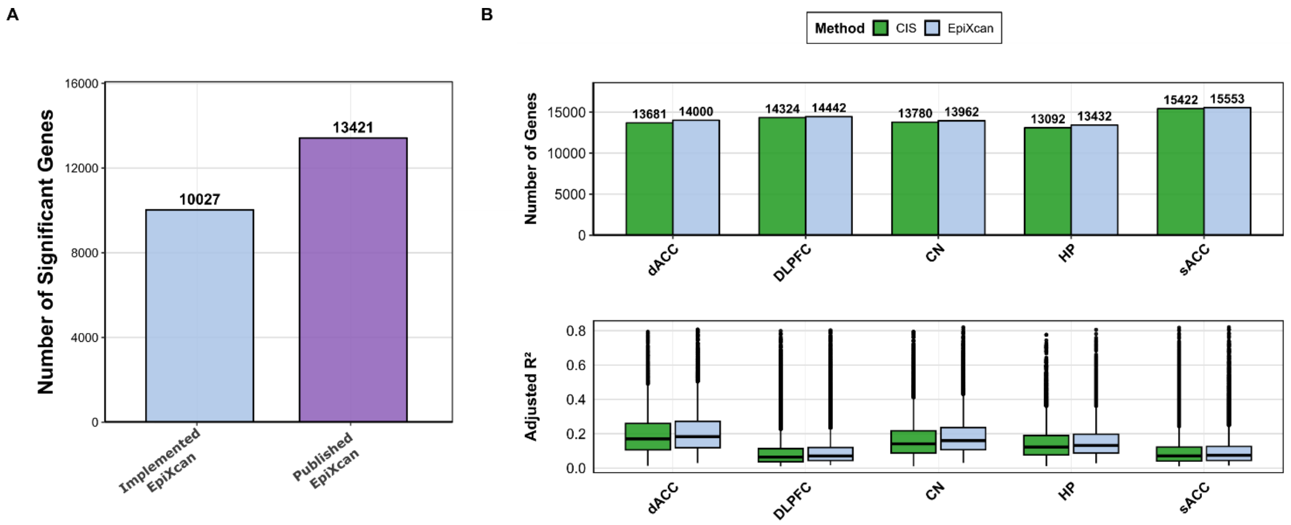

Figure 2. Redesigned EpiXcan pipeline and replication across brain regions. *(A) Number of significant genes predicted by our implemented EpiXcan compared with the published EpiXcan in the CMC DLPFC. (B) Extension of EpiXcan training to LIBD brain regions (dACC, DLPFC, CN, HP, sACC). Top: number of genes with predictive models across regions. Bottom: distribution of adjusted R^2 values, showing consistent improvement of EpiXcan over CIS.*

2. To leverage co-expression by INGENE and MODULE, one must first construct/estimate the co-expression gene network. Different methods would provide different co-expression networks, which will influence the findings by INGENE and MODULE. How such co-expression networks were constructed and how the uncertainty of co-expression network construction are not described nor discussed.

Response:

We thank the reviewer for raising this important point. We would like to clarify that we identified no new co-expression networks in this study. Instead, all 48 networks used by INGENE and MODULE were obtained from previously published resources. We thus employed existing co-expression gene sets as lists of partner genes for each gene we considered. As our methods do not use network-derived parameters, they can be readily adapted to include additional models. WGCNA modules serve to reduce the multiple testing burden and limit false positives in trans-eQTL prediction.

We cited the pertinent manuscripts in the main text and listed them in the Supplementary Key Resources table. We concur that multiple networks yield complementary perspectives on co-expression, so we minimized dataset- and method-specific biases by leveraging an ensemble of independently validated networks. Our approach follows the general principle that an ensemble of models is more effective than single models when conducting predictions.

For the sake of transparency, we now provide a supplementary Excel file (as Supplementary Data) listing, for each network used in our analyses, the associated genes.

3. Using cis-eQTL of co-expressed genes to predict target gene expression by INGENE and identifying co-eQTL based on their association with the eigenvalue (first principal

component) of the target gene's co-expression module by MODULE, sound reasonable. However, the authors need to be clear on how such cis-eQTL and co-eQTL are determined.

Response:

We agree that further clarification was needed on how cis-eQTLs are defined. In the revised manuscript, we have expanded the Methods to describe the identification of cis-eQTLs used in CIS and EpiXcan (p. 45, "cis-eQTL discovery"). Briefly, cis-eQTLs were defined as SNPs within ±1 Mb of each gene's transcription start site (TSS) (5′ end of the gene, strand-specific). Associations with expression were tested using 4-fold CV with robust linear models, and significance was assessed with F-tests. Results across folds were meta-analyzed to obtain a single effect size, standard error, and p-value per SNP–gene pair. SNPs were then ranked by p-value, and LD pruning ($R^2 < 0.1$ within ±250 kb) was applied to retain independent signals.

For MODULE, a detailed description of co-eQTL discovery was already provided in the original manuscript (p. 48). Briefly, SNPs were mapped to genes within each co-expression module and tested for association with the module eigengene (PC1) in a 4-fold CV framework using robust linear models. SNPs were ranked across folds, pruned for LD, and the top 5% of cross-validated associations were retained as co-eQTLs.

Finally, we would like to emphasize that the INGENE model does not use cis-SNPs of the target gene directly. Instead, INGENE relies on the cis-predicted expression of co-expressed partner genes (obtained from CIS/EpiXcan models) as predictors of the target gene. The methodological procedure is described in the sections *INGENE Models and Assumptions* and *INGENE Training and Application to Testing Dataset* (p. 46-47).

4. Need to describe how MAGMA Z-score is derived, and why significant correlation is only observed for cis-predicting-eQTL, but not for trans-predicting-eQTL.

Response:

We thank the reviewer for this comment and are happy to clarify. In our study, gene-level MAGMA Z-scores were derived following the revised MAGMA gene analysis framework (PMID:33372276). Specifically, for each gene we computed a *Z*-score of association with SCZ based on GWAS effect size (PMID: 35396580) of SNPs in the genomic loci. We have added this information to the Supplementary Methods section 1.7 (page 7).

Regarding the observed correlations, the finding that cis-predicting models show significant correlation with MAGMA Z-scores, while trans-predicting models do not, is fully consistent with the cis-nature of MAGMA inferences. As such, MAGMA is well-suited to capture the same signal modeled by cis-based predictors, but it does not reflect the distal, network-level contributions that underlie INGENE and MODULE. Considering that we explicitly excluded the cis region from the trans-prediction of each gene, the absence of correlation between MAGMA Z-scores and our trans-predicting models is expected and reassuring. We have now clarified this point in the revised Results on page 27.

5. Previous TWAS studies (PMID: 38824563) showed that only ~30% of significant TWAS risk genes are only driven by trans-eQTL, different from the 50% reported by this study. This work detected a total of 1,764 unique TWAS risk genes, including only 249 that were previously implicated in other studies, and 1,515 unique novel findings. This is not concordant with previous findings, where most TWAS studies only detect around 10% novel findings. The findings of these 1,515 unique novel TWAS risk genes need to be justified or replicated. No gene ontology enrichment was significant for upregulated genes detected by coTWAS is concerning.

Response:

We thank the reviewer for highlighting this issue and understand that it required clarification. In our revised framework, which integrates cross-trained prediction models (see response to Reviewer #2, point 6) and conditional analysis (see following request), we recomputed coTWAS and examined the distribution of cis- and trans-only associations. As shown in Fig. 3 (Fig. S19 in the revised manuscript), the updated analysis substantially restricted the overall fraction of trans-only discoveries to the most reliable ones also revealing regional variability (from ~19% in Amygdala to ~45% in sACC). These figures are consistent with the 30% estimate mentioned by the Reviewer.

Importantly, the study cited by the Reviewer (PMID: 38824563) applied BGW-TWAS models. When we re-analyzed the DLPFC, the same brain region used in that study, we obtained a trans-only proportion of 27.2% (Figure 3), very similar to the BGW-TWAS result reported in the study. The higher overall average in our study arises from including additional brain regions (e.g., sACC), some of which show stronger trans contributions, rather than from inflation derived from our methods.

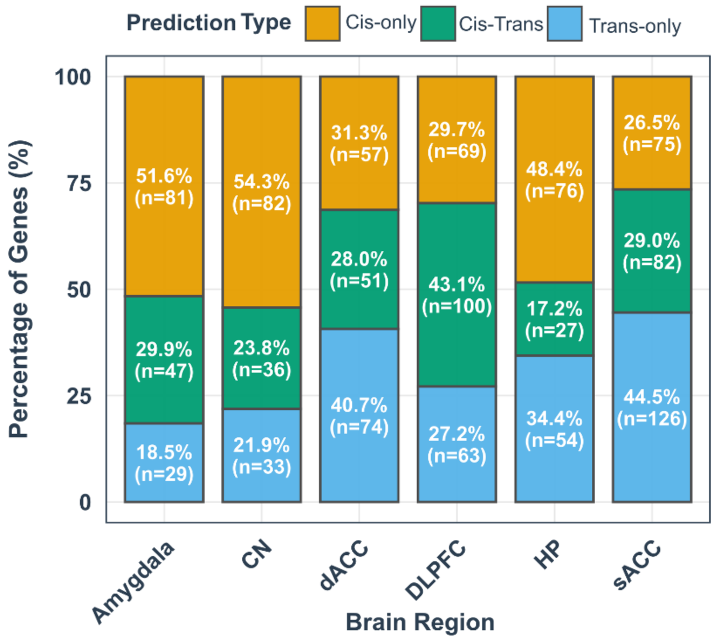

Figure 3. Distribution of cis-only, cis+trans, and trans-only associations across brain regions. *Stacked bar plots show the proportion of coTWAS discoveries attributable to cis-only, cis+trans, and trans-only models in six brain regions (amygdala, CN, dACC, DLPFC, HP, sACC). Trans-only associations account for ~21% of discoveries in CN and up to ~45% in sACC, revealing substantial regional variability. In the DLPFC, the trans-only proportion is 29.6%, closely matching the value reported in BGW-TWAS (PMID: 38824563).*

Regarding the concern about the higher proportion of novel discoveries compared to previous TWAS studies, we note that earlier large-scale efforts relied almost exclusively on

cis-based prediction models. By contrast, our framework explicitly incorporates trans-eQTL information, which broadens the search space and increases the likelihood of identifying associations that cannot be retrieved based on cis-methods.

Our revised manuscript reports the results of a Locus-independence Conditional Analysis, which we now adopt as our main framework for identifying independent associations (see response to Reviewer #1, point 6). Additionally, we explored replicability using a leave-one-out CV across the 62 PGC3 sites (see response to Reviewer #1, point 6 for details). As expected, the conditional analysis yielded fewer absolute discoveries than the classical coTWAS meta-analysis (Fig. 4A). The overall proportion of novel versus previously reported genes remained stable (~77–84% novel). To address the Reviewer's concern, we stratified results by prediction type (cis-only, cis+trans, trans-only; Fig. 4B). We found that most previously reported genes were rediscovered through cis-only and cis+trans models, consistent with prior TWAS studies and with the expectation of this Reviewer in quantitative terms. In contrast, novel associations were strongly enriched in the trans-only category, underscoring that distal regulation is the major contributor to new discoveries in our framework.

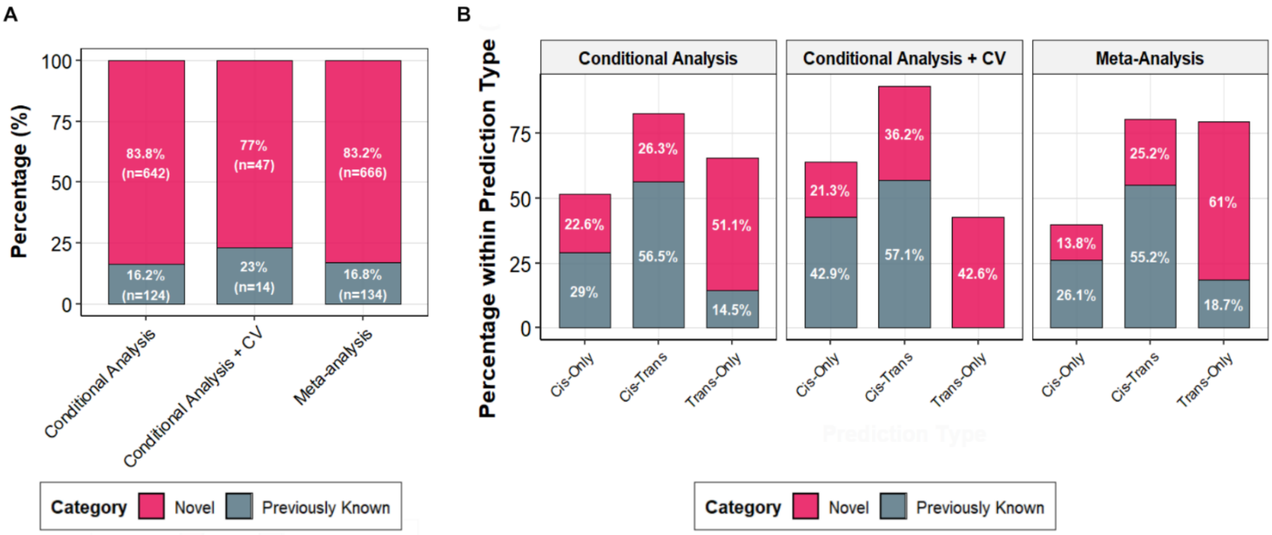

Figure 4. ***Contribution of prediction type to novel vs previously reported SCZ associations.*** *(A) Proportion of novel and previously known genes identified under three frameworks: locus-independence conditional analysis, conditional analysis with leave-one-out CV, and standard coTWAS meta-analysis. Across approaches, ~77–84% of discoveries are novel. (B) Stratification of discoveries by prediction type (cis-only, cis+trans, trans-only). Previously known genes were largely recovered through cis-only and cis+trans models, whereas novel associations were strongly enriched in the trans-only category, highlighting distal regulation as the primary source of new signals.*

To further address this concern, we performed an additional analysis evaluating the overlap between coTWAS-significant genes and genome-wide significant PGC3 loci (PMID: 35396580). As shown in Figure 5 (added as new Supplementary Figure S28 in the manuscript), 158 genes (27.4% of all tissue-specific associations) mapped within PGC3 regions, while 608 genes (72.6%) lay outside. Notably, cis and cis–trans predictions accounted for most within-locus associations, whereas trans-only signals were strongly enriched outside established GWAS intervals, a pattern consistent across all examined brain regions (Figure 5A–B). The chromosomal distribution (Supplementary Figure 5C) further revealed that several of the most significant associations (–log□□ FDR > 20) clustered

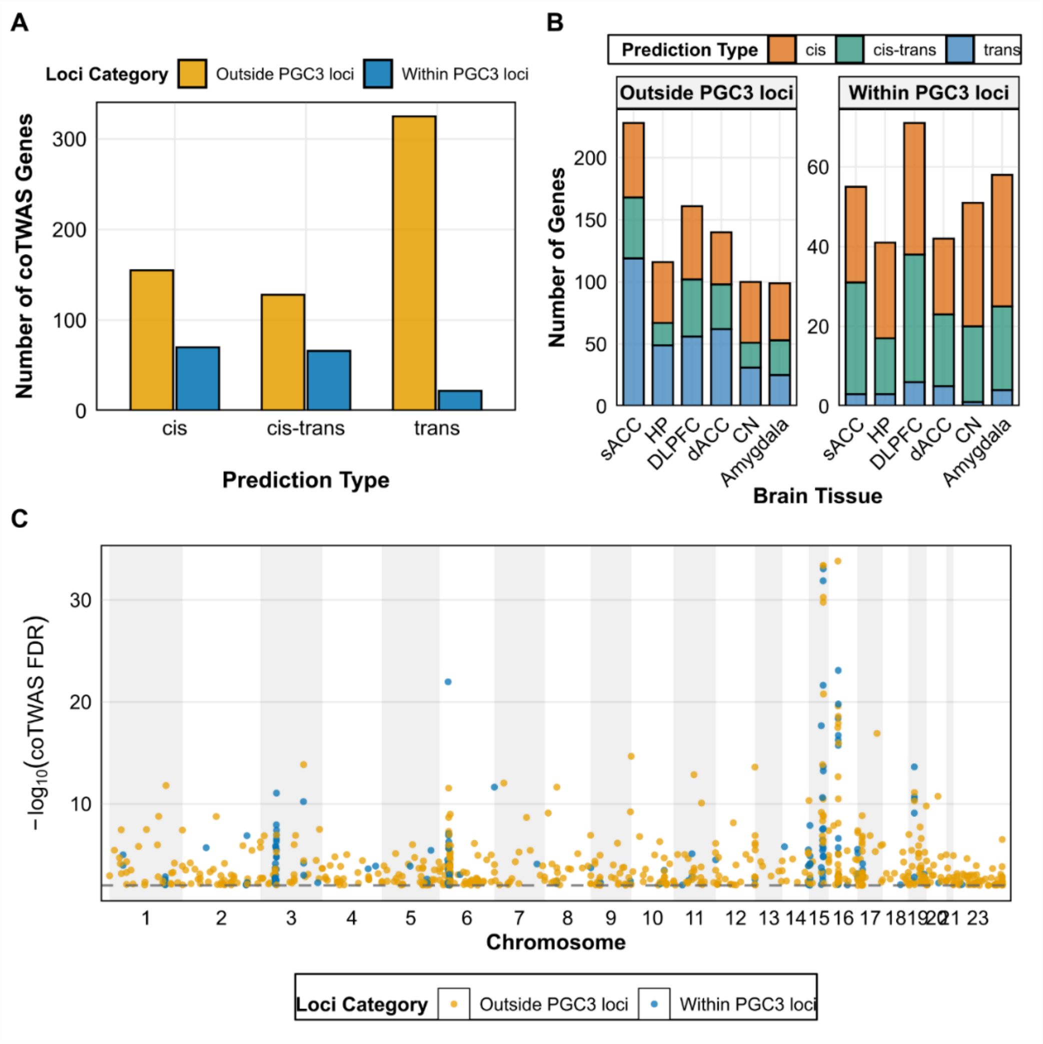

Figure 5. Distribution of coTWAS associations within and outside PGC3 schizophrenia loci. *A) Number of significant coTWAS genes stratified by prediction type (cis, cis–trans, trans) and their genomic position relative to genome-wide significant PGC3 loci. B) Regional distribution of coTWAS genes across six brain tissues, shown separately for genes outside (left) and within (right) PGC3 loci. C) Manhattan plot showing the chromosomal distribution of coTWAS-significant genes (–log□□ FDR) color-coded by locus category. Blue points denote genes within PGC3 loci and yellow points those located outside.*

outside known peaks.

Taken together, these results demonstrate that our method successfully recovers established cis-driven associations while simultaneously extending discovery into new

trans-regulatory space. When results are unpacked by each of the analyzed regions, trans-only contributions remain below 50%, which is not inconsistent with the article the Reviewer suggested. We report these results on p. 29 of the revised manuscript. Figure 4 is included as Supplementary Figure 27, showing only the conditional analysis results. Figure 5 is the new Supplementary Figure 28.

6. Because of the nature of overlapped eQTL for nearby genes, the TWAS results are known to be inflated. Significant risk genes including overlapped test eQTL are likely not independent significant signals. These need to be accounted for in reporting the number of novel findings.

Response:

We thank the reviewer for this important comment. In response, we revised our pipeline and now present results from conditional analysis as our main framework.

Our revised models in a standard coTWAS meta-analysis (96,535 gene–tissue tests across PGC3 sites and brain regions) identified 800 significant genes at FDR < 0.01 (compared to 1,764 unique genes in the original analysis), trading the number of novel discoveries for their reliability.

In direct response to the present concern, we implemented a locus-level conditional analysis following the approach of Huckins et al. (2019, PMID: 30911161). We partitioned the genome into 315 regions, considering the whole 96,535 gene–tissue tests, and applied forward stepwise conditional testing. This procedure identified 766 unique significant genes at conditional FDR < 0.01 across 4,318 conditional tests, corresponding to 314 independent loci overall. At the regional level, we observed 199 loci in DLPFC and HP, 198 in dACC, 221 in sACC, 169 in amygdala, 162 in CN. Notably, 362 significant genes were shared between the classical meta-analysis and the conditional analysis (Figure 6). We report the conditional analysis results on page 24 and describe the corresponding methods on pages 54-55 of the manuscript.

In lieu of an equally powered replication cohort, we performed a leave-one-site-out replicability analysis across the 62 PGC3 cohorts. For each conditionally significant gene–tissue pair, we assessed two measures of robustness: (a) the concordance of effect directions between the leave-one-out and held-out site models, and (b) the correlation (R^2) of effect sizes across sites. Empirical null distributions were generated by permuting held-out effects, and genes were retained as replicable if both concordance and R^2 exceeded the 95th percentile of the null distribution. This stringent approach reduced the final set to 556 unique, independent, and replicable signals (one-tailed empirical $p < 0.05$). We report the results and methods of this analysis in the manuscript on pages 24 and 55, respectively. In addition, we include a new Supplementary Figure 18 showing the cross-validated coTWAS genes.

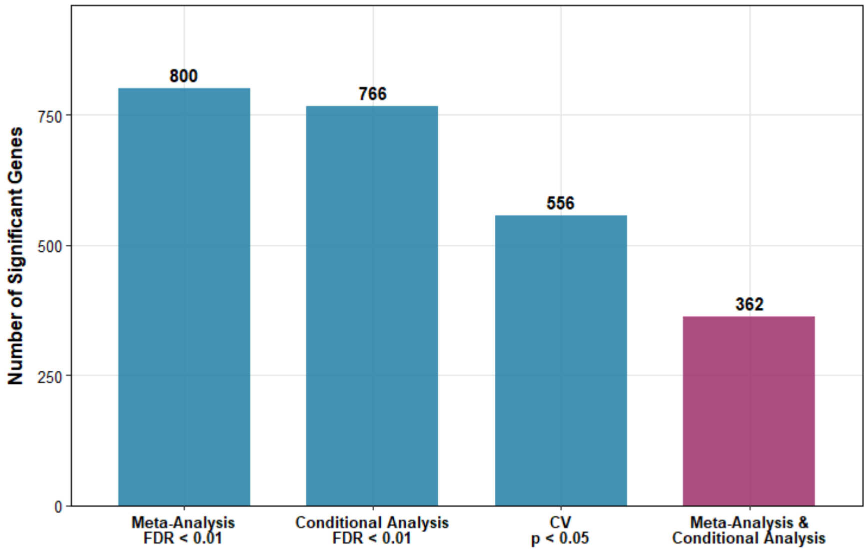

Figure 6. Reduction of inflated TWAS signals by conditional and replicability analyses. *Number of significant genes identified by different procedures: standard meta-analysis, conditional analysis, leave-one-out CV across PGC sites, and overlap between meta-analysis and conditional analysis.*

Taken together, our revised results represent signals that are less likely to be artifacts of local correlation and more likely to reflect biologically meaningful associations, as also shown in our CV framework.

7. It is shown that Mendelian Randomization and TWAS framework are aiming to test the same causal genetic effects that are mediated through gene expression (PMID: 32737316). It is expected that both analyses will obtain a large number of overlaps. So, it is not true that the authors say MR is an independent method. The authors need to describe clearly how MR is conducted, and how the instrument variables (eQTL) are determined.

Response:

We thank the reviewer for raising this important point. We agree that Mendelian Randomization (MR) and TWAS are conceptually related, as both test whether genetic

effects on complex traits are mediated through gene expression. We intended to use MR findings as a comparison, since the two frameworks differ in their statistical implementation and in how they handle causal inference.

We also clarify that we did not perform a new MR analysis in this study. Instead, we compared our coTWAS results against those reported in the large-scale PGC3 MR study (PMID: 35396580). Thus, in our manuscript MR is used solely as an external reference point derived from a robust published study, rather than as an independent replication of TWAS. We have now explicitly clarified this procedure in the revised main text (pp. 28-29).

Reviewer #2 (Remarks to the Author):

The manuscript by Rossi et al. introduced two novel trans-eQTL models, INGENE and MODULE, designed to enhance gene expression predictions in schizophrenia (SCZ). These models leverage co-expression networks to capture trans-regulatory effects, which are hypothesized to account for a substantial portion of gene expression heritability. The study integrates these trans-models with cis-based models (CIS and EpiXcan) to improve predictive accuracy and identify novel SCZ-associated genes. It seems to be an important innovation targeting a broader regulation force of gene expression. However, several concerns exist:

1. The manuscript employs relatively loose statistical thresholds (adjusted $R^2 \geq 0.01$ and p-value < 0.05) for model/signal selection, which increases the likelihood of false positives. Why multiple test correction not considered for the gene prediction part? I understand that it is a common practice in several other existing tools, but dealing with trans-regulation, the risk of false positives may further increase with the dramatically increased number of tests. At least, at this stage, I'd love to see how many of the signals can survive multiple test correction.

Response:

We thank the reviewer for raising this important point. The strength of our approach lies in its validation in independent external cohorts (CMC and GTEx), which we view as the most rigorous safeguard against false positives. In addition to the original pipeline design, we now explicitly apply cross-dataset training validation for our *trans*-based models (as requested in point 6), ensuring that only models reproducible across independent cohorts are retained. Importantly, this cross-dataset validation step is unique to our framework: other *trans*-informed methods (e.g., MOSTWAS [PMID: 33684137] and BGW-TWAS [PMID: 32961112]) have not applied alternative training thresholds. However, we note that it is important to keep the same thresholds used in previous TWAS publications to obtain a legitimate benchmark.

To directly address the reviewer's concern, we quantified the number of genes that survive Bonferroni and FDR corrections at different thresholds to our INGENE and MODULE prediction models in each brain region (Figure 7).

For MODULE, the impact of correction was modest: survival rate remained very high within regions, with ~96-97% of genes surviving under FDR thresholds and ~91-98% under Bonferroni.

For INGENE, ~71-80% of the genes were retained under FDR thresholds and ~52-65% under Bonferroni, depending on the region.

We would like to stress that correcting for multiple comparisons in the training set is still prone to overfitting, even in a CV setting (as also shown by the high survival rates in this analysis). Therefore, the replication in the independent testing datasets is the most robust approach. For these reasons, we retained the standard threshold ($p < 0.05$, adjusted $R^2 \geq 0.01$) in the results.

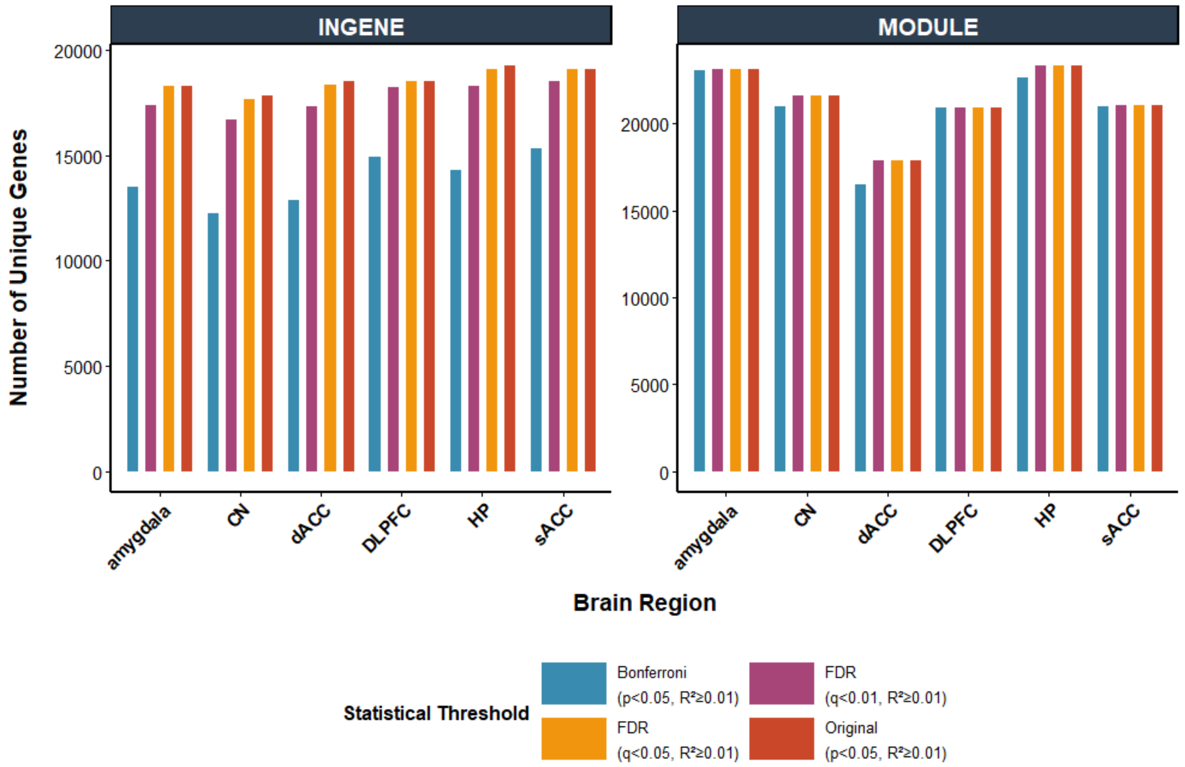

Figure 7. Number of unique genes retained by INGENE and MODULE under different statistical thresholds. *Number of unique genes predicted by INGENE (left) and MODULE (right) across six brain regions (amygdala, CN, dACC, DLPFC, HP, sACC). Bars represent results under four statistical thresholds: Bonferroni correction ($p < 0.05$, $R^2 \geq 0.01$), FDR $q < 0.05$ ($R^2 \geq 0.01$), FDR $q < 0.01$ ($R^2 \geq 0.01$), and the original threshold ($p < 0.05$, $R^2 \geq 0.01$).*

2. The manuscript does not explicitly mention whether DNA genotype data were compared with RNA-seq genotype data to verify sample identity in their preprocessing. This step is critical in studies involving both genotypic and transcriptomic data to ensure that samples are correctly matched and to avoid errors in downstream analyses.

Response:

We thank the reviewer for raising this important point. We confirm that the genotype–RNA-seq concordance check was performed to verify sample identity and exclude possible sample swaps. Specifically, as part of the SPEAQeasy pipeline used by the Lieber Institute for Brain Development (LIBD), single nucleotide variants (SNVs) are extracted from the RNA-seq data and compared against the DNA genotype calls for the corresponding donor. Concordance is assessed using standard tools such as *bcftools*, and any mismatches indicative of potential sample swaps are flagged and corrected or excluded. This quality control procedure is routinely applied at the LIBD to all datasets combining genotypic and transcriptomic information (see SPEAQeasy documentation: https://research.libd.org/SPEAQeasy-example/swap_speaqeasy.html).

We have now added a statement in the Methods section (p. 41), explicitly clarifying that this concordance check was performed as part of data preprocessing.

3. Regarding the CIS model's genetic variant selection, please clarify if the ±1 Mb window is defined relative to the transcription start site (TSS) or the gene body. This ambiguity could affect the interpretation of cis-eQTL boundaries and should be explicitly addressed.

Response:

We thank the reviewer for pointing out this ambiguity. In our analysis, the ±1 Mb cis-window was defined relative to the TSS of each gene. To ensure strand-specific accuracy, we identified the TSS as the 5′ end of the annotated gene: the start coordinate for genes on the positive strand and the end coordinate for genes on the negative strand.

We note that this TSS is an operational proxy rather than the exact biological TSS. Many genes have multiple transcripts and alternative promoters, but for large-scale eQTL mapping it is standard practice to use the 5′ end of the gene annotation as a consistent anchor point. This is the same convention adopted by widely used tools such as tensorQTL (PMID: 31675989). We have revised the Methods section (p. 45) to make this explicit.

4. On Page 19, the manuscript focuses on MODULE's functional enrichment of SCZ signals without adequately explaining why INGENE is not discussed.

Response:

We understand that some clarification is needed on this point. The reason we did not emphasize INGENE in the functional enrichment analyses depends on this method's design. Indeed, INGENE imputes target gene expression using the cis-genetically regulated expression of its co-expression partners, rather than directly leveraging SNP predictors. This procedure is conceived so that the genetic signal underlying INGENE predictions is mediated through the cis-regulation of partner genes. In practice, when the INGENE model is compared against GWAS signals, their enrichment profile is expected to mirror that of classical cis-based methods (CIS and EpiXcan). Indeed, as shown in Figure 8, the relationship between GWAS effect sizes (absolute log□ odds ratio) and cis-SNPs of coexpression partners in INGENE closely resembles the pattern observed for CIS and EpiXcan models, with relatively low correlation compared to MODULE. This finding is consistent with the fact that INGENE SNPs are cis-eQTLs after all, although we evaluate

their effects in trans on predicted genes. We added a sentence on p. 20 to better explain INGENE exclusion from this analysis.

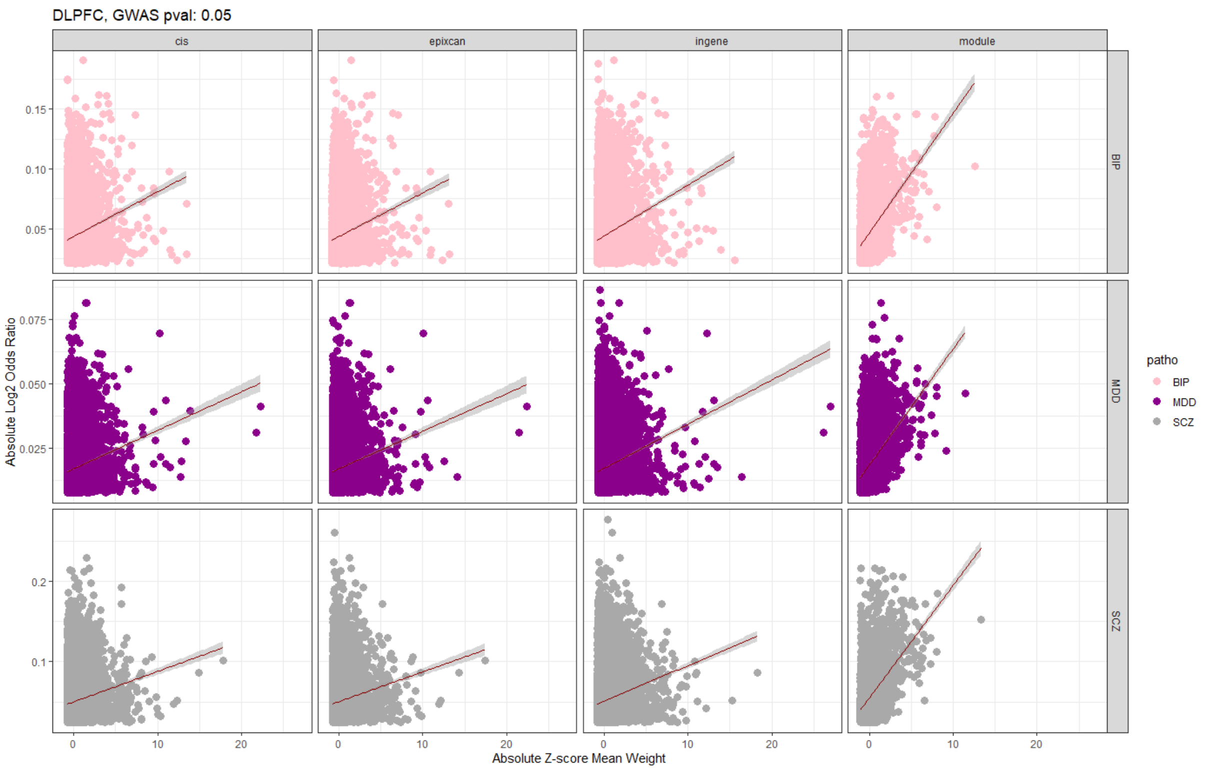

Figure 8. Scatterplot of PGC3 (p-value < 0.05) SNP log(OR) and mean weight absolute value values in CIS, EpiXcan, INGENE and MODULE. *The x-axis shows the absolute Z-score mean weight of each SNP, while the y-axis represents the absolute value of the log2 of PGC3 Odd Ratios.*

5. When assessing replicability of the cis and trans models, the authors counted the number of genes that can be predicted in GTEx and CMC, also presented the number of genes shared by the two cohorts, and their Pearson correlation. However, they should show the percentage of sharing in the total genes for the three cohorts. My impression is that the % is not that high, meaning that there are many genes predicted only in one specific data. That could indicate the instability of prediction.

Response:

We thank the reviewer for this suggestion. In the revised analysis of complementarity between CMC and GTEx using LIBD-trained models, replicability was quantified in two ways: (i) the correlation of prediction accuracy between the two validation cohorts (Supplementary Figure 6, scatterplots, left), and (ii) the proportion of genes consistently predicted in both cohorts relative to the total number predicted in at least one cohort (Supplementary Figure 11, Venn diagrams, right).

For the baseline CIS and EpiXcan models, replicability between CMC and GTEx ranges from ~32% to 47% of predicted genes, with correlations up to r = 0.43-0.68. INGENE and MODULE were filtered by cross-dataset training replicability (see details in response # 6). After this filtering, overlap fractions were ~77-88% across regions, and cross-cohort correlations reached r = 0.17-0.52 for INGENE and r = 0.14-0.17 for MODULE. Although

these correlations are lower than those observed for *cis*-only models, this is expected for *trans*-prediction relative to *cis*-prediction, due to leveraging a larger number of SNPs with smaller effect sizes.

We have reported these new results in the main manuscript (p. 18) and updated Supplementary Figure 11 accordingly.

6. Since we are talking about the genetic regulators of gene expression, they should be stable across cohorts. The better test of replicability would be testing the weights/effect sizes of each gene's prediction models learned from three cohorts. If the training on different brain collections obtained consistent prediction models, I would be more convinced about their prediction. With that, they would need to train the models from three cohorts. It is a non-trivial job, but critical to prove their points about the reliable use of trans-regulation information. I would recommend that they take the time to do the right, diligent work.

Response:

We thank the reviewer for this important suggestion. We fully agree that demonstrating replicability across independent cohorts is critical to establish confidence in the use of *trans*-regulatory information. In response, we undertook an extensive series of new analyses that both test cross-cohort stability and reinforce the rigor of our pipeline.

(1) Training performance across models and regions.

As a first step, we systematically compared raw training performance across models (CIS, EpiXcan, INGENE, MODULE) and across all available datasets (LIBD, GTEx, CMC) and brain regions (CMC models were limited to DLPFC and ACC, while EpiXcan lacked amygdala RoadMap functional annotations). This design provided a comprehensive, three-way training framework spanning multiple cohorts and brain regions.

We first considered the statistical power of each dataset for eQTL identification. Sample sizes for each dataset and region are shown in Fig. 9A, ranging from as few as 116 samples in CMC amygdala to nearly 600 in LIBD DLPFC, highlighting substantial heterogeneity. These sample-size differences likely reduce the generalizability of CV training performance and contribute to variability across cohorts, a well-documented limitation of CV in settings with unbalanced sample sizes (PMID: 28655633; PMID: 31697686).

When comparing the number of genes with predictive models (Fig. 9B), we observed marked differences in model stability and performance (Fig. 9C). Despite the larger effect size per SNP, CIS models showed extreme variability, with mean CV adjusted R^2 between predicted and observed gene expression ranging from 0.11 in CMC to 0.78 in GTEx, with very wide error bars. EpiXcan displayed similar variability, with mean adjusted R^2 spanning 0.10–0.32 across datasets. By contrast, INGENE and MODULE achieved more stable performance: INGENE reached mean adjusted R^2 of 0.34 in CMC, 0.30 in LIBD, and 0.10 in GTEx, while MODULE yielded 0.15–0.20 across datasets, all with much narrower error bars.

Taken together, Fig. 9 (panels A–C) shows that predictive models could be trained across independent datasets, with overall strong performance. Cis-based models provide a useful

benchmark, but their variability suggests a sensitivity to sample-size differences, an aspect we further consider below. Importantly, INGENE and MODULE performed at least as well as cis models, without showing reduced stability. We added these results to p.13 of the manuscript and to pp. 8-9 of the Supplementary Notes (Section 2.1), with methods on pp. 49-50 in the manuscript. The corresponding plots are presented as new Supplementary Figure 3.

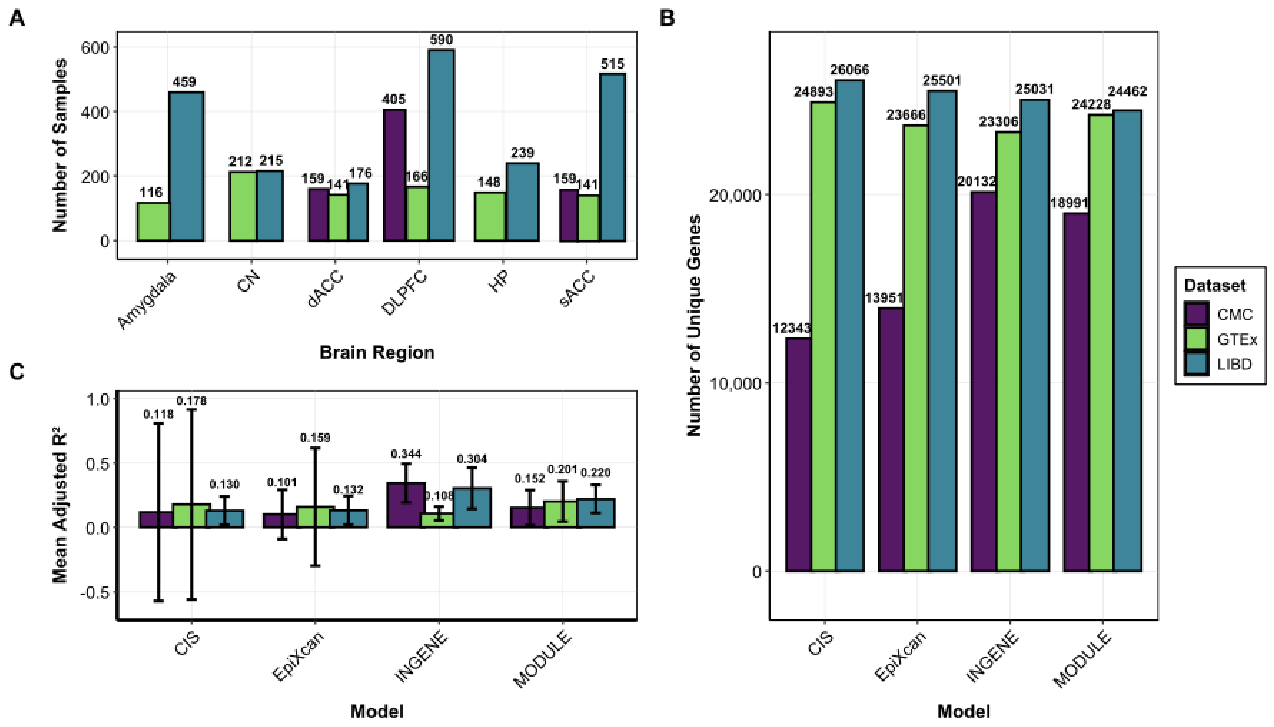

Figure 9. Training performance of predictive models across cohorts and brain regions. *(A) Sample sizes available for training across brain regions in CMC, GTEx, and LIBD, highlighting substantial variability in statistical power. (B) Number of unique genes within predictive models across cohorts for each framework (CIS, EpiXcan, INGENE, MODULE). (C) Mean cross-validated adjusted R^2 values across datasets, showing high variability for CIS and EpiXcan, and greater stability for INGENE and MODULE.*

(2) Sample-size sensitivity of cis-based predictors.

The results highlight three key points (Fig. 10A–D):

1. **CIS is highly prone to instability.** For CIS, cross-cohort performance differences scaled strongly with the ratio of training sample sizes ($R^2 = 0.476$, $p = 0.019$; Fig. 10A), confirming that CIS predictions are sensitive to sample-size heterogeneity. This is evident at the gene level: in the CMC–LIBD DLPFC comparison, where sample sizes are similar (refer to Figure 8A), CIS retained ~53% of overlapping genes. In other regions with more unbalanced cohorts, retention dropped markedly in proportion to the sample-size ratio (Fig. 10B). Across all regions, CIS retention ranged from ~25% to 53%.
2. **EpiXcan is comparatively more stable.** In contrast, EpiXcan showed a non-significant association between sample-size ratio and performance differences ($R^2 = 0.033$, $p = 0.62$; Fig. 10C). In balanced comparisons (CMC–LIBD DLPFC), retention exceeded that of CIS, and in unbalanced comparisons the drop-off was less

pronounced (Fig. 10D). Across regions, EpiXcan retention ranged from ~35% to 60%, consistently outperforming CIS

3. **Overlap across cohorts remains limited.** Despite being more stable than CIS, EpiXcan still shows modest gene-level replicability across datasets. Even under balanced sample sizes (e.g., CMC–LIBD DLPFC), only about half of overlapping genes were retained, and in most other cohort pairs both CIS and EpiXcan lost 70-85% of genes.

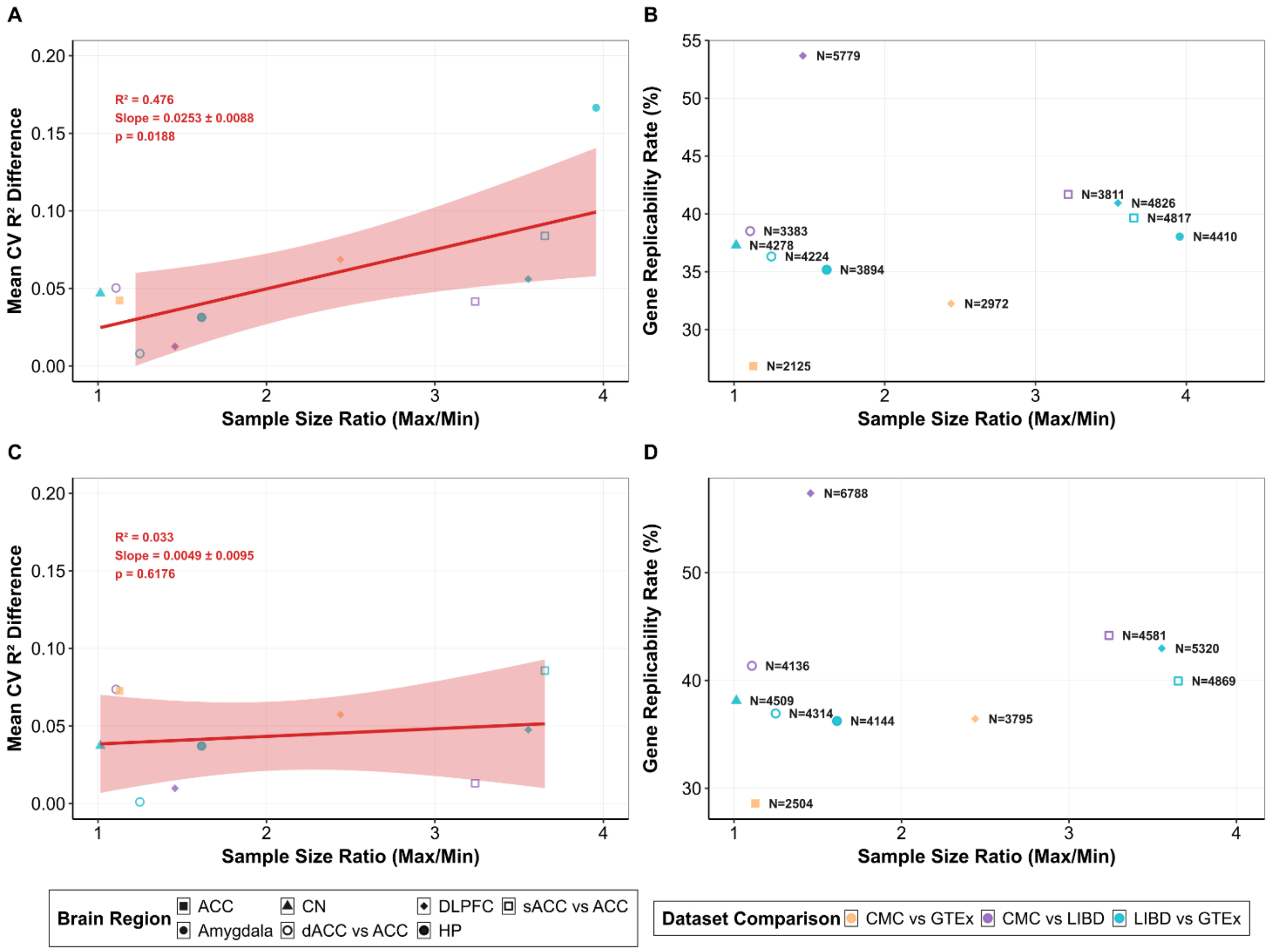

Figure 10. Sample-size sensitivity of CIS and EpiXcan predictors*. (A) Relationship between sample-size ratio (maximum/minimum) and cross-cohort performance differences for CIS models, showing strong sensitivity to heterogeneity ($R^2 = 0.476$, $p = 0.019$). (B) Gene-level replicability rates for CIS across cohort comparisons, with retention declining as sample-size imbalance increases. N represents the number of genes that pass the cross-dataset filtering threshold. (C) Relationship between sample-size ratio and performance differences for EpiXcan models, showing no significant association ($R^2 = 0.033$, $p = 0.62$). (D) Gene-level replicability rates for EpiXcan across cohorts, consistently outperforming CIS but still showing limited overlap between datasets. N represents the number of genes that pass the cross-dataset filtering threshold.*

These analyses are reported on p.13 of the manuscript and in Supplementary Notes on pp. 9-10 (Section 2.2). The results shown on Figure 10 are reported ad Supplementary Figure 4.

7

For these reasons, we adopted a balanced strategy. CIS and EpiXcan predictions were retained in full for downstream coTWAS analyses, ensuring comparability with previous

TWAS studies. In contrast, our trans-aware models (INGENE and MODULE) were subjected to more stringent replication filters, retaining only genes reproducible across cohorts. This guarantees that our novel methods are benchmarked under the most rigorous conditions, while avoiding penalization of established *cis*-based predictors. Given that trans-models identify numerous otherwise unpredicted genes, to reduce the multiple test burden and false positive rate, it is desirable to limit the analysis to the most reliable predictions.

(3) Cross-dataset replication for trans-aware models.

Our original pipeline had two valuable features: i) we implemented a train-test strategy with three independent datasets, and ii) as shown above, the LIBD is the best-powered sample to be used as a main training set. To directly address the reviewer's request, we implemented a cross-dataset replication strategy for INGENE and MODULE while preserving the integrity of our original pipeline (LIBD as training; GTEx and CMC as independent validation). Indeed, using GTEx and CMC for training models would leak information from the testing datasets and invalidate our replication analyses. Therefore, we operated by eliminating gene prediction models obtained from LIBD whenever they could not be reliably trained in GTEx and CMC, while also keeping the replication filters when models were tested and not trained in GTEx and CMC. This way, model generation in LIBD was not affected, while the multiple comparison burden and consequent false positive rate decreased.

Concretely, for each gene we correlated imputed gene expression values from GTEx and CMC with LIBD predictions, with all correlations computed across the same LIBD individuals. This approach eliminates sample-size confounding and provides a direct measure of cross-cohort consistency in estimated effect sizes.

Across brain regions, both MODULE (Fig. 11 A-B) and INGENE (Fig. 11 C-D) retained substantial replicable subsets of cross-dataset genes. Panel A shows that the number of replicable genes was consistently large across regions, ranging from ~6,900 to ~14,400 depending on the dataset and brain region. Panel B illustrates that the distributions of correlation coefficients between predicted and observed expression was skewed towards positive values, indicating that most replicable genes capture genuine cross-cohort signal.

The majority of the gene models trained in LIBD survived when we tested them in GTEx. Panel C shows that INGENE retained 65–85% of GTEx-validated genes depending on region, while MODULE retained ~55–75%. Notably, where both GTEx and CMC datasets were available, survival rates followed nearly identical trends, arguing against dataset-specific idiosyncrasies as a main driver of performance. Finally, we examined retention at variable thresholds of Pearson's correlation between predicted and observed gene expression. Panel D demonstrates a gradual decline in survival rates as correlation thresholds increase, confirming that replicability holds across a spectrum of stringency levels rather than being driven by a small set of marginal cases.

By anchoring replicability to LIBD as the common reference, this strategy ensures that GTEx and CMC remain untouched to keep their intended role as external validation datasets. At the same time, it provides a stringent filter for trans-aware models, retaining only those genes that demonstrate cross-cohort stability in effect sizes not only during testing but also during training. We included the full results of this cross-dataset replication

in the Supplementary Notes on pp. 8-9 (Section 2.1), and the findings (Figure 11 panels A,C) are shown in Supplementary Figure 2.

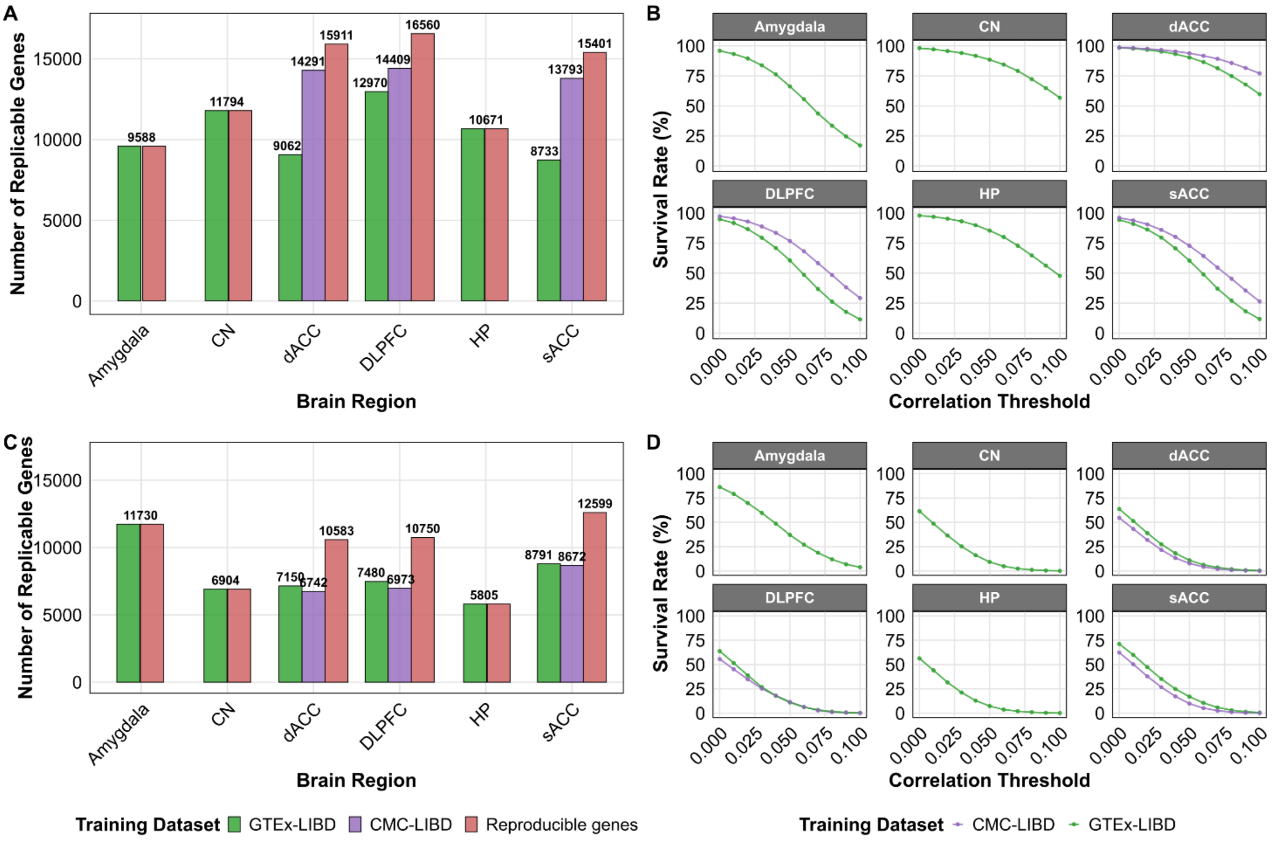

Figure 11. Cross-dataset replication of trans-based INGENE and MODULE models*. (A) Bars show the number of genes passing the replication criterion (r > 0 between external and LIBD-predicted expression) in GTEx → LIBD (green), CMC → LIBD (violet), and the total number of reproducible genes (red). (B) Survival rates of MODULE genes at increasing Pearson correlation thresholds between predicted and observed expression in testing dataset GTEx, showing positive skew consistent with true signal. (C) Number of replicable INGENE genes across brain regions in GTEx and CMC relative to LIBD. (D) Survival rates of INGENE genes across varying correlation thresholds, confirming replicability under progressively stringent criteria in testing dataset.*

Along with my 5th point above, comparing CIS, INGENE, and MODULE performance regarding their reproducibility is the most important part of this paper.

I see that they are discovering more genes, and some of them have meaningful functional implications. However, whether they are true positives needs more thoughtful validation. Having a small portion uncovered from different data is not convincing. Showing the effect sizes from different models are similar; or heritability from cis- and trans- regulations, are all not direct proof of model reproducibility.

The coTWAS identified 1,515 novel genes. Then, how do we know that they are valid findings.

Response:
We thank the reviewer for raising this important concern. We agree that demonstrating the robustness of novel associations is critical. In the revised manuscript, we implemented multiple complementary validation steps to ensure that our findings are not artifacts of local correlation or overfitting.

1. **Locus-level conditional analysis.** To address the risk of inflated discoveries from overlapping eQTLs among nearby genes, we replaced the previous coTWAS meta-analysis with a locus-level conditional analysis (PMID: 30911161). This step reduced the initial 1,764 significant genes to 766 independent associations across 314 loci, thereby prioritizing signals that survive after accounting for local correlation. We now present these 766 genes as our main gene set. We report results and methods in the main text (p.24 and 54-55 respectively).

2. **Replication across PGC3 cohorts.** Since no independent SCZ cohort of comparable size is available for true replication, we further assessed stability using a leave-one-site-out procedure across the 62 contributing cohorts (methods described on p. 55 of the manuscript). This analysis does not constitute a formal replication set but serves to evaluate how often associations generalize across subsets of the data. Genes were retained only if their effects were replicated in held-out sites with consistent direction and effect-size correlation beyond the 95th percentile of null distributions. This approach yielded 556 unique, independent, and replicable signals (results added on p. 24; see Figure 6 in this rebuttal, Supplementary Figure 18 and Supplementary Table 7), indicating that a large majority of the 766 genes discovered demonstrate reliability in stringent settings.

Taken together, these validation steps indicate that the reported novel genes are supported by independent and replicable signals rather than artifacts of model inflation.

7. Page 14 and Figure 3, they compared their CIS model to EpiXcan. If I understand it correctly, the only difference of these two models is the training data. Is that right? Both only used cis-eQTL. Please clarify.

Response:
While both CIS and EpiXcan rely exclusively on cis-eQTLs (±1 Mb of the transcription start site), their difference is not limited to the source of training data. CIS uses a standard elastic-net trained on cis variants without priors (PMID: 26258848), whereas EpiXcan (PMID: 31444360) integrates functional annotations from epigenomic annotations from the Roadmap Epigenomics Consortium (PMID: 25693563) as penalization priors.

In this revision, we retrained EpiXcan across all LIBD brain regions, ensuring a fair comparison with the CIS model trained under the same framework. As shown in Figure 12 panels A-B, the vast majority of genes (~87%) are predicted by both methods, but each also contributes unique models (~6% CIS-only and ~8% EpiXcan-only), highlighting their

complementarity. At the performance level, EpiXcan achieves consistently higher predictive accuracy: across shared genes, the mean cross-validated R^2 is 0.153 for EpiXcan versus 0.140 for CIS (~9% relative gain, Figure 12 panel C).

At the gene level, results are mixed (Figure 2D). For instance, in DLPFC, CIS predicted 14,324 genes and EpiXcan 14,442, with 13,822 shared. Among shared genes, EpiXcan yielded higher R^2 for 6,994 genes (48.8%), while CIS was superior for 6,411 genes (44.8%), with 919 (6.4%) tied. Similar proportions were observed across CN, dACC, HP, and sACC. Importantly, the magnitude of improvement favors EpiXcan: when EpiXcan outperforms CIS, the gains are larger (e.g., mean ΔR^2 = 0.0167 to 0.0228 across regions) than the modest gains of CIS when it is better (mean ΔR^2 = +0.0066 to +0.0434). Both models also predict unique subsets of genes (e.g., 502 CIS-only vs. 620 EpiXcan-only in DLPFC; 1,955 vs. 2,295 in HP).

Thus, while EpiXcan is generally the strongest of the two methods in aggregate, CIS remains competitive and can be advantageous for subsets of genes or regions. For this reason, we deliberately retained both CIS and EpiXcan in our pipeline: this strategy maximizes gene coverage, ensures fairness across regions, and avoids biasing discovery toward a single cis-only predictor.

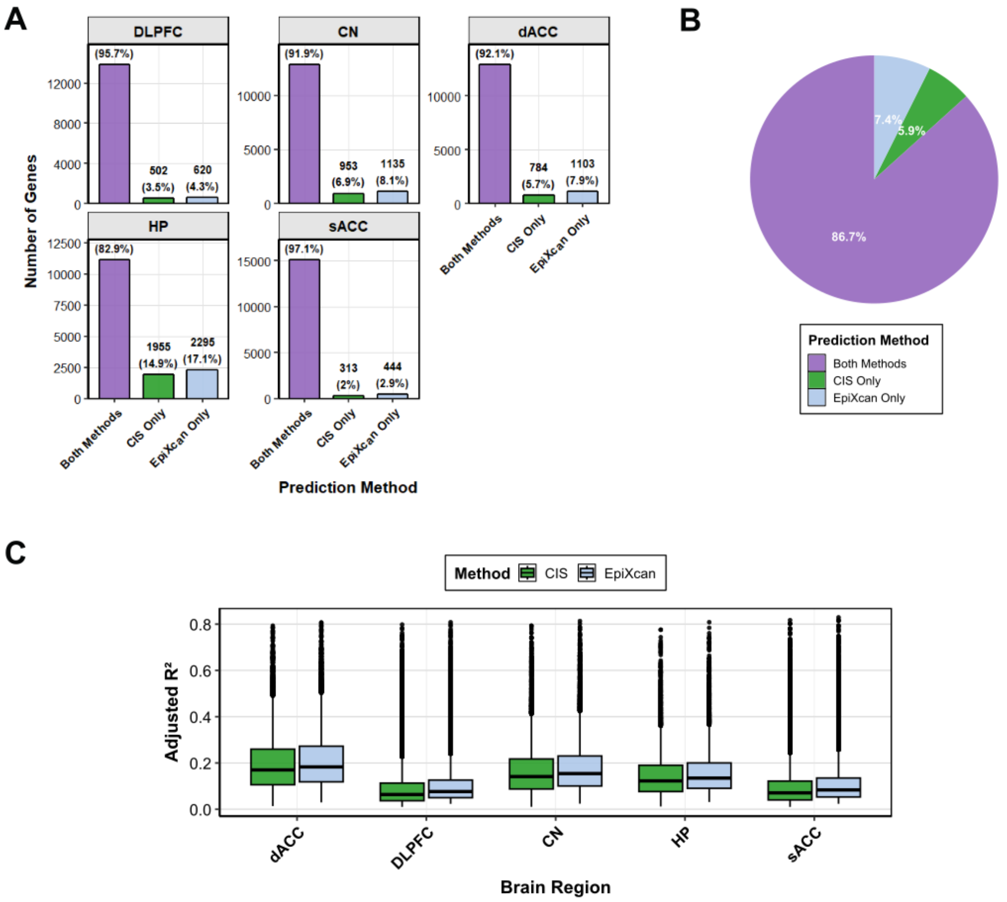

__Figure 12. Comparison of CIS and EpiXcan prediction models across brain regions.__ (A) Overlap of predicted genes between CIS (green) and EpiXcan (blue) across five LIBD brain regions. The vast majority of genes (purple) are predicted by both methods. (B) Aggregate distribution across all regions. (C) Distribution of cross-validated prediction accuracy (adjusted R^2) across LIBD brain regions. EpiXcan (blue) achieves consistently higher average predictive performance compared to CIS (green).

8. Page 18, “40% MODULE trans-SNPs were GTEx cis-eQTLs, predicting 5,821 and 19,276 eGenes.” Quite some information was missing in this sentence. I don’t understand what they try to say. Trans-SNPs in MODULE are also cis-SNPs of those eGenes in GTEx, two brain regions?

Response:
We thank the reviewer for highlighting that this sentence was unclear. Our intention was to show that many SNPs identified by MODULE as trans-regulators also act as cis-eQTLs for other genes (eGenes) in the GTEx QTL resource (PMID: 32913098). In other words, these trans-SNPs have evidence of a cis effect on a nearby gene while also exerting trans effects on other genes. We used results from an independent analysis of GTEx data to avoid double-dipping in our analysis. We have revised the text (p. 19) to clarify that the reported numbers correspond to each brain region analyzed (Supplementary Figure 12).

9. If they just compare their coTWAS results to PGC3 MR gene list, which is based on cis-regions, the comparison is not meaningful. That cannot be used to prove their coTWAS based on trans- regulation, as they target very different regulations.

Response:
We agree that the comparison between our coTWAS results and the PGC3 MR findings (PMID: 35396580) is inherently limited, as the latter are derived solely from genome-wide significant cis-eQTL instruments, whereas our framework also incorporates trans-eQTL–mediated regulation. Consequently, the overlap between the two approaches cannot be interpreted as validation of trans-based findings. Instead, our intent was to provide a comparison of our novel findings with previously published findings.

We have revised the manuscript to emphasize this distinction. Specifically, we now clarify that MR comparisons do not serve to validate trans-predictions but rather provide complementary evidence that our coTWAS signal is consistent with prior large-scale causal inference analyses. This limitation is now explicitly acknowledged in the Discussion (p. 37).

In summary, this is an exciting project. But I am not completely convinced about the reproducibility of their results. Additional tests and validations are required.

Response:
We thank Reviewer#2 for their appreciation and for the constructive comments provided.

Reviewer #2 (Remarks on code availability):

cannot access, without permission.

Response:
We apologize for the restricted access in the previous submission. In this revised submission, we provide the complete pipeline code in a dedicated folder, including all scripts

required to reproduce our analyses. Upon publication the source code will be made openly accessible without restrictions, in line with the journal’s policy on reproducibility.
